# Synthesis of 4-Hydroxyquinolines as Potential Cytotoxic Agents †

**DOI:** 10.3390/ijms23179688

**Published:** 2022-08-26

**Authors:** Oszkár Csuvik, Nikoletta Szemerédi, Gabriella Spengler, István Szatmári

**Affiliations:** 1 Institute of Pharmaceutical Chemistry, University of Szeged, Eötvös u. 6, H-6720 Szeged, Hungary; 2 Department of Medical Microbiology, Albert Szent-Györgyi Health Center and Albert Szent-Györgyi Medical School, University of Szeged, Semmelweis u. 6, H-6725 Szeged, Hungary; 3 Stereochemistry Research Group, Eötvös Loránd Research Network, University of Szeged, Eötvös u. 6, H-6720 Szeged, Hungary

**Keywords:** Conrad–Limpach reaction, 4-hydroxyquinoline, modified Mannich reaction, Knoevenagel condensation, cytotoxic effect, selective toxicity towards MDR cancer

## Abstract

The synthesis of alkyl 2-(4-hydroxyquinolin-2-yl) acetates and 1-phenyl-4-(phenylamino)pyridine-2,6(1*H*,3*H*)-dione was optimised. Starting from 4-hydroxyquinolines (4HQs), aminomethylation was carried out via the modified Mannich reaction (*m*Mr) applying formaldehyde and piperidine, but a second paraformaldehyde molecule was incorporated into the Mannich product. The reaction also afforded the formation of bisquinoline derivatives. A new 1*H*-azeto [1,2-*a*]quinoline derivative was synthesised in two different ways; namely starting from the aminomethylated product or from the ester-hydrolysed 4HQ. When the aldehyde component was replaced with aromatic aldehydes, Knoevenagel condensation took place affording the formation of the corresponding benzylidene derivatives, with the concomitant generation of bisquinolines. The reactivity of salicylaldehyde and hydroxynaphthaldehydes was tested; under these conditions, partially saturated lactones were formed through spontaneous ring closure. The activity of the derivatives was assessed using doxorubicin-sensitive and -resistant colon adenocarcinoma cell lines and normal human fibroblasts. Some derivatives possessed selective toxicity towards resistant cancer cells compared to doxorubicin-sensitive cancer cells and normal fibroblasts. Cytotoxic activity of the benzylidene derivatives and the corresponding Hammett–Brown substituent were correlated.

## 1. Introduction

4-Hydroxyquinolines or 4-quinolones possess a variety of pharmacological activities. Some of the substituted 2-(4-hydroxyquinolin-2-yl) acetates are rather active compounds. The 7-methoxy derivative attached to a propranolamine chain proved to be a more potent β-adrenergic receptor blocker in vivo in a rat preparation than propranolol [1]. The ethyl ester of the 6-trifluoromethoxy derivative was reacted with hydroxylamine affording an *N*-hydroxyacetamide derivative, which was tested as a matrix metalloproteinase inhibitor [2]. The first 3-carboxyl-substituted 4-hydroxyquinoline with antibacterial effect was discovered serendipitously. It was an intermediate by-product in chloroquine synthesis, leading to the development of fluoroquinolone antibiotics [3]. The 2-carboxylic acid derivative of 4-hydroxyquinoline is kynurenic acid (KYNA), which is an endogenous metabolite produced in both humans and rodents; moreover, it is a potential neuroprotective agent [4].

Aminoalkylation is usually referred as Mannich aminoalkylation, which involves ammonia or amine, an aldehyde, and a substrate with an active hydrogen. The procedure was described by Carl Mannich by reacting ammonia, formaldehyde, and antipyrine [5]. However, Mannich’s study is subsequent to Mario Betti’s research [6,7], who reported the reaction of ammonia, benzaldehyde, and 2-naphthol yielding the aminoalkylated derivative of 2-naphtol (1-α-aminobenzyl-2-naphthol). The reaction mechanism of Mannich and Betti procedure is identical; though, the procedure has become known as Mannich reaction [8]. In recent years, the process has received increasing attention due to the variability of the components involved, the use of mild reaction conditions, and the potential biological activity of the end products [9,10]. Among the versatile pharmacological possibility of their application, a prominent field is anticancer therapy; specifically, using them as cytotoxic agents [11,12]. In the modified Mannich reaction (*m*Mr), the aminoalkylation of hydroxyquinolines can be achieved [13], since this moiety can be considered as an *N*-containing 1-naphthol analogue. The aminomethylation of rather similar scaffold, including 2-methyl-4-hydroxyquinoline and KYNA, has been performed [14,15,16,17]. The aim of this study was to test the reactivity of 2-(4-hydroxyquinolin-2-yl) acetates in *m*Mr, using piperidine as amine with formaldehyde or aromatic aldehydes. Further aim of our study was to develop tumour selective anticancer drugs that are potent against cancer cells and exert low toxicity towards normal cells. The compounds with selective toxicity may have less side effects because they target only cancer cells.

## 2. Results and Discussion

### 2.1. Synthesis

#### 2.1.1. Syntheses of 4-Hydroxyquinolines

**Scheme 1:**
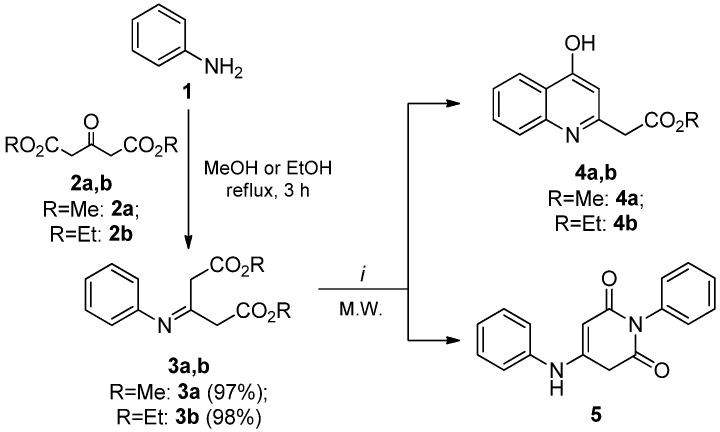
The synthesis of 4-hydroxyquinolines.

The synthesis of ethyl 2-(4-hydroxyquinolin-2-yl) acetate is known as the Conrad–Limpach reaction [18,19,20]. However, Rahn et al. revealed an alternative synthetic method [21], when 1-methoxy-1,3-bis(trimethylsilyloxy)-1,3-butadiene was reacted with 2-nitrobenzoyl chlorides followed by the hydrogenation of the condensation products resulting in substituted 2-(4-hydroxyquinolin-2-yl) acetates. In our study, the Conrad–Limpach reaction was used with some fine-tuned modifications (Scheme 1).

Aniline and dimethyl- or diethyl-1,3-acetonedicarboxylate were dissolved in methanol or ethanol, respectively. The mixture was treated under reflux conditions for 6 h, affording intermediate enamines **3a** or **3b**. The alcohol was evaporated by vacuum distillation, and the residue was dissolved in 1,2-dichlorobenzene.

According to the literature, ring closure requires high temperature for a short period of time. The boiling point of 1,2-dichlorobenzene is 180 °C, but the required temperature was higher. Therefore, the reaction was performed in closed, pressurised vials and heating in a microwave reactor, enabling a temperature higher than that of the boiling point of the solvent. Several conditions (*i*) were tested that are included in Table 1. After microwave irradiation (M.W.), the mixture was cooled in iced water and crystals formed were filtered out.

At lower temperatures, the presence of by-product **5** was observed, but its formation could be lowered by increasing the temperature. The critical reaction temperature was 245 °C, until then the side product could be perceived. However, according to the literature [22], 4 h at 120 °C, followed by a 2-h heating at 180 °C are the optimum conditions for the formation of **5**. In contrast to some earlier studies [23], our observation was that an additional acid catalyst (e.g., *para*-toluenesulphonic acid) did not afford the formation of the quinoline skeleton and only pyridinedione derivative **5** was detected.

#### 2.1.2. Reaction of 2-(4-Hydroxyquinolin-2-yl) Acetates with Paraformaldehyde and Piperidine

**Scheme 2:**
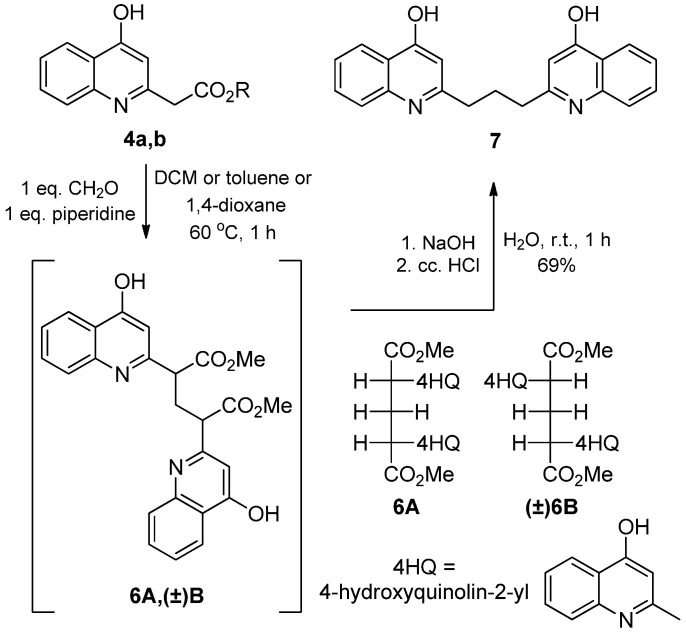
The reaction of 2-(4-hydroxyquinolin-2-yl) acetates with paraformaldehyde in the presence of piperidine.

Our first attempt was the aminoalkylation of **4a** via *m*Mr reacting piperidine and paraformaldehyde in dichloromethane under reflux (Scheme 2). After a 1-h reaction, formed crystals were filtered out. The crude ^1^H NMR spectrum showed that the product was not the desired 3-aminometylquinolinol. The spectrum was remarkably similar to that found by Tatemitsu et al. studying *meso*- and (±)-diphenylglutaric acid [24]. Valdéz-Camacho et al. also isolated the mixture of *erythro* and *threo* dimethyl 2,4-diphenylpentanedioate side products [25]. According to these observations, we assumed that the reaction afforded the mixture of *meso* compound **6A** and racemic compound **(±)6B** (Scheme 2). Our attempts to separate these compounds were unsuccessful, but in order to confirm the structures synthetically, decarboxylation was tested. The saponification of **4b** furnishes the 2-methyl derivative, i.e., quinaldine [18]. Note that similar bisquinolines have been synthesised and transformed via decarboxylation by Ayad et al. [26] applying NaOH. Accordingly, NaOH treatment was followed by neutralisation. In the ^1^H NMR spectrum of the isolated product, a quintet with 2 integrals at 2.12 ppm and a triplet with 4 integrals at 2.70 ppm were present. These multiplicities appear when a –(CH_2_)_3_– chain is located between aromatic rings [27], which is consistent with the structure of **7**.

**Scheme 3:**
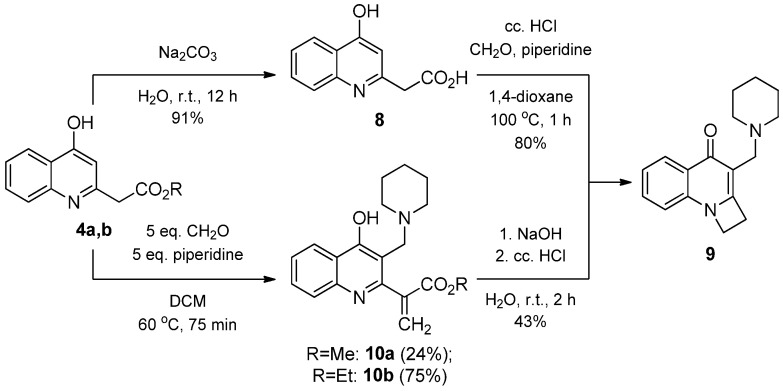
Synthetic routes to 1*H*-azeto [1,2-*a*]quinolin **9**.

The reaction in toluene and 1,4-dioxane afforded the bisquinoline derivatives as well. In order to gain an aminomethyl product, it was necessary to synthesise the carboxylic acid derivatives of the 4HQ acetates (**4a,b**) and then to transform them via *m*Mr. As mentioned, the saponification of **4a,b** with NaOH affords quinaldine; consequently, alternative reagents were tested. Acidic ester hydrolysis by cc. HCl furnished **8**, but after a few hours, spontaneous decarboxylation took place resulting in quinaldine. Immediately after isolation of **8**, without waiting for undesired decarboxylation, paraformaldehyde and piperidine were added, and the mixture was stirred in 1,4-dioxane for 1 h. The formed white solid was isolated and analysed. In the ^1^H NMR spectrum, two triplets were observed with 2-2 integrals, which requires two –CH_2_– neighbouring groups, and the signs of piperidine and the methylene bridge were also present. These were not consistent with the structure hypothesised previously, but the formation of **9** was assumed (Scheme 3).

In order to gain the stable 2-(4-hydroxyquinolin-2-yl)acetic acid (**8**), **4a** was treated with Na_2_CO_3_. The hydrolysis required a longer reaction, but a stable compound was formed. It was reacted with paraformaldehyde and piperidine in 1,4-dioxane, but no reaction occurred. The reaction was modified by first using cc. HCl followed by adding the other reagents (paraformaldehyde and piperidine). A new spot appeared on the TLC, and the isolated product was identical to that synthesised previously (compound **9**).

The aminomethylation presumably took place on **8**; therefore, *m*Mr of **4a** was repeated with an increased amount of paraformaldehyde and piperidine using 5 equivalents each. The reaction was carried out in dichloromethane stirring for 75 min. After isolation, the corresponding spectra were studied. The ^1^H NMR spectrum seemingly indicated the 3-(piperidin-1-ylmethyl) derivative of **4a**, since the signs of piperidine and the methylene bridge could be observed. However, the MS spectrum showed a higher molecular weight; therefore, structure **10a** was presumed. The synthesis of **10b** was carried out in a similar manner providing a higher yield compared to that of the methyl ester. The saponification of the two esters was tested, and in both cases, the reactions afforded the same spot observed by TLC and the same ^1^H NMR spectrum. The spectra were also identical to the spectrum of **9**, which confirmed its structure, that is a new 1*H*-azeto [1,2-*a*]quinoline derivative was isolated. This reaction also undoubtedly proves that **10a** and **10b** are acrylate derivatives with 3-(piperidin-1-ylmethyl) substitution.

#### 2.1.3. Reaction of 4-Hydroxyquinolines with Aromatic Aldehydes and Piperidine

**Scheme 4:**
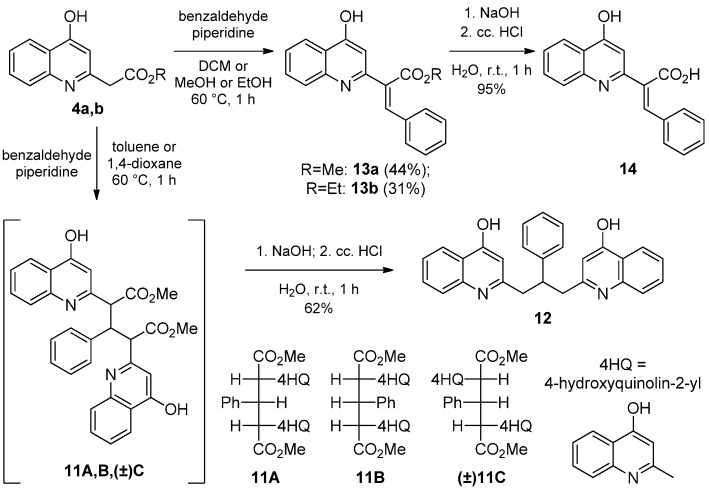
The reaction of 4-hydroxyquinolines with benzaldehyde in the presence of piperidine.

Our next goal was to change paraformaldehyde to aromatic aldehydes to explore the reactivity. First, benzaldehyde, piperidine, and **4a** were reacted (Scheme 4). Toluene and 1,4-dioxane were tested as solvents, but in both cases, the experiments led to the formation of bisquinolines (**11A**,**B**, (**±**)**C**). Synthetic verification was also desired performed by hydrolysis with NaOH, followed by neutralisation delivering **12** in good yields.

Thereafter, the solvent was changed to dichloromethane. The expected compound was a Mannich product. However, after isolation of the main product, its ^1^H NMR spectrum showed that the methylene group of the starting compound (**4a**) disappeared and the piperidine moiety was not incorporated. In the spectrum, the presence of 10 aromatic protons could be observed. According to these findings, the formation of methyl 4-hydroxyquinoline-3-phenyl acrylate (**13a**) was assumed, rather than that of the desired Mannich product (Scheme 4). The process can be explained by the Knoevenagel condensation [28], a reaction between the activated methylene group and benzaldehyde. A solvent change was carried out, testing MeOH instead of DCM, and a higher yield was observed. **13b** was furnished in a similar manner, starting from **4b** and applying EtOH as a solvent.

Furthermore, decarboxylation experienced previously was also tested, hypothesising that a styrilquinoline derivative would form. Styrilquinolines possess a rather wide range of biological activities [29], including antiproliferative [30,31,32,33], antiviral, and antibacterial [29] activities. Therefore, **13a** was stirred with NaOH in water at room temperature, then it was neutralised with cc. HCl. According to the ^1^H NMR spectrum, there was no additional proton sign, which would have indicated decarboxylation. Mass spectrometry also supported that only ester hydrolysis took place. The reaction was repeated at a higher temperature but again, only the carboxylic acid derivative was isolated (**14**).

**Scheme 5:**
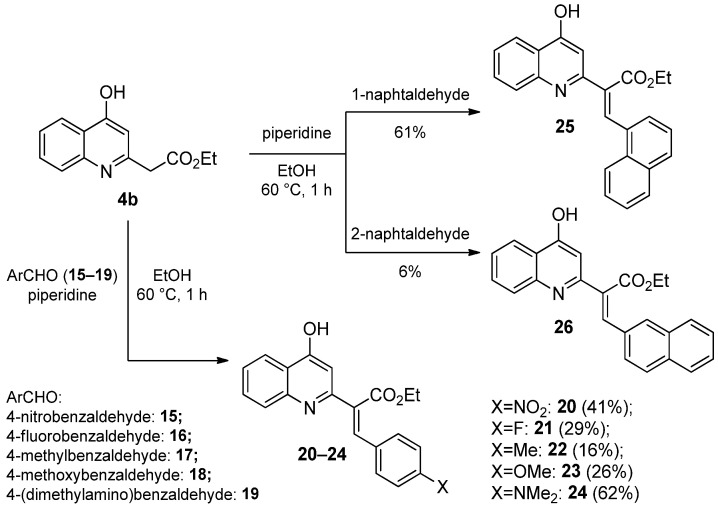
The reaction of 4-hydroxyquinolines with aromatic aldehydes in the presence of piperidine.

In order to investigate the extensibility of the reaction, the aldehyde component was modified. Since the yield of starting compound **4b** was higher than that of **4a**, we continued our work with only the ethyl ester (Scheme 5). Five *para*-substituted benzaldehyde (**15**–**19**) were tested. 4HQ acetate **4b**, the corresponding benzaldehyde and piperidine were dissolved in EtOH and the mixture was treated at reflux temperature for 1–10 h. The solubility of products in EtOH was lower than that of the starting compounds and consequently, the formed substances were crystallised from EtOH. In all cases, NMR confirmed the structure of the new *para*-substituted benzylidene derivatives (**20**–**24**). 1- and 2-naphthaldehyde were also tested with **25** isolated in a good yield, but **26** was formed in a much lower yield. A possible explanation is that 1-naphthyl promotes condensation, whereas the 2-naphthyl ring sterically repulses it.

**Scheme 6:**
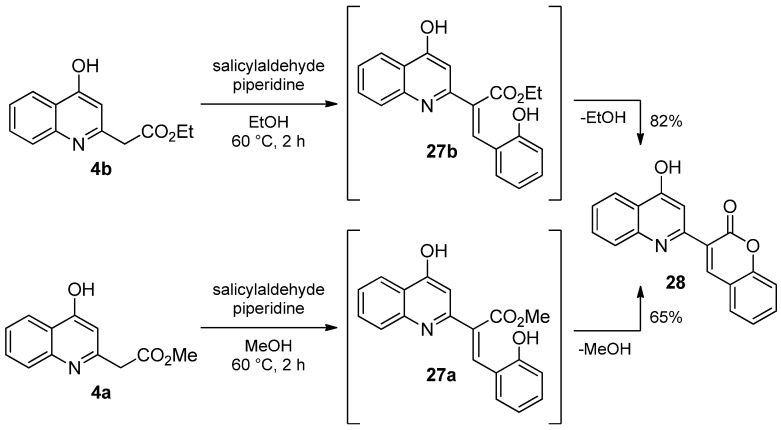
The reaction of 4-hydroxyquinolines with salicylaldehyde in the presence of piperidine.

In further investigation, an *ortho*-substituted benzaldehyde, namely salicylaldehyde, was tested. It was treated at reflux temperature with **4b** in presence of piperidine in EtOH, which led to the formation of a single product after a short reaction of 2 h. The ^1^H NMR spectrum of the isolated solid showed the absence of the ethyl ester group. It clearly indicates that compound **27b** is an intermediate product (Scheme 6), and an intramolecular ring closure takes place via ethanol loss; thus, a new 2*H*-chromen-2-one derivative (**28**) was isolated. Our hypothesis, i.e., spontaneous ring closure via alcohol loss, was confirmed by repeating the reaction from **4a**. As expected, the synthesis led to the formation of the same chromanone derivative (**28**).

The *Z*/*E* isomerism of benzylidene derivatives (**13ab**, **14**, **20**–**26**) synthesised previously was not described. Nevertheless, the ring closure of **28** can only take place if the intermediate products (**27a,b**) are *Z*-isomers, because the functional groups, which are involved in ring closure, are located in a sterically appropriate arrangement. This indirectly proves that all isolated compounds are *Z*-isomers.

**Scheme 7:**
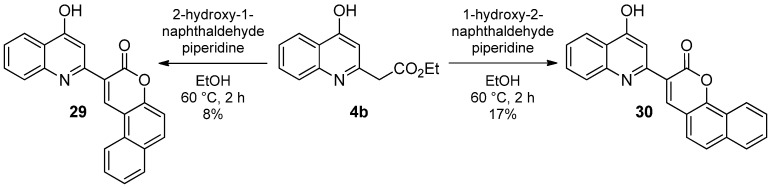
The reaction of 4-hydroxyquinolines with hydroxynaphthaldehydes in the presence of piperidine.

There are known synthesis pathways for 2-quinolyl coumarins [34,35,36,37], but our reaction (Scheme 5) provides a more convenient alternative synthetic route. The reaction was further tested for functionalised aromatic aldehydes such as 2-hydroxy-1-naphthaldehyde and 1-hydroxy-2-naphthaldehyde. In these cases, the expected intramolecular ring closure took place and compounds **29** and **30** were isolated (Scheme 7). The ring closure can only happen when the intermediates are *Z* isomers, which is a further support to our hypothesis about *Z*/*E* isomerism of the synthesised benzylidene derivatives.

### 2.2. Biological Evaluations

The cytotoxic activity of the derivatives was evaluated using doxorubicin-sensitive and -resistant colon adenocarcinoma cell lines (Colo 205 and Colo 320, respectively) and normal human embryonic MRC-5 fibroblasts (Table 2). The IC_50_ values below 20 µM were considered as cytotoxic. In this regard, the following compounds were cytotoxic on the resistant cancer cell line Colo 320 (IC_50_ values are expressed in µM): **20** (IC_50_: 4.61), **13b** (IC_50_: 4.58), **13a** (IC_50_: 8.19), **29** (IC_50_: 9.86), **26** (IC_50_: 11), **22** (IC_50_: 12.29), **28** (IC_50_: 14.08). Compounds **20** (IC_50_: 2.34), **13b** (IC_50_: 8.1), **22** (IC_50_: 11.79), **13a** (IC_50_: 11.86), **26** (IC_50_: 12.63), **21** (IC_50_: 16.54) exerted cytotoxic activity on the chemosensitive tumour cell line Colo 205.

The selectivity of the compounds towards cancer cells compared to normal cells was calculated using the non-tumoural MRC-5 human embryonic lung fibroblast cell line, as reported previously [38]. The different selectivity indexes (SI) were evaluated as the quotient of the IC_50_ value in the non-tumoural cells divided by the IC_50_ in the cancer cell line. The compounds’ activity towards cancer cells is considered to be strongly selective if the selectivity index (SI) value is higher than 6, moderately selective if 3 < SI < 6, slightly selective if 1 < SI < 3 and non-selective if SI is lower than 1. The most selective derivative towards the resistant Colo 320 cell line was **29** showing an SI higher than 6 (Table 3). Moderate selectivity towards MDR cancer cells was obtained in the presence of compounds **13b** and **13a**, with the selectivity indices 4.14 and 4.23, respectively. In addition, moderate selectivity was observed towards the sensitive cancer cell line Colo 205 with compound **20** (SI: 4.23).

Overseeing the results, mainly *para*-substituted benzylidene derivatives proved to be active compounds as cytotoxic agents. It was realised that the activity might correlate with some kind of effect of the functional groups. A well-known descriptor is the Hammett–Brown substituent *σ*^+^ (*σ_p_*^+^ for *para*-substituted derivatives), which sums up the electronic effect of a substituted group on a benzene ring [39]. Table 4 includes the constant *σ_p_*^+^ and the pIC_50_ obtained from IC_50_.

**Figure 1:**
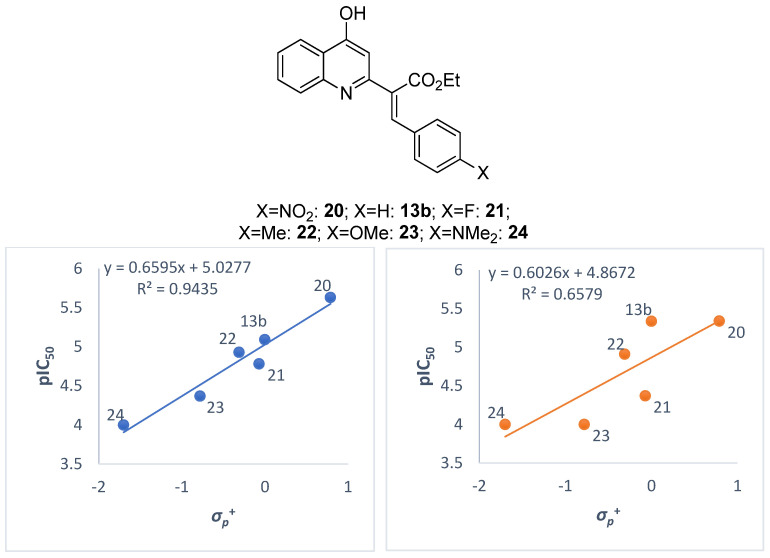
A correlation of the Hammet–Brown constant (*σ_p_*^+^) and pIC_50_ for compounds **13b**, **20**–**24**; regression lines and regression coefficients are given.

The Hammett–Brown substituent was correlated to the pIC_50_ values. The correlations are depicted in Figure 1. In the case of Colo 205, the R^2^ is 0.94, while Colo 320 showed a lower R^2^ (0.66).

## 3. Materials and Methods

### 3.1. Preparation Protocols for the Synthesis of the New Derivatives

Melting points were determined on a Hinotek X-4 melting point apparatus. Merck Kieselgel 60F_254_ plates were applied for TLC. Microwave reactions were carried out with a CEM Discover SP microwave reactor.

^1^H and ^13^C NMR spectra were recorded in DMSO-d_6_ solutions in 5 mm tubes at room temperature (RT), on a Bruker DRX-500 spectrometer (Bruker Biospin, Karlsruhe, Baden Wurttemberg, Germany) at 500 (^1^H) and 125 (^13^C) MHz, with the deuterium signal of the solvent as the lock and TMS as internal standard (^1^H, ^13^C). All spectra (^1^H, ^13^C) were acquired and processed with the standard BRUKER software.

The HRMS flow injection analysis was performed with Thermo Scientific Q Exactive Plus hybrid quadrupole-Orbitrap (Thermo Fisher Scientific, Waltham, MA, USA) mass spectrometer coupled to a Waters Acquity I-Class UPLC™ (Waters, Manchester, UK).

The intermediate enamines dimethyl and diethyl 3-(phenylimino)pentanedioate (**3a** and **3b**) were synthesised according to literature methods [18,19,23,40,41,42,43].

#### 3.1.1. General Procedure for the Synthesis of **4a,b** and **5**

Dimethyl or diethyl 3-(phenylimino)pentanedioate (2.71 mmol; 0.75 g **3a** or 0.674 g **3b**) dissolved in 1,2-dichlorobenzene (15 mL) was placed into a 35-mL pressurised reaction vial. The reaction mixture was heated in a CEM SP microwave reactor according to conditions given in Table 1 and then cooled down in iced water. The formed crystals were filtered out and washed with EtOAc/Et_2_O (30 mL).

Methyl 2-(4-hydroxyquinolin-2-yl) acetate (**4a**)

Pale beige solid; yield: 31% (203 mg); m.p.: 211–214 °C (lit [21]: 179 °C); ^1^H NMR ([D_6_] DMSO): δ = 3.68 (3H, s); 3.79 (2H, s); 6.01 (1H, s); 7.30 (1H, t, *J* = 7.5 Hz); 7.51 (1H, d, *J* = 7.7 Hz); 7.64 (1H, t, *J* = 8.1 Hz); 8.05 (1H, d, *J* = 7.8 Hz); 11.69 (1H, s); ^13^C NMR ([D_6_]DMSO): δ = 39.0; 52.7; 110.3; 118.5; 123.5; 125.1; 125.3; 132.2; 140.6; 146.1; 169.8; 177.4. HRMS calcd for [M + H^+^] *m*/*z* = 218.0812, found *m*/*z* = 218.0808 (Figures S1–S3).

Ethyl 2-(4-hydroxyquinolin-2-yl) acetate (**4b**)

Pale beige solid; yield: 41% (256 mg); m.p.: 208–210 °C (lit [18]: 202–204 °C); ^1^H NMR ([D_6_] DMSO): δ = 1.22 (3H, t, *J* = 7.0 Hz); 3.76 (2H, s); 4.14 (2H, q, *J* = 7.2 Hz); 6.00 (1H, s); 7.30 (1H, t, *J* = 7.4 Hz); 7.51 (1H, d, *J* = 8.2 Hz); 7.63 (1H, t, *J* = 7.5 Hz); 8.05 (1H, d, *J* = 8.1 Hz); 11.68 (1H, s); ^13^C NMR ([D_6_]DMSO): δ = 14.5; 39.2; 61.4; 110.3; 118.5; 123.5; 125.1; 125.3; 132.2; 140.6; 146.2; 169.3; 177.4. HRMS calcd for [M + H^+^] *m*/*z* = 232.0968, found *m*/*z* = 232.0964 (Figures S4–S6).

1-Phenyl-4-(phenylamino)pyridine-2,6(1*H*,3*H*)-dione (**5**)

Pale beige solid; yield: 53% (444 mg); m.p.: 294–297 °C (lit [22]: 281–283); ^1^H NMR ([D_6_] DMSO): δ = 5.41 (1H, s); 7.13 (2H, d, *J* = 7.2 Hz); 7.19 (1H, t, *J* = 7.4 Hz); 7.26 (2H, d, *J* = 7.7 Hz); 7.33–7.46 (5H, m); 9.07 (1H, s); ^13^C NMR ([D_6_]DMSO): δ = 37.0; 87.6; 123.0; 125.1; 128.0; 129.0; 129.6; 129.9; 136.4; 139.3; 153.5; 166.8; 168.9. HRMS calcd for [M + H^+^] *m*/*z* = 279.1128, found *m*/*z* = 279.1126 (Figures S7–S9).

#### 3.1.2. General Procedure for the Synthesis of Bisquinoline Derivatives (**7**, **12**)

4-Hydroxyquinoline derivative **4a** (0.43 mmol, 94 mg), aldehyde (0.43 mmol; 13 mg paraformaldehyde or 46 mg benzaldehyde) and piperidine (0.43 mmol, 37 mg) were placed in a 50-mL round bottom flask, and dissolved in 25 mL of toluene. It was heated at 60 °C for 1 h, then evaporated. Then NaOH (0.86 mmol, 34 mg) and 20 mL distilled water were added to the residue and stirred for 1 h at room temperature. 4 drops of cc. HCl were added, when white crystals precipitated and were filtered out.

2,2′-(Propane-1,3-diyl)bis(quinolin-4-ol) (**7**)

White solid; yield: 69% (98 mg); m.p.: >350 °C; ^1^H NMR ([D_6_] DMSO): δ = 2.12 (2H, quint, *J* = 7.6 Hz); 2.70 (4H, t, *J* = 7.6 Hz); 6.01 (2H, s); 7.29 (2H, t, *J* = 7.5 Hz); 7.54 (2H, d, *J* = 8.4 Hz); 7.62 (2H, t, *J* = 7.6 Hz); 8.05 (2H, d, *J* = 7.9); 11.63 (2H, s); ^13^C NMR ([D_6_]DMSO): δ = 27.5; 33.0; 108.2; 118.5; 123.4; 125.0; 125.2; 132.0; 140.6; 153.3; 177.1. HRMS calcd for [M + H^+^] *m*/*z* = 331.1441, found *m*/*z* = 331.1441 (Figures S10–S12).

2,2′-(2-Phenylpropane-1,3-diyl)bis(quinolin-4-ol) (**12**)

White solid, yield: 62% (109 mg); m.p.: 199–202 °C; ^1^H NMR ([D_6_] DMSO): δ = 3.08–3.15 (2H, m); 3.17–3.22 (2H, m); 3.90 (1H, quint, *J* = 7.6 Hz); 6.12 (2H, s); 7.11 (1H, t, *J* = 7.2 Hz); 7.21 (1H, t, *J* = 7.5 Hz); 7.30 (2H, d, *J* = 7.6 Hz); 7.34 (2H, t, *J* = 7.5 Hz); 7.67 (2H, t, *J* = 7.7 Hz); 7.71 (2H, d, *J* = 8.2 Hz); 8.01 (2H, d, *J* = 8.0 Hz); 12.54 (2H, s); ^13^C NMR ([D_6_]DMSO): δ = 44.1; 108.8; 118.8; 123.8; 124.2; 124.8; 127.2; 128.0; 128.8; 132.4; 140.4; 142.3; 153.0; 175.3. HRMS calcd for [M + H^+^] *m*/*z* = 407.1754, found *m*/*z* = 407.1756 (Figures S13–S15).

#### 3.1.3. Synthesis of 2-(4-Hydroxyquinolin-2-yl) Acetic Acid (**8**)

4-Hydroxyquinoline derivative **4a** (0.46 mmol, 100 mg), Na_2_CO_3_ (0.94 mmol, 100 mg), and 20 mL distilled water were placed into a 50-mL round bottom flask. It was stirred for 12 h at room temperature. Then 3 drops of cc. HCl were added, and the precipitation of white crystals was observed, which were filtered out. Yield: 91% (85 mg); mp.: 221–224 °C; ^1^H NMR ([D_6_] DMSO): δ = 3.26 (2H, s); 5.81 (1H, s); 7.23 (1H, d, *J* = 7.1 Hz); 7.52–7.59 (2H, m); 8.03 (1H, d, *J* = 7.9 Hz); ^13^C NMR ([D_6_]DMSO): δ = 42.7; 108.5; 118.7; 122.8; 125.2; 125.2; 131.5; 140.3; 151.8; 171.4; 177.1. HRMS calcd for [M + H^+^] *m*/*z* = 204.0655, found *m*/*z* = 204.0659 (Figures S16–S18).

#### 3.1.4. Synthesis of 3-(Piperidin-1-Ylmethyl)-1H-Azeto [1,2-a] Quinolin-4(2H)-One (**9**)

Procedure A: 100 mg Acetic acid derivative **8** yielded via Na_2_CO_3_ hydrolysis (0.49 mmol) and 20 mL 1,4-dioxane were placed into a 50-mL round bottom flask and stirred at 100 °C. Then 3 drops of cc. HCl were added, paraformaldehyde (1.00 mmol, 30 mg), and piperidine (1.00 mmol, 85 mg) were added. The mixture was stirred for 1 h at 100 °C when the product was precipitated. It was purified by neutral column chromatography (DCM:MeOH, 4:1). Yield: 80% (105 mg).

Procedure B: 30 mg of **10b** (0.10 mmol), 30 mg of NaOH (0.75 mmol), and 5 mL distilled water were placed into a 25-mL round bottom flask. The mixture was stirred for 2 h at room temperature and then neutralised with cc. HCl. The mixture was evaporated, and the residue was crystallised from Et_2_O (5 mL), and recrystallised from MeOH/Et_2_O. Yield: 43% (11 mg).

White solid; mp.: >350 °C; ^1^H NMR ([D_6_] DMSO): δ = 1.57–1.70 (2H, m); 1.75–1.84 (2H, m); 1.85–1.94 (2H, m); 3.21 (2H, t, *J* = 6.7 Hz); 3.49–3.55 (4H, m); 3.85 (2H, t, *J* = 6.8 Hz); 4.43 (2H, s); 7.31–7.36 (1H, m); 7.67–7.70 (2H, m); 8.10 (1H, d, *J* = 8.3 Hz); 12.65 (1H, s); ^13^C NMR ([D_6_]DMSO): δ = 19.7; 21.6; 22.2; 52.9; 56.5; 59.1; 106.4; 118.5; 123.7; 123.7; 125.1; 132.5; 140.0; 143.3; 174.8. HRMS calcd for [M + H^+^] *m*/*z* = 269.1648, found *m*/*z* = 269.1651 (Figures S19–S21).

#### 3.1.5. General Procedure for the Synthesis of Mannich Bases (**10a,b**)

4-Hydroxyquinoline derivative (0.43 mmol; 94 mg **4a** or 100 mg **4b**), paraformaldehyde (2.16 mmol, 65 mg), and piperidine (2.16 mmol, 184 mg) were placed in a 50-mL round bottom flask, and dissolved in 30 mL of DCM. After 75 min reflux, the mixture was evaporated and then purified by column chromatography.

Methyl 2-(4-hydroxy-3-(piperidin-1-ylmethyl)quinolin-2-yl) acetate (**10a**)

The residue was purified by column chromatography (DCM:MeOH, 9:1), and was crystallised from Et_2_O (10 mL). It was a pale beige solid; yield: 24% (32 mg); m.p.: 166–169 °C; ^1^H NMR ([D_6_] DMSO): δ = 1.57–1.67 (2H, m); 1.69–1.79 (4H, m); 3.25–3.29 (4H, m); 3.56 (3H, s); 4.20 (2H, s); 4.33 (2H, s); 7.05 (1H, t, *J* = 7.5 Hz); 7.23 (1H, d, *J* = 7.7 Hz); 7.38 (1H, t, *J* = 7.7 Hz); 7.89 (1H, d, *J* = 7.7 Hz); 12.13 (1H, s); ^13^C NMR ([D_6_]DMSO): δ = 20.1; 21.8; 49.8; 57.5; 57.7; 58.1; 63.1; 63.5; 92.8; 116.4; 121.0; 123.4; 124.7; 130.4; 139.1; 148.9; 166.8; 170.3. HRMS calcd for [M + H^+^] *m*/*z* = 327.1703, found *m*/*z* = 327.1702 (Figures S22–S24).

Ethyl 2-(4-hydroxy-3-(piperidin-1-ylmethyl)quinolin-2-yl) acetate (**10b**)

The residue was purified by column chromatography (DCM:EtOH, 4:1), and was crystallised from Et_2_O (12 mL). It was a pale beige solid; yield: 75% (53 mg); m.p.: 152–155 °C; ^1^H NMR ([D_6_] DMSO): δ = 1.21 (3H, t, *J* = 7.1 Hz); 1.58–1.68 (2H, m); 1.69–1.80 (4H, m); 3.26–3.30 (4H, m); 4.06 (2H, q, *J* = 6.9 Hz); 4.20 (2H, s); 4.33 (2H, s); 7.05 (1H, t, *J* = 7.3 Hz); 7.23 (1H, d, *J* = 8.1 Hz); 7.39 (1H, t, *J* = 7.9 Hz); 7.90 (1H, d, *J* = 7.9 Hz); 12.17 (1H, s); ^13^C NMR ([D_6_]DMSO): δ = 15.6; 20.2; 21.8; 57.5; 57.6; 57.7; 58.0; 58.2; 63.2; 63.5; 92.7; 116.4; 121.0; 123.4; 124.7; 130.4; 139.1; 149.0; 166.6; 170.3. HRMS calcd for [M + H^+^] *m*/*z* = 341.1860, found *m*/*z* = 341.1854 (Figures S25–S27).

#### 3.1.6. General Procedure for the Synthesis of the Knoevenagel Derivatives **13a,b**; **20**–**26**; **28**–**30**

4-Hydroxyquinoline derivative (0.43 mmol; 94 mg **4a** or 100 mg **4b**), aromatic aldehyde (0.43 mmol; 55 mg benzaldehyde or 78 mg 4-nitrobenzaldehyde or 64 mg 4-fluorobenzaldehyde or 62 mg 4-methylbenzaldehyde or 71 mg 4-methoxybenzaldehyde or 77 mg 4-(dimethylamino)benzaldehyde or 81 mg 1-naphthaldehyde or 81 mg 2-naphthaldehyde or 63 mg salicylaldehyde or 89 mg 2-hydroxy-1-naphthaldehyde or 89 mg 1-hydroxy-2-naphthaldehyde), and piperidine (0.21 mmol, 18 mg) were placed in a 50-mL round bottom flask, and dissolved in 25 mL of MeOH or EtOH, respectively. The mixture was heated by an oil bath at reflux temperature or stirred at room temperature for 1–10 h. After evaporation, the products were purified by column chromatography and then crystallised.

(*Z*)-methyl 2-(4-hydroxyquinolin-2-yl)-3-phenylacrylate (**13a**)

The reaction mixture was treated under reflux conditions for 6 h. The mixture was evaporated, then purified by column chromatography (DCM:MeOH, 9:1). The desired product was isolated by crystallisation from DCM (10 mL); yellow solid; yield: 44% (58 mg); m.p.: 247–251 °C; ^1^H NMR ([D_6_] DMSO): δ = 3.77 (3H, s); 5.92 (1H, s); 7.29–7.40 (6H, m); 7.53 (1H, d, *J* = 7.6 Hz); 7.66 (1H, t, *J* = 7.3 Hz); 7.99 (1H, s); 8.11 (1H, d, *J* = 7.8 Hz); 11.92 (1H, s); ^13^C NMR ([D_6_]DMSO): δ = 53.1; 110.3; 118.9; 123.9; 125.4; 125.6; 126.4; 129.3; 130.6; 131.0; 132.4; 133.4; 140.9; 143.9; 146.3; 166.0; 177.4. HRMS calcd for [M + H^+^] *m*/*z* = 306.1125, found *m*/*z* = 306.1119 (Figures S28–S30).

(*Z*)-ethyl 2-(4-hydroxyquinolin-2-yl)-3-phenylacrylate (**13b**)

The reaction was treated at reflux temperature for 10 h. The eluent was evaporated, and the residue was purified by column chromatography (CHCl_3_:EtOH, 19:1). The product was isolated by crystallisation from ET_2_O (8 mL); yellow solid; yield: 31% (43 mg); m.p.: 222–225 °C; ^1^H NMR ([D_6_] DMSO): δ = 1.23 (3H, t, *J* = 7.1 Hz); 4.25 (2H, q, *J* = 7.2 Hz); 5.91 (1H, s); 7.28–7.38 (6H, m); 7.53 (1H, d, *J* = 8.3 Hz); 7.67 (1H, t, *J* = 7.5 Hz); 7.99 (1H, s); 8.10 (1H, d, *J* = 8.2 Hz); 11.97 (1H, s); ^13^C NMR ([D_6_]DMSO): δ = 14.6; 61.8; 110.2; 118.9; 123.9; 125.4; 125.5; 126.6; 129.3; 130.6; 131.0; 132.4; 133.4; 140.8; 143.7; 146.4; 165.5; 177.3. HRMS calcd for [M + H^+^] *m*/*z* = 320.1281, found *m*/*z* = 320.1277 (Figures S31–S33).

(*Z*)-ethyl 2-(4-hydroxyquinolin-2-yl)-3-(4-nitrophenyl)acrylate (**20**)

Reflux time: 4 h. The mixture was evaporated, and the purification was carried out by column chromatography (DCM:*n*-hexane:EtOH, 7:2.5:0.5). The final product was isolated by crystallisation from DCM/EtOAc (10 mL); yellow solid; yield: 41% (64 mg); m.p.: 257–260 °C; ^1^H NMR ([D_6_] DMSO): δ = 1.24 (3H, t, *J* = 7.3 Hz); 4.28 (2H, q, *J* = 7.2 Hz); 5.89 (1H, s); 7.35 (1H, t, *J* = 7.4 Hz); 7.52 (1H, d, *J* = 8.4 Hz); 7.60 (2H, d, *J* = 8.8 Hz); 7.67 (1H, t, *J* = 7.6 Hz); 8.08 (1H, d, *J* = 7.8 Hz); 8.10 (1H, s); 8.15 (2H, d, *J* = 7.2 Hz); 11.96 (1H, s); ^13^C NMR ([D_6_]DMSO): δ = 14.5; 62.1; 110.5; 118.9; 123.9; 124.2; 125.4; 125.6; 130.1; 131.4; 132.5; 140.1; 140.8; 141.5; 145.4; 148.3; 165.0; 177.3. HRMS calcd for [M + H^+^] *m*/*z* = 365.1132, found *m*/*z* = 365.1129 (Figures S34–S36).

(*Z*)-ethyl 3-(4-fluorophenyl)-2-(4-hydroxyquinolin-2-yl)acrylate (**21**)

The mixture was stirred under reflux conditions for 6 h, then evaporated, and purified by column chromatography (DCM:EtOH, 19:1). The isolation was carried out by crystallisation from DCM/EtOAc (9 mL); yellow solid; yield: 29% (43 mg); m.p.: 233–236 °C; ^1^H NMR ([D_6_] DMSO): δ = 1.23 (3H, t, *J* = 7.2 Hz); 4.25 (2H, q, *J* = 7.1 Hz); 5.91 (1H, s); 7.17 (2H, t, *J* = 7.9 Hz); 7.35 (1H, t, *J* = 7.5 Hz); 7.39–7.44 (2H, m); 7.53 (1H, d, *J* = 8.4 Hz); 7.66 (1H, t, *J* = 7.8 Hz); 7.98 (1H, s); 8.11 (1H, d, *J* = 8.1 Hz); 11.90 (1H, s); ^13^C NMR ([D_6_]DMSO): δ = 14.6; 61.8; 110.2; 116.5 (*^2^J_C-F_* = 21.8 Hz); 118.9; 123.9; 125.4; 125.6; 126.4; 130.1; 132.4; 133.1 (*^3^J_C-F_* = 9.2 Hz); 140.9; 142.5; 146.2; 162.5; 165.0 (*^1^J_C-F_* = 118.4 Hz); 177.4. HRMS calcd for [M + H^+^] *m*/*z* = 338.1187, found *m*/*z* = 338.1182 (Figures S37–S39).

(*Z*)-ethyl 2-(4-hydroxyquinolin-2-yl)-3-(*p*-tolyl)acrylate (**22**)

Reflux time: 8 h. The mixture was evaporated, and the residue was purified by column chromatography (DCM:EtOH, 9:1). The desired product was crystallised from DCM/EtOAc (12 mL); yellow solid; yield: 16% (23 mg); m.p.: 224–227 °C; ^1^H NMR ([D_6_] DMSO): δ = 1.22 (3H, t, *J* = 6.9 Hz); 2.24 (3H, s); 4.24 (2H, q, *J* = 6.8 Hz); 5.90 (1H, s); 7.13 (2H, d, *J* = 7.5 Hz); 7.24 (2H, d, *J* = 7.5 Hz); 7.36 (1H, t, *J* = 7.4 Hz); 7.53 (1H, d, *J* = 8.3 Hz); 7.67 (1H, t, *J* = 7.8 Hz); 7.94 (1H, s); 8.11 (1H, d, *J* = 7.9 Hz); 11.96 (1H, s); ^13^C NMR ([D_6_]DMSO): δ = 14.6; 21.4; 61.7; 110.2; 118.8; 123.9; 125.4; 125.5; 125.6; 130.0; 130.6; 130.7; 132.4; 140.9; 141.3; 143.7; 146.6; 165.6; 177.4. HRMS calcd for [M + H^+^] *m*/*z* = 334.1438, found *m*/*z* = 334.1434 (Figures S40–S42).

(*Z*)-ethyl 2-(4-hydroxyquinolin-2-yl)-3-(4-methoxyphenyl)acrylate (**23**)

The reaction was treated at reflux temperature for 9 h, then it was evaporated. The purification was carried out by column chromatography (DCM:EtOH, 19:1). The desired product was isolated by crystallisation from EtOH (5 mL). It was a yellow solid; yield: 26% (39 mg); m.p.: 215–218 °C; ^1^H NMR ([D_6_] DMSO): δ = 1.22 (3H, t, *J* = 7.2 Hz); 3.72 (3H, s); 4.23 (2H, q, *J* = 7.0 Hz); 5.92 (1H, s); 6.89 (2H, d, *J* = 7.4 Hz); 7.31 (2H, d, *J* = 8.7 Hz); 7.36 (1H, t, *J* = 7.5 Hz); 7.53 (1H, d, *J* = 7.3 Hz); 7.66 (1H, t, *J* = 7.2 Hz); 7.92 (1H, s); 8.11 (1H, d, *J* = 7.4 Hz); 11.92 (1H, s); ^13^C NMR ([D_6_]DMSO): δ = 14.6; 55.8; 61.5; 110.1; 115.0; 118.9; 123.7; 123.9; 125.4; 125.5; 125.7; 132.4; 132.8; 140.9; 143.4; 146.9; 161.7; 165.7; and 177.4. HRMS calcd for [M + H^+^] *m*/*z* = 350.1387, found *m*/*z* = 350.1384 (Figures S43–S45).

(*Z*)-ethyl 3-(4-(dimethylamino)phenyl)-2-(4-hydroxyquinolin-2-yl)acrylate (**24**)

The reaction was stirred under reflux conditions for 3 h, then it was evaporated. The residue was purified by column chromatography (DCM:EtOH, 4:1). The crystallisation was carried out from DCM/EtOAc (19 mL); pale brown solid; yield: 62% (98 mg); m.p.: 256–259 °C; ^1^H NMR ([D_6_] DMSO): δ = 1.20 (3H, t, *J* = 7.1 Hz); 2.90 (6H, s); 4.20 (2H, q, *J* = 7.3 Hz); 5.92 (1H, s); 6.58 (2H, d, *J* = 9.1 Hz); 7.16 (2H, d, *J* = 9.1 Hz); 7.35 (1H, t, *J* = 7.5); 7.54 (1H, d, *J* = 8.4 Hz); 7.65 (1H, t, *J* = 7.7 Hz); 7.81 (1H, s); 8.12 (1H, d, *J* = 7.7 Hz); 11.83 (1H, s); ^13^C NMR ([D_6_]DMSO): δ = 14.7; 61.1; 110.3; 112.1; 118.8; 119.8; 120.2; 123.7; 125.4; 125.6; 132.2; 132.9; 141.1; 144.1; 147.7; 152.2; 166.1; 177.5.HRMS calcd for [M + H^+^] *m*/*z* = 363.1703, found *m*/*z* = 363.1700 (Figures S46–S48).

(*Z*)-ethyl 2-(4-hydroxyquinolin-2-yl)-3-(naphthalen-1-yl)acrylate (**25**)

Reflux time: 3 h. The mixture was evaporated, then purified by column chromatography (DCM:EtOH, 9:1). The final product was isolated by crystallisation from EtOH (12 mL); yellow solid; yield: 61% (97 mg); m.p.: 262–265 °C; ^1^H NMR ([D_6_] DMSO): δ = 1.29 (3H, t, *J* = 7.0 Hz); 4.33 (2H, q, *J* = 7.3 Hz); 5.68 (1H, s); 7.26–7.36 (3H, m); 7.52 (1H, d, *J* = 8.2 Hz); 7.58–7.68 (3H, m); 7.90 (1H, d, *J* = 7.5 Hz); 7.94–8.00 (2H, m); 8.09 (1H, d, *J* = 8.2 Hz); 8.61 (1H, s); 11.95 (1H, s); ^13^C NMR ([D_6_]DMSO): δ = 14.6; 61.9; 110.6; 118.7; 123.7; 124.4; 125.3; 125.3; 125.8; 127.0; 127.0; 127.6; 129.1; 130.0; 130.5; 131.3; 131.5; 132.3; 133.5; 140.6; 142.5; 146.1; 165.2; 177.1. HRMS calcd for [M + H^+^] *m*/*z* = 370.1438, found *m*/*z* = 370.1435 (Figures S49–S51).

(*Z*)-ethyl 2-(4-hydroxyquinolin-2-yl)-3-(naphthalen-2-yl)acrylate (**26**)

The mixture was stirred at room temperature for 2 h. The eluent was evaporated, and the residue was purified by column chromatography (CHCl_3_:EtOH, 19:1). The desired product was crystallised from Et_2_O (8 mL); brick-red solid; yield: 6% (9 mg); 177–180 °C; ^1^H NMR ([D_6_] DMSO): δ = 1.26 (3H, t, *J* = 6.9 Hz); 4.28 (2H, q, *J* = 6.8 Hz); 5.95 (1H, s); 7.25 (1H, d, *J* = 8.9 Hz); 7.37 (1H, t, *J* = 7.8 Hz); 7.50–7.58 (3H, m); 7.67 (1H, t, *J* = 7.8 Hz); 7.75 (1H, d, *J* = 8.62 Hz); 7.77–7.84 (2H, m); 8.06–8.15 (3H, m); 11.97 (1H, s); ^13^C NMR ([D_6_]DMSO): δ = 14.6; 61.8; 110.5; 118.9; 123.9; 125.5; 125.6; 125.6; 126.8; 127.4; 128.0; 128.4; 128.7; 129.0; 131.1; 132.4; 132.8; 133.1; 133.9; 140.9; 143.8; 146.4; 165.6; 177.3. HRMS calcd for [M + H^+^] *m*/*z* = 370.1438, found *m*/*z* = 370.1434 (Figures S52–S54).

3-(4-Hydroxyquinolin-2-yl)-*2H*-chromen-2-one (**28**)

Starting compound: **4a**; reflux time: 2 h. The purification of the evaporated mixture was carried out by column chromatography (DCM:MeOH, 19:1). The final product was isolated by crystallisation from EtOH (18 mL); yellow solid; yield: 82% (103 mg); m.p.: 315–319 °C; ^1^H NMR ([D_6_] DMSO): δ = 6.40 (1H, s); 7.36 (1H, t, *J* = 7.2 Hz); 7.46 (1H, t, *J* = 7.6 Hz); 7.53 (1H, d, *J* = 7.3 Hz); 7.63–7.72 (2H, m); 7.75 (1H, t, *J* = 7.3 Hz); 7.87 (1H, d, *J* = 7.5 Hz); 8.11 (1H, d, *J* = 7.8 Hz); 8.58 (1H, s); 11.78 (1H, s); ^13^C NMR ([D_6_]DMSO): δ = 110.0; 116.7; 119.0; 119.0; 122.0; 123.9; 125.3; 125.4; 125.6; 130.0; 132.6; 133.8; 140.6; 144.7; 145.2; 154.0; 158.9; 177.4. HRMS calcd for [M + H^+^] *m*/*z* = 290.0812, found *m*/*z* = 290.0808 (Figures S55–S57).

2-(4-Hydroxyquinolin-2-yl)-*3H*-benzo[*f*]chromen-3-one (**29**)

Starting compound: **4a**. The mixture was stirred at room temperature for 3 h. It was evaporated, then the residue was purified by column chromatography (DCM:*n*-hexane:MeOH, 7:2.5:0.5). The isolation was carried out by crystallisation from Et_2_O (8 mL); yellow solid; yield: 8% (12 mg); m.p.: 295–298 °C; ^1^H NMR ([D_6_] DMSO): δ = 6.58 (s, 1H); 7.37 (1H, s, brs); 7.65–7.72 (4H, m); 7.80 (1H, t, *J* = 7.2 Hz); 8.13 (2H, t, *J* = 7.5 Hz); 8.33 (1H, d, *J* = 7.4 Hz); 8.77 (1H, d, *J* = 7.2 Hz); 9.37 (1H, s); 11,84 (1H, s); ^13^C NMR ([D_6_]DMSO): δ = 110.4; 113.4; 117.0; 119.1; 120.9; 123.4; 123.8; 125.3; 125.4; 127.0; 129.2; 129.5; 129.5; 130.5; 132.5; 135.3; 140.7; 140.8; 145.1; 154.1; 159.0; 177.4. HRMS calcd for [M + H^+^] *m*/*z* = 340.0968, found *m*/*z* = 340.0966 (Figures S58–S60).

3-(4-Hydroxyquinolin-2-yl)-*2H*-benzo[*h*]chromen-2-one (**30**)

Starting compound: **4a**; reflux time: 1 h. The mixture was evaporated, then the residue was purified by column chromatography (DCM:EtOH, 9:1). The final product was crystallised from EtOH (11 mL); yellow solid; yield: 17% (25 mg); m.p.: >350 °C; ^1^H NMR ([D_6_] DMSO): δ = 6.49 (1H, s); 7.34–7.39 (1H, m); 7.67–7.72 (2H, m); 7.77–7.82 (2H, m); 7.85 (1H, d, *J* = 8.5 Hz); 7.95 (1H, d, *J* = 8.5 Hz); 8.09–8.15 (2H, m); 8.44–8.48 (1H, m); 8.73 (1H, s); 11.79 (1H, s); ^13^C NMR ([D_6_]DMSO): δ = 109.9; 114.8; 119.0; 121.4; 122.1; 122.4; 123.3; 123.8; 125.1; 125.3; 125.5; 128.3; 128.8; 129.9; 132.5; 135.5; 140.6; 145.1; 145.3; 158.9; 177.4. HRMS calcd for [M + H^+^] *m*/*z* = 340.0968, found *m*/*z* = 340.0966 (Figures S61–S63).

#### 3.1.7. Synthesis of (Z)-2-(4-hydroxyquinolin-2-yl)-3-phenylacrylic Acid (**14**)

Phenylacrilate derivative **13a** (0.33 mmol, 100 mg) and NaOH (0.33 mmol, 13 mg) in distilled water (25 mL) were stirred at room temperature for 8 h. 2 drops of cc. HCl were added, when white crystals precipitated, which were filtered out. White solid; yield: 95% (91 mg); m.p.: 230–233 °C; ^1^H NMR ([D_6_] DMSO): δ = 5.88 (1H, s); 7.28–7.38 (6H, m); 7.55 (1H, d, *J* = 8.2 Hz); 7.66 (1H, t, *J* = 7.8 Hz); 7.94 (1H, s); 8.10 (1H, d, *J* = 8.0 Hz); 11.97 (1H, s); 13.19 (1H, brs); ^13^C NMR ([D_6_]DMSO): δ = 110.0; 118.8; 123.8; 125.4; 125.5; 127.5; 129.3; 130.5; 130.8; 132.3; 133.6; 140.9; 143.1; 147.1; 167.0; 177.3. HRMS calcd for [M + H^+^] *m*/*z* = 292.0968, found *m*/*z* = 292.0966 (Figures S64–S66).

Physical-chemical properties of the synthesised 4-hydroxyquinolines are listed in Table S1.

### 3.2. Biological Assays

#### 3.2.1. Cell Lines

Human colonic adenocarcinoma cell lines Colo 205 doxorubicin-sensitive (ATCC-CCL-222) and Colo 320/MDR-LRP multidrug resistant overexpressing ABCB1 (MDR1)-LRP (ATCC-CCL-220.1) were purchased from LGC Promochem, Teddington, UK. The cells were cultured in RPMI 1640 medium supplemented with 10% heat-inactivated fetal bovine serum, 2 mM L-glutamine, 1 mM Na-pyruvate and 100 mM Hepes. The cell lines were incubated at 37 °C, in a 5% CO_2_, 95% air atmosphere. The semi-adherent human colon cancer cells were detached with Trypsin-Versene (EDTA) solution for 5 min at 37 °C.

MRC-5 human embryonal lung fibroblast cell lines (ATCC CCL-171) were purchased from LGC Promochem, Teddington, UK. The cell line was cultured in Eagle’s Minimal Essential Medium (EMEM, containing 4.5g/L glucose) supplemented with a non-essential amino acid mixture, a selection of vitamins and 10% heat-inactivated fetal bovine serum. The cell lines were incubated at 37 °C, in a 5% CO_2_, 95% air atmosphere.

#### 3.2.2. Cytotoxicity Assay

In the study, MRC-5 non-cancerous human embryonic lung fibroblast and human colonic adenocarcinoma cell lines (doxorubicin-sensitive Colo 205 and multidrug resistant Colo 320 colonic adenocarcinoma cells) were used to determine further the effect of compounds on cell growth. The effects of increasing concentrations of compounds on cell growth were tested in 96-well flat-bottomed microtiter plates. The compounds were diluted in a volume of 100 μL of medium.

The adherent human embryonal lung fibroblast cells were cultured in 96-well flat-bottomed microtiter plates, using EMEM supplemented with 10% heat-inactivated fetal bovine serum. The density of the cells was adjusted to 1 × 10^4^ cells in 100 μL per well, the cells were seeded for 24 h at 37 °C, 5% CO_2,_ then the medium was removed from the plates containing the cells, and the dilutions of compounds previously made in a separate plate were added to the cells in 200 μL.

In regards to the colonic adenocarcinoma cells, the two-fold serial dilutions of compounds were prepared in 100 μL of RPMI 1640, horizontally. The semi-adherent colonic adenocarcinoma cells were treated with Trypsin-Versene (EDTA) solution. They were adjusted to a density of 1 × 10^4^ cells in 100 μL of RPMI 1640 medium, and were added to each well, with the exception of the medium control wells. The final volume of the wells containing compounds and cells was 200 μL.

The culture plates were incubated at 37 °C for 24 h; at the end of the incubation period, 20 μL of MTT (thiazolyl blue tetrazolium bromide, Merck KGaA, Darmstadt, Germany) solution (from a stock solution of 5 mg/mL) were added to each well. After incubation at 37 °C for 4 h, 100 μL of sodium dodecyl sulfate (SDS) (Merck KGaA, Darmstadt, Germany) solution (10% in 0.01 M HCI) were added to each well and the plates were further incubated at 37 °C overnight. Cell growth was determined by measuring the optical density (OD) at 540 nm (ref. 630 nm) with Multiscan EX ELISA reader (Thermo Labsystems, Cheshire, WA, USA). Inhibition of cell growth was expressed as IC_50_ values, defined as the inhibitory dose that reduces the growth of the cells exposed to the tested compounds by 50%. IC_50_ values and the SD of triplicate experiments were calculated by using GraphPad Prism software version 5.00 for Windows with nonlinear regression curve fit (GraphPad Software, San Diego, CA, USA; www.graphpad.com).

## 4. Conclusions

The Conrad–Limpach reaction for methyl and ethyl 2-(4-hydroxyquinolin-2-yl) acetates was optimised under microwave irradiation. The formation of the by-product 1-phenyl-4-(phenylamino)pyridine-2,6(1*H*,3*H*)-dione could be reduced, but it was also synthesised as the main product. Depending on the solvent and on the used equivalents of formaldehyde and piperidine, Mannich bases or bisquinolines were furnished. In the former case, 3-piperidine-1-yl-methyl acrylate derivatives of 4-hydroxyquinolines were isolated. In the latter case, bisquinolines were transformed to 2,2′-(propane-1,3-diyl)bis(quinolin-4-ol) via decarboxylation. The hydrolysis of 3-piperidine-1-yl-methyl acrylate led to a novel 1*H*-azeto [1,2-*a*]quinoline, which was also synthesised starting from 2-(4-hydroxyquinolin-2-yl)acetic acid. When formaldehyde was changed to benzaldehyde, the formation of bisquinolines was observed again, but altering the reaction conditions did not lead to a Mannich reaction, but a Knoevenagel condensation took place. To investigate the scope and limitation of the condensation, a series of *para*-substituted (nitro-, fluoro-, methyl-, methoxy-, dimethylamino-) and *ortho*-(hydroxy)-substituted benzaldehydes as well as 1- and 2-naphthaldehyde were applied. When the reaction was carried out with salicylaldehyde, an intramolecular ring closure took place affording a lactone-containing skeleton, which is a 4-hydroxyquinoline–coumarin hybrid. Benzocoumarin derivatives were also synthesised using the appropriate hydroxynaphthaldehydes.

Regarding the biological results, it can be concluded that some derivatives possess selectivity towards cancer cells and these scaffolds may be interesting to develop further MDR tumour selective compounds. The pIC_50_ values and the Hammett–Brown substituent showed a good correlation; therefore the results might be used to predict the activity of further compounds.

## Figures and Tables

**Table 1:** Reaction conditions (*i*) for the synthesis of **4a,b** and **5**.

Product	Time (min)	Temperature (°C)	Yield of 4a/4b (%)	Yield of 5 (%)
**4a**	20	240	28	0
	20	245	31	0
	20	250	27	0
	20	245	0	53 ^1^
**4b**	20	220	7	0.5
	20	230	16	0.6
	20	235	28	0.3
	20	240	35	0.1
	20	245	41	0
	20	250	35	0
	15	245	37	0
	30	245	36	0

^1^ With added *p*-TSA.

**Table 2:** Cytotoxic activity of the synthesised compounds on Colo 205 and Colo 320 cell lines, and normal human embryonic MRC-5 fibroblasts.

Compound	Colo 205 IC_50_ (µM)		Colo 320 IC_50_ (µM)		MRC-5 IC_50_ (µM)	
	Mean	SD (+/−)	Mean	SD (+/−)	Mean	SD (+/−)
**4a**	>100	−	92.78	1.68	>100	−
**4b**	>100	−	>100	−	>100	−
**5**	>100	−	>100	−	>100	−
**7**	>100	−	>100	−	>100	−
**8**	>100	−	>100	−	>100	−
**9**	>100	−	>100	−	>100	−
**10a**	>100	−	>100	−	>100	−
**10b**	>100	−	>100	−	>100	−
**12**	>100	−	>100	−	>100	−
**13a**	11.86	1.07	8.19	1.35	28.56	1.11
**13b**	8.10	0.11	4.58	0.18	18.94	1.83
**14**	>100	−	>100	−	>100	−
**20**	2.34	0.24	4.61	0.24	9.89	0.00
**21**	16.54	1.97	42.65	2.26	21.94	0.82
**22**	11.79	0.27	12.29	0.55	30.64	0.64
**23**	42.76	2.35	>100	−	65.18	1.13
**24**	>100	−	>100	−	>100	−
**25**	>100	−	32.40	3.37	36.54	1.01
**26**	12.63	0.49	11.00	0.37	17.58	0.27
**28**	>100	−	14.08	0.35	6.52	0.46
**29**	>100	−	9.86	0.90	>100	−
**30**	>100	−	>100	−	>100	−
DOXO	2.30	0.12	3.61	0.34	>10	−

DOXO: doxorubicin.

**Table 3:** The selectivity index of the synthesised compounds based on their IC_50_.

Compound	SI (Selectivity Index)	
	MRC-5/Colo 205	MRC-5/Colo 320
**4a**	-	-
**4b**	-	-
**5**	-	-
**7**	-	-
**8**	-	-
**9**	-	-
**10a**	-	-
**10b**	-	-
**12**	-	-
**13a**	2.41	3.49
**13b**	2.34	4.14
**14**	-	-
**20**	4.23	2.15
**21**	1.33	0.51
**22**	2.60	2.49
**23**	1.52	-
**24**	-	-
**25**	-	1.13
**26**	1.39	1.60
**28**	-	0.46
**29**	-	>6
**30**	-	-

Selectivity index = IC_50 (MRC-5)_/IC_50 (Colo205)_ or IC_50 (MRC-5)_/IC_50 (Colo320)._

**Table 4:** The Hammett–Brown constant (*σ_p_*^+^) for substituents (X) at the *para* position and the corresponding negative logarithm of IC_50_ (pIC_50_).

Compound	X	*σ_p_* ^+^	Colo 205 pIC_50_	Colo 320 pIC_50_
**20**	NO_2_	0.79	5.63	5.34
**13b**	H	0.00	5.09	5.34
**21**	F	–0.07	4.78	4.37
**22**	Me	–0.31	4.93	4.91
**23**	OMe	–0.78	4.37	<4.00
**24**	NMe_2_	–1.70	<4.00	<4.00

## Supplementary Materials

The following supporting information can be downloaded at: https://www.mdpi.com/article/10.3390/ijms23179688/s1.
