# Supplementary material for: Synthesis of 4-Hydroxyquinolines as Potential Cytotoxic Agents
Source: Int J Mol Sci. 2022 Aug 26;23(17):9688. doi: 10.3390/ijms23179688 (PMC9456289; doi:10.3390/ijms23179688)
Supplement: Supplementary file 1 [file ijms-23-09688-s001.zip › ijms-1863920-supplementary.pdf]

# Synthesis of 4-Hydroxyquinolines as Potential Cytotoxic Agents

**Oszkár Csuvik [1], Nikoletta Szemerédi [2], Gabriella Spengler [2] and István Szatmári [1,3,*]**

[1] Institute of Pharmaceutical Chemistry, University of Szeged, Eötvös u. 6, H-6720 Szeged, Hungary
[2] Department of Medical Microbiology, Albert Szent-Györgyi Health Center and Albert Szent-Györgyi Medical School, University of Szeged, Semmelweis u. 6, H-6725 Szeged, Hungary
[3] Stereochemistry Research Group, Eötvös Loránd Research Network, University of Szeged, Eötvös u. 6, H-6720 Szeged, Hungary
* Correspondence: szatmari.istvan@szte.hu

**Table S1. Physical-chemical properties of the synthesised 4-hydroxyquinolines.**

| Compound | Melting point (°C) | Molecular weight [M + $H^+$] |
|---|---|---|
| **4a** | 211–214 | 218.0808 |
| **4b** | 208–210 | 232.0964 |
| **5** | 294–297 | 279.1126 |
| **7** | >350 | 331.1441 |
| **12** | 199–202 | 407.1756 |
| **8** | 221–224 | 204.0659 |
| **9** | >350 | 269.1651 |
| **10a** | 166–169 | 327.1702 |
| **10b** | 152–155 | 341.1854 |
| **13a** | 247–251 | 306.1119 |
| **13b** | 222–225 | 320.1277 |
| **20** | 257–260 | 365.1129 |
| **21** | 233–236 | 338.1182 |
| **22** | 224–227 | 334.1434 |
| **23** | 215–218 | 350.1384 |
| **24** | 256–259 | 363.1700 |
| **25** | 262–265 | 370.1435 |
| **26** | 177–180 | 370.1434 |
| **28** | 315–319 | 290.0808 |
| **29** | 295–298 | 340.0966 |
| **30** | >350 | 340.0966 |
| **14** | 230–233 | 292.0968 |

### $^1$H NMR, $^{13}$C NMR, and HRMS spectra of synthesised compounds

**Methyl 2-(4-hydroxyquinolin-2-yl) acetate (4a)**

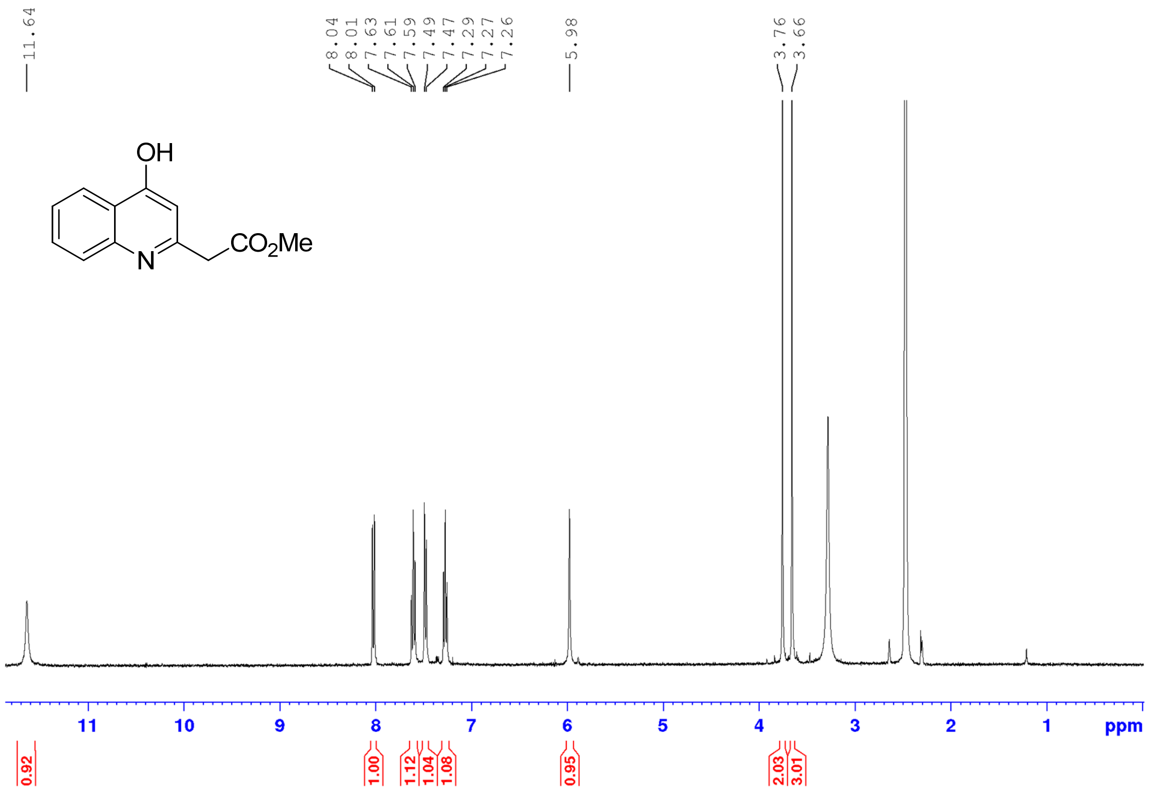


Figure S1. $^1$H NMR spectrum of **4a**

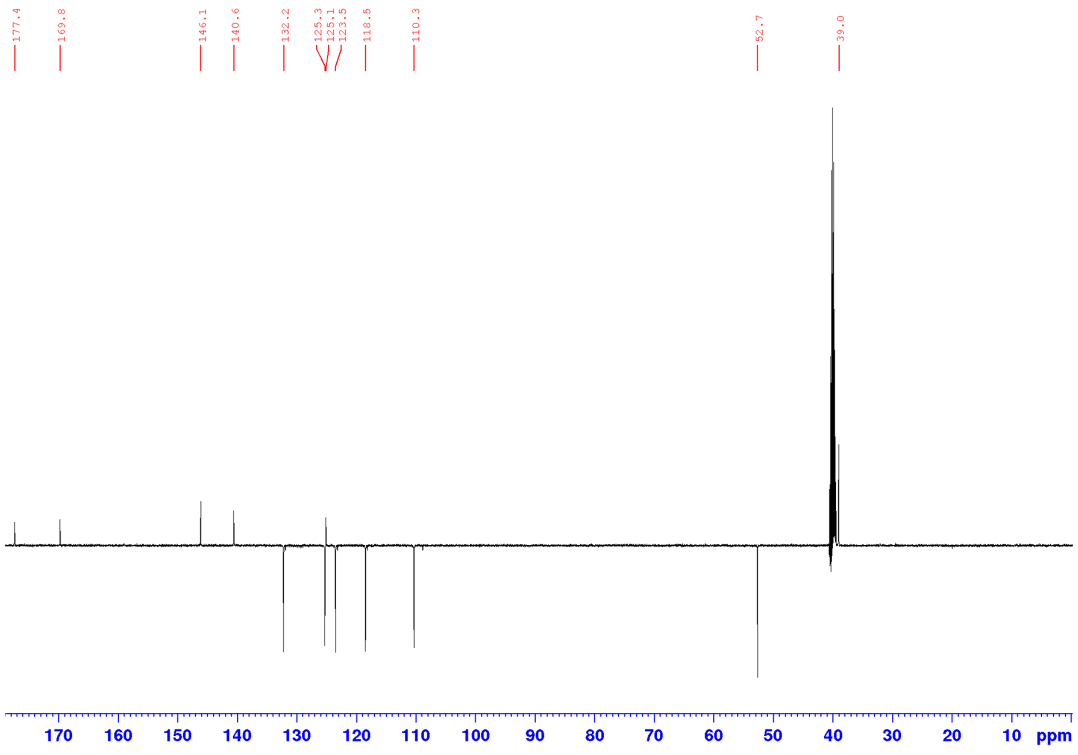


Figure S2. $^{13}$C NMR spectrum of **4a**

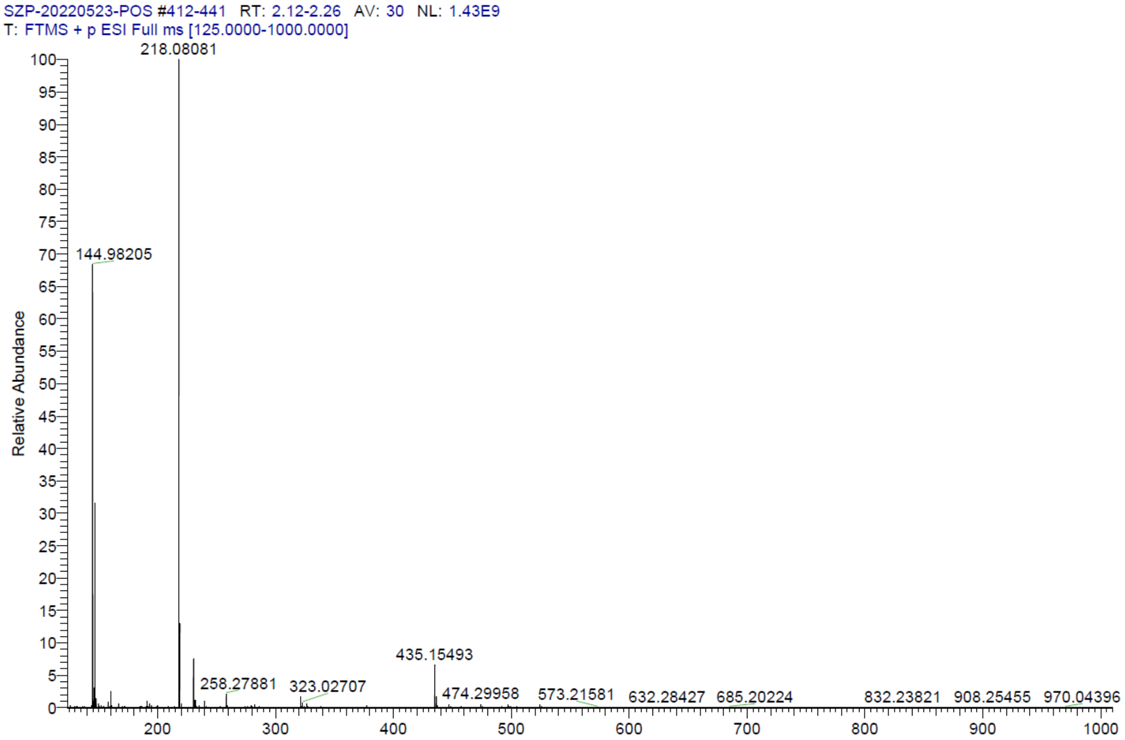


Figure S3. HRMS spectrum of **4a**

**Ethyl 2-(4-hydroxyquinolin-2-yl) acetate (4b)**

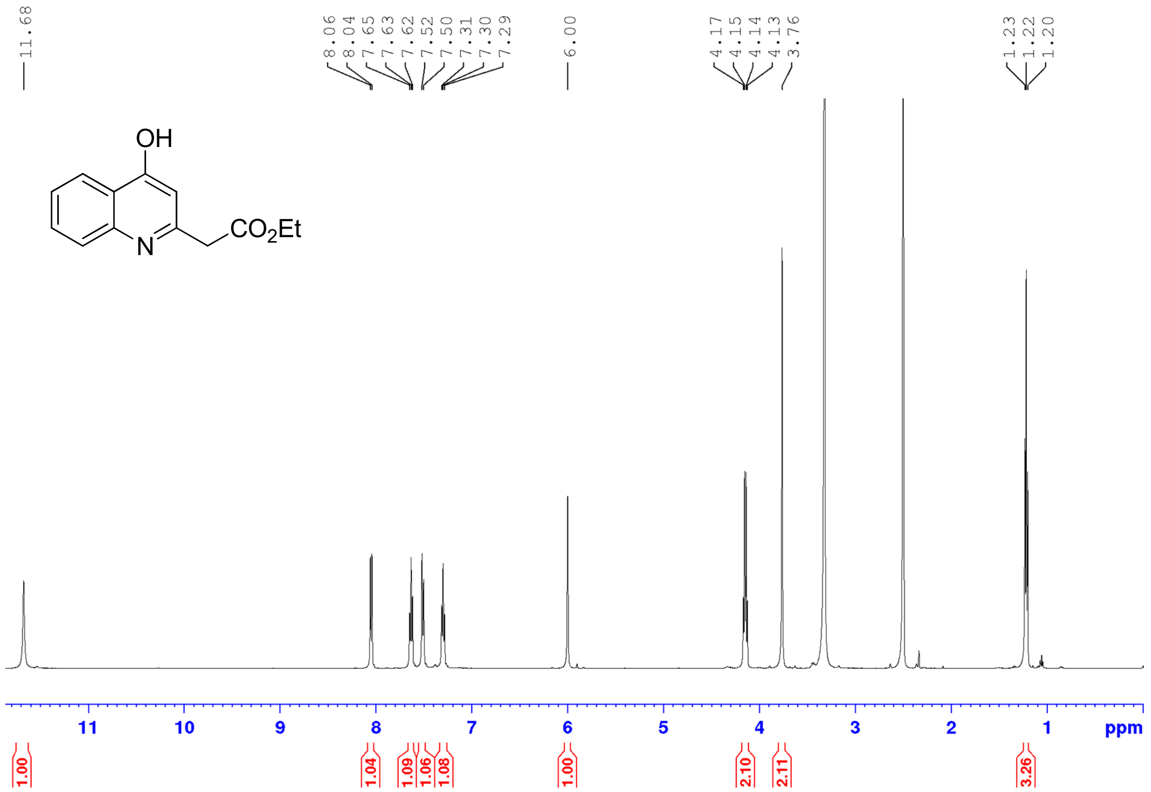


Figure S4. $^{1}$H NMR spectrum of **4b**

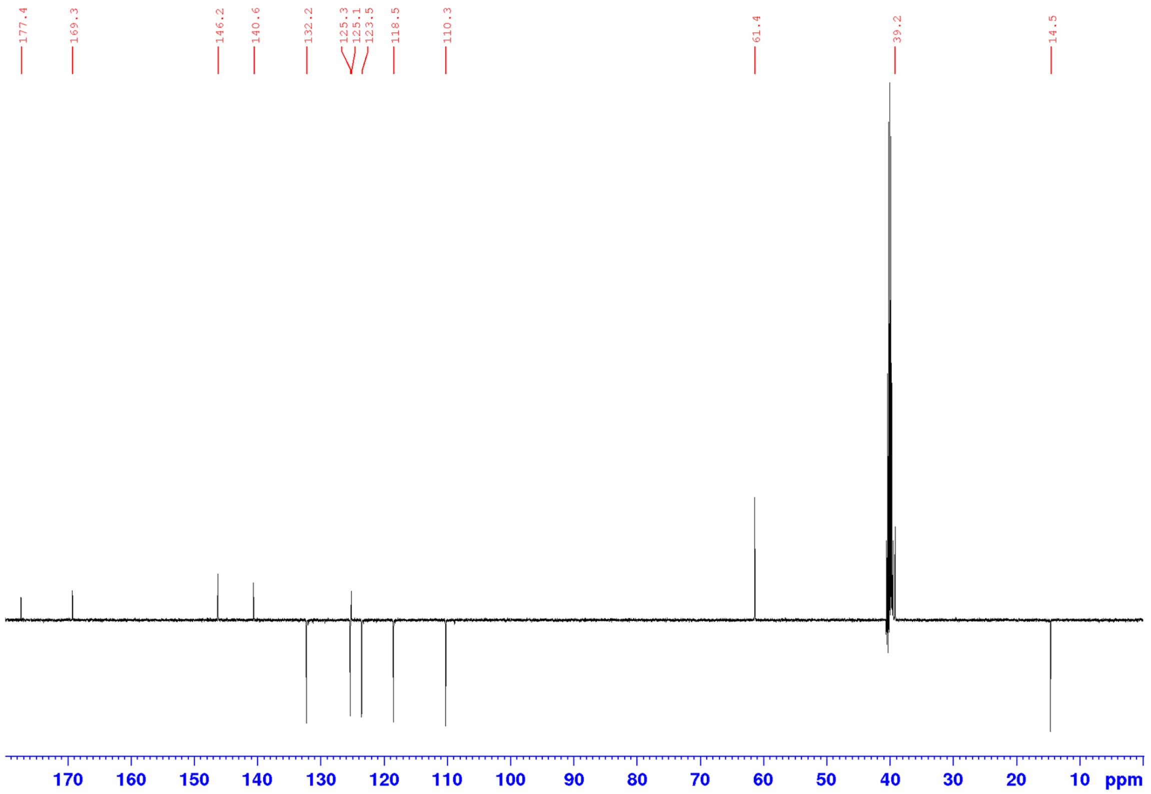


Figure S5. $^{13}$C NMR spectrum of **4b**

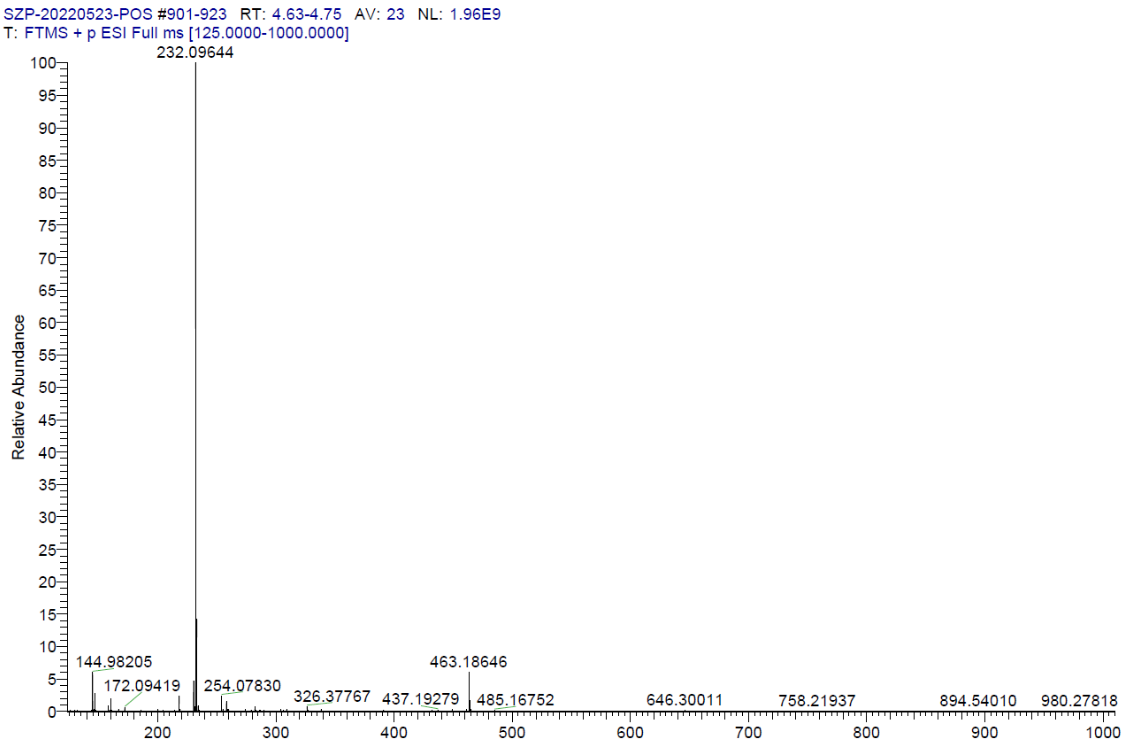


Figure S6. HRMS spectrum of **4b**

**1-Phenyl-4-(phenylamino)pyridine-2,6(1*H*,3*H*)-dione (5)**

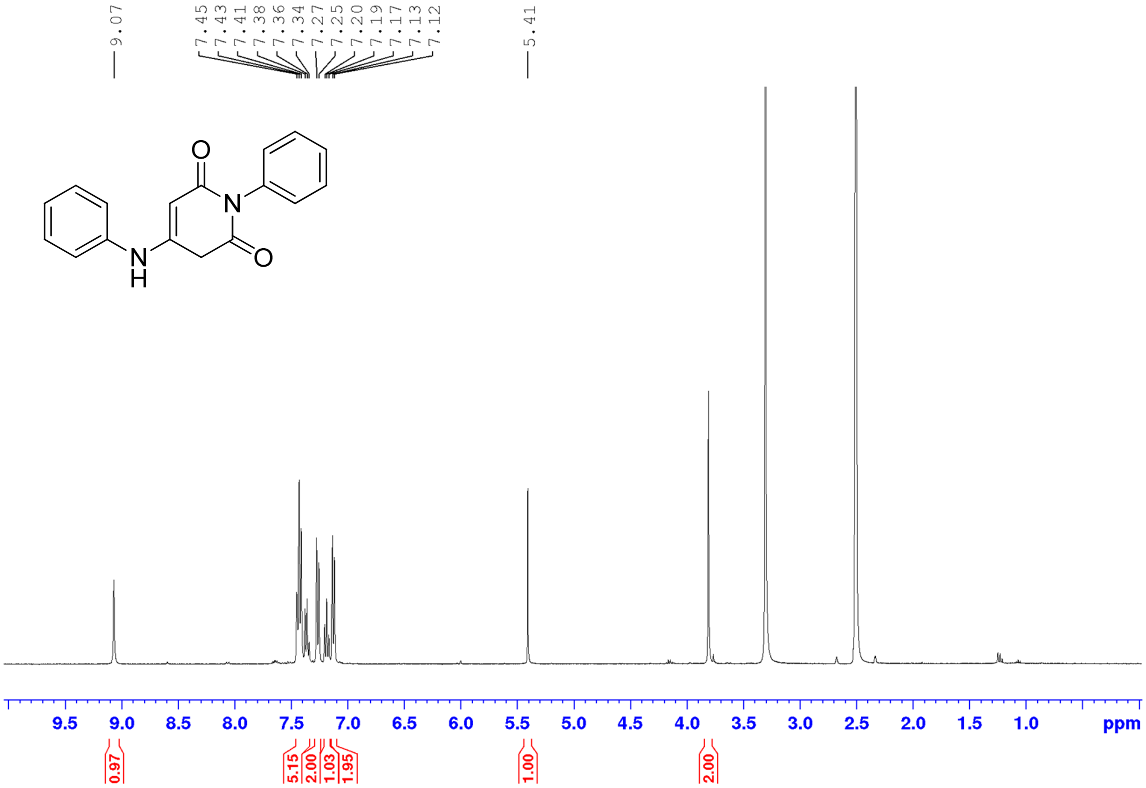


Figure S7. $^{1}$H NMR spectrum of **5**

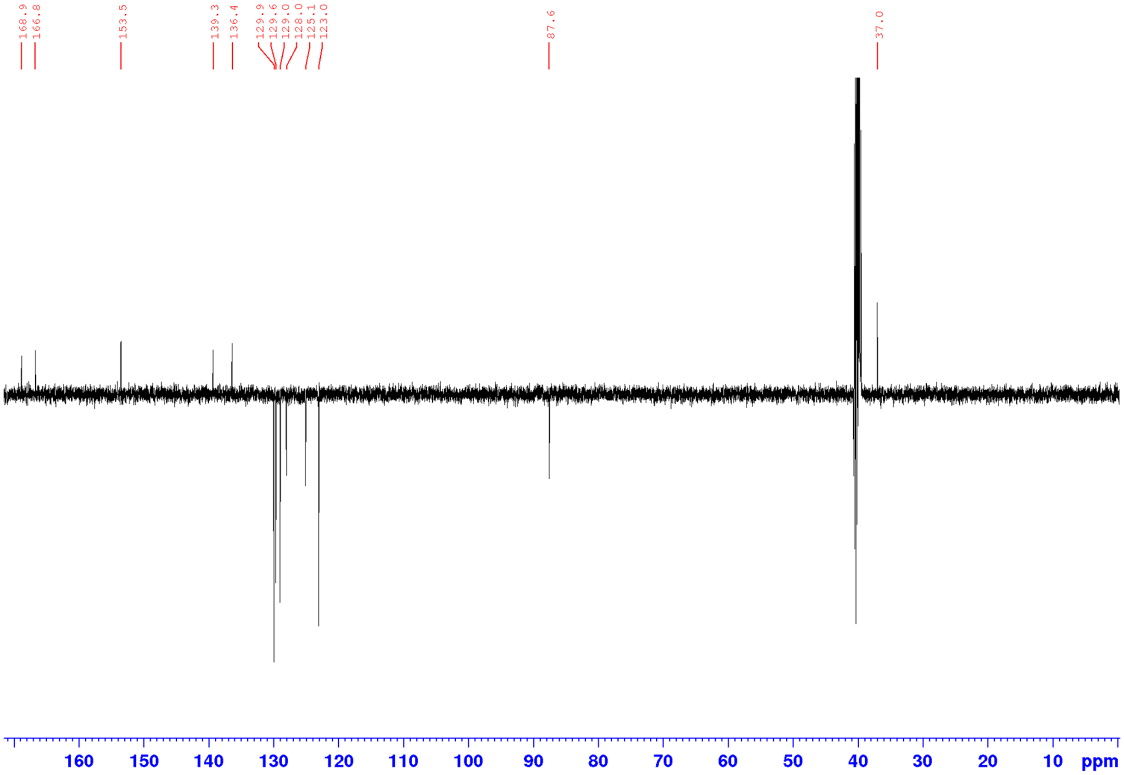


Figure S8. $^{13}$C NMR spectrum of **5**

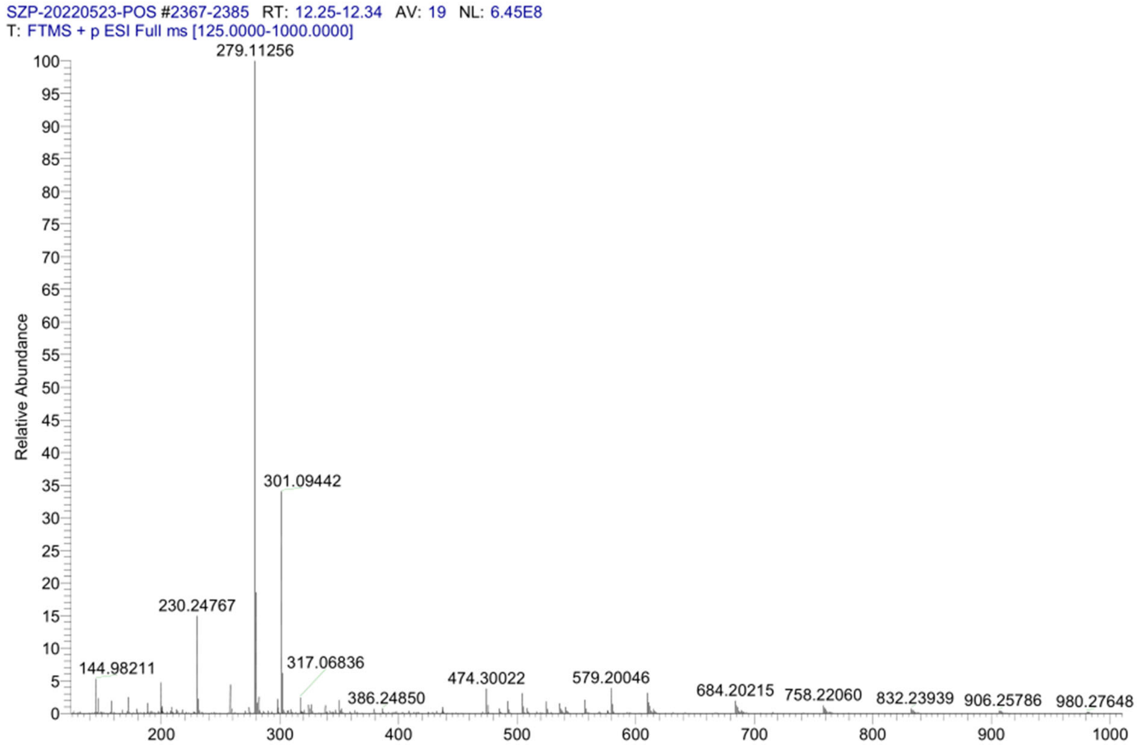


Figure S9. HRMS spectrum of **5**

**2,2'-(Propane-1,3-diyl)bis(quinolin-4-ol) (7)**

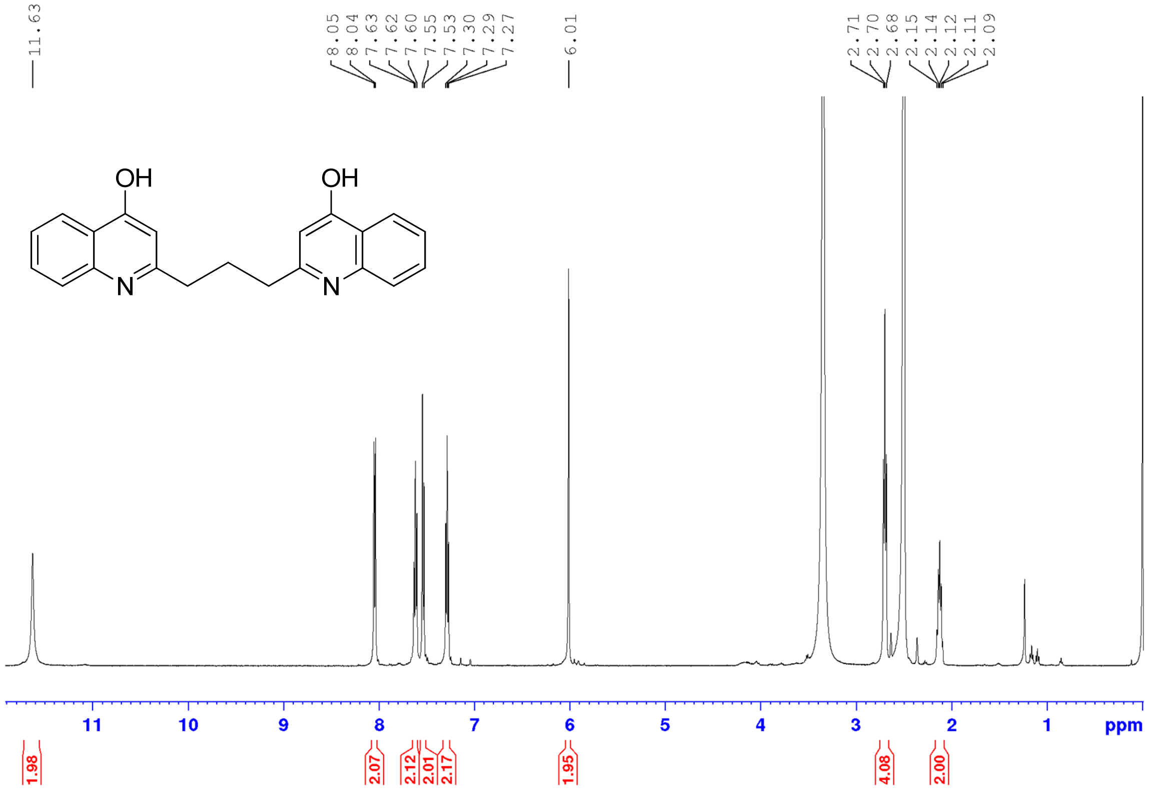


Figure S10. $^{1}$H NMR spectrum of **7**

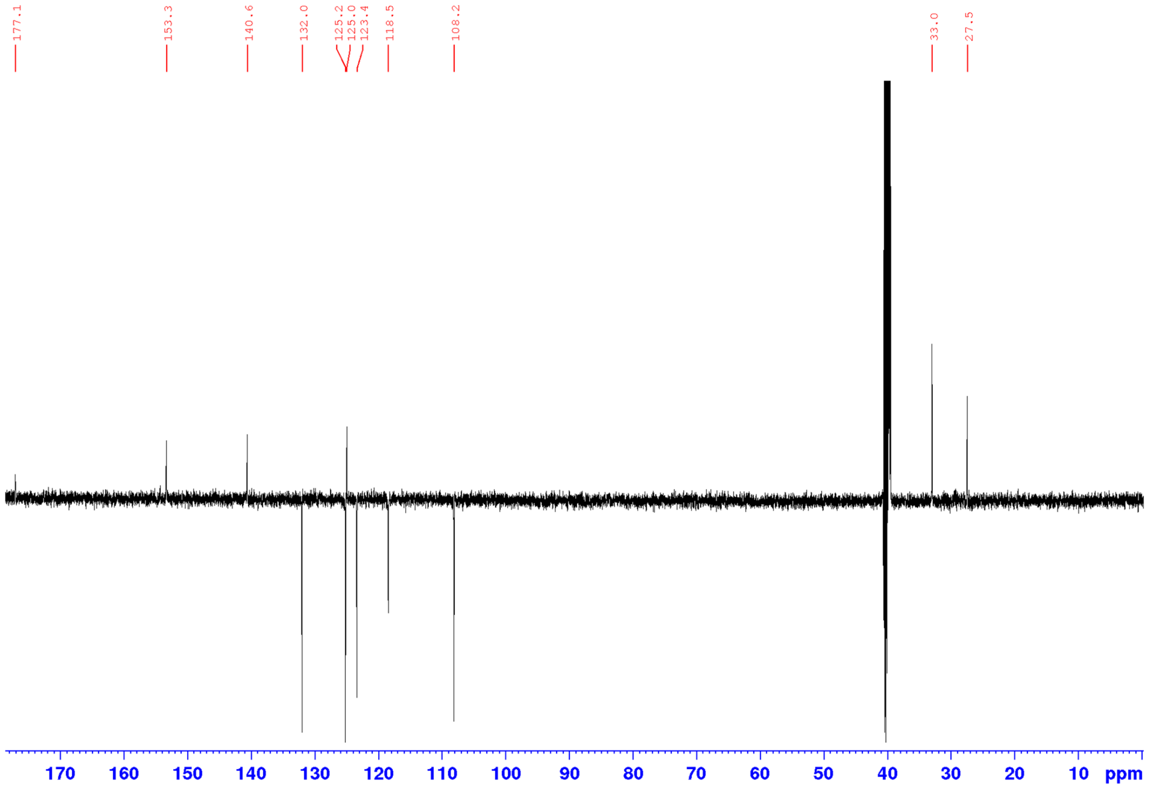


Figure S11. $^{13}$C NMR spectrum of **7**

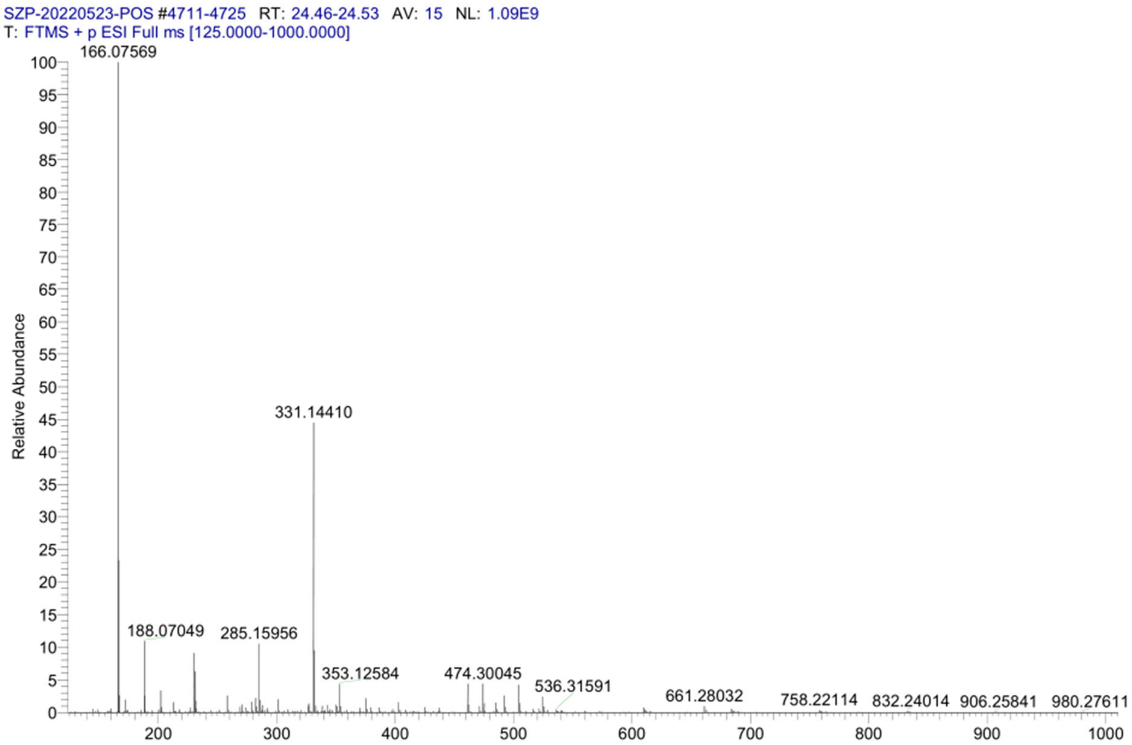


Figure S12. HRMS spectrum of **7**

**2,2'-(2-Phenylpropane-1,3-diyl)bis(quinolin-4-ol) (12)**

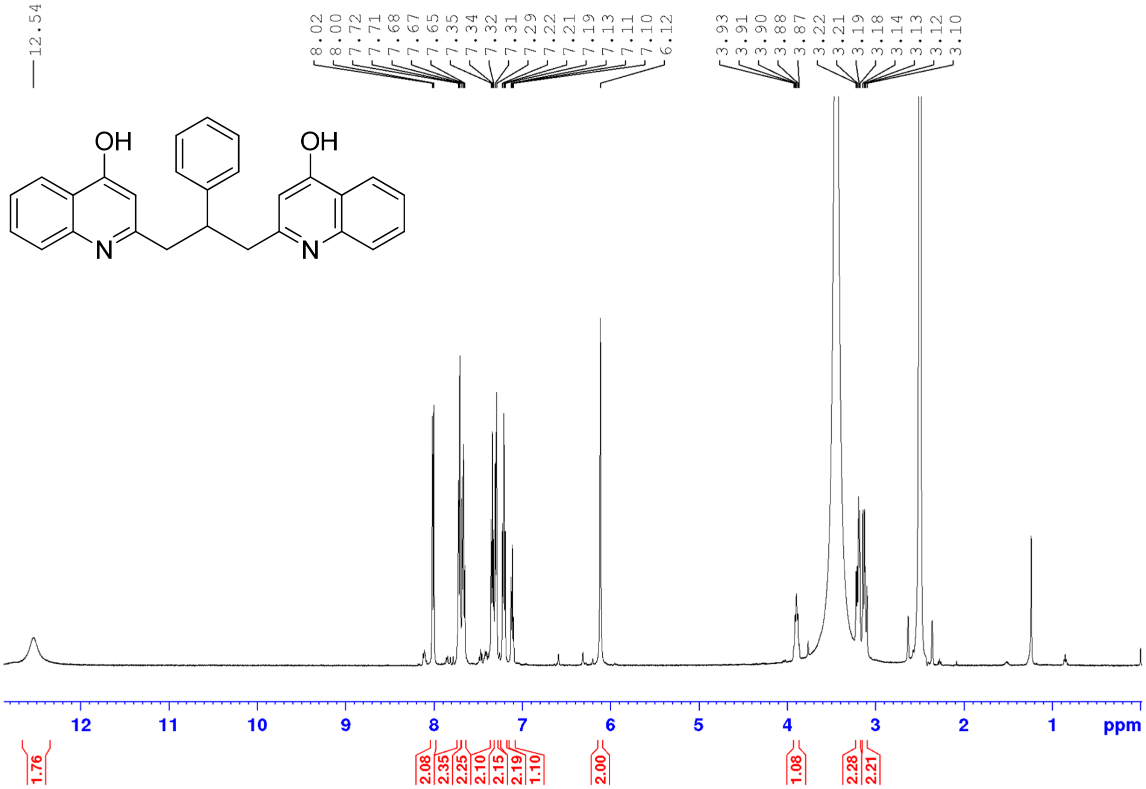


Figure S13. $^{1}$H NMR spectrum of **12**

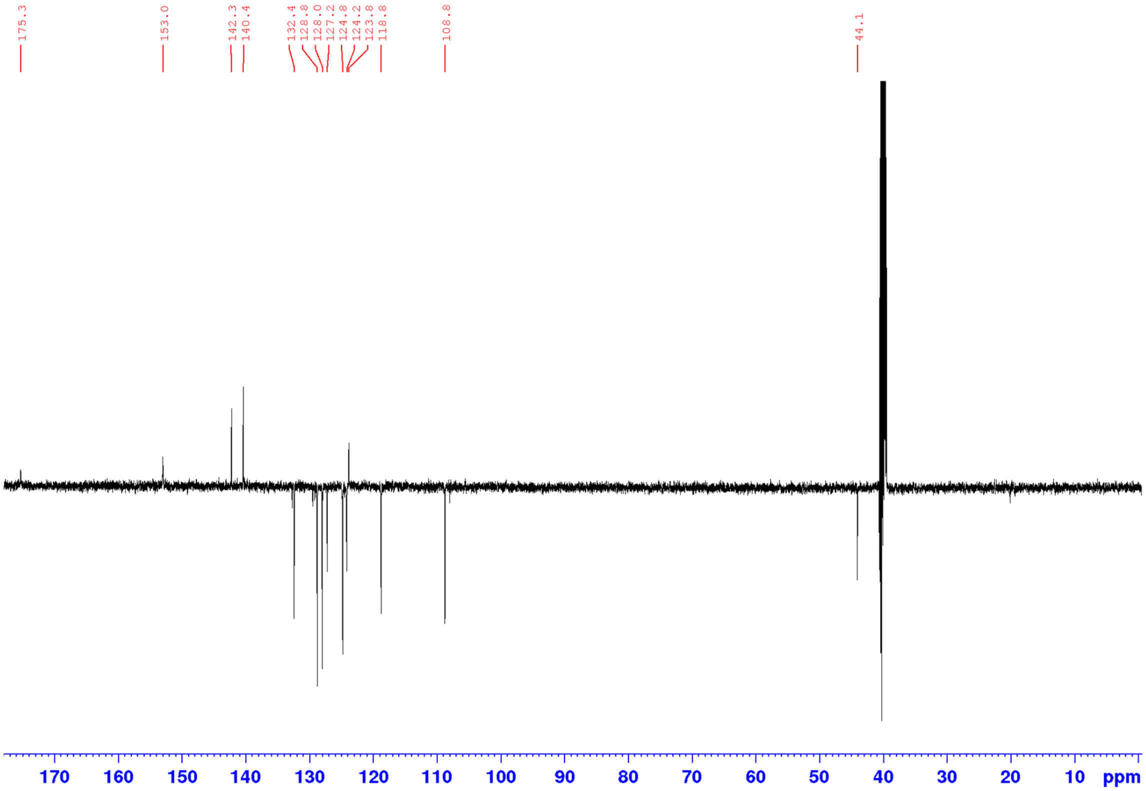


Figure S14. $^{13}$C NMR spectrum of **12**

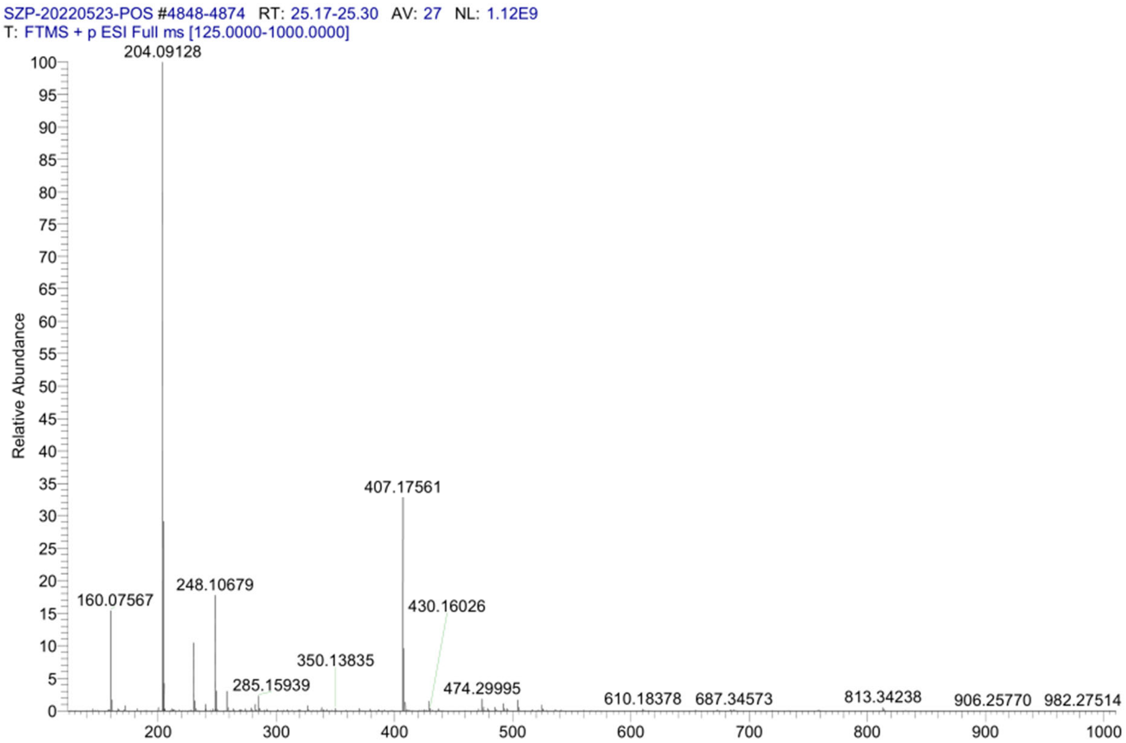


Figure S15. HRMS spectrum of **12**

**2-(4-hydroxyquinolin-2-yl)acetic acid (8)**

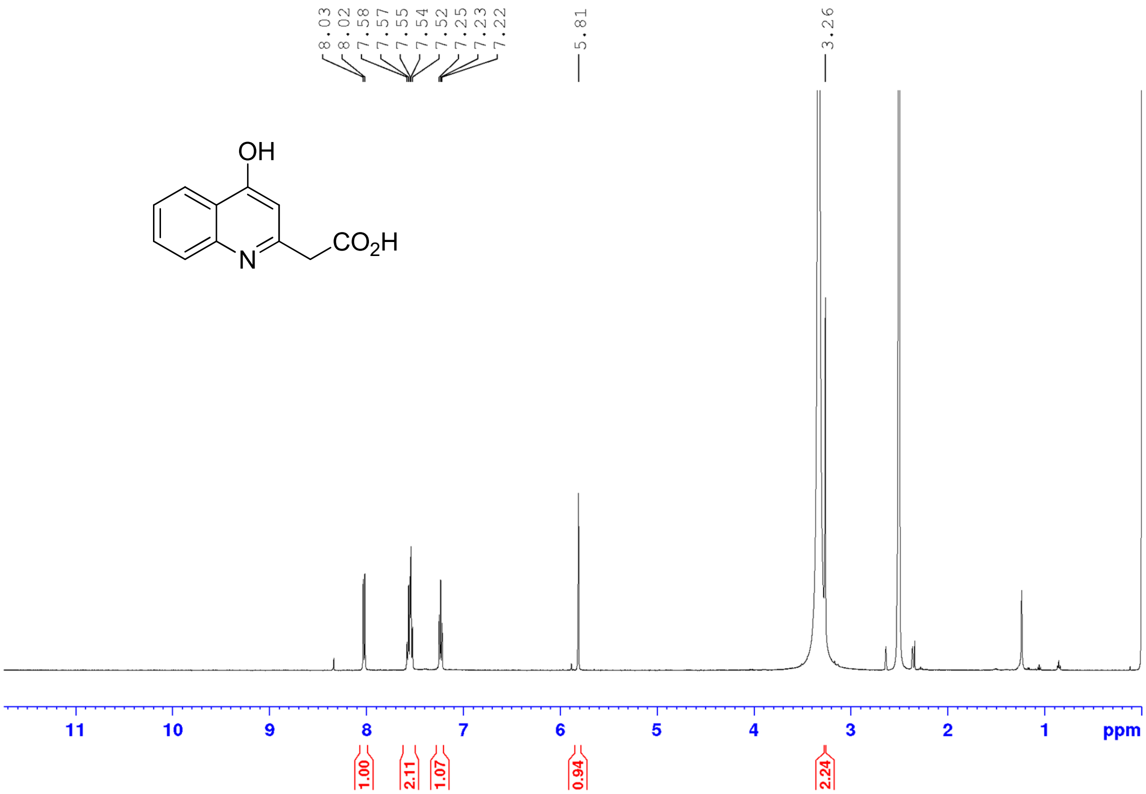


Figure S16. $^{1}$H NMR spectrum of **8**

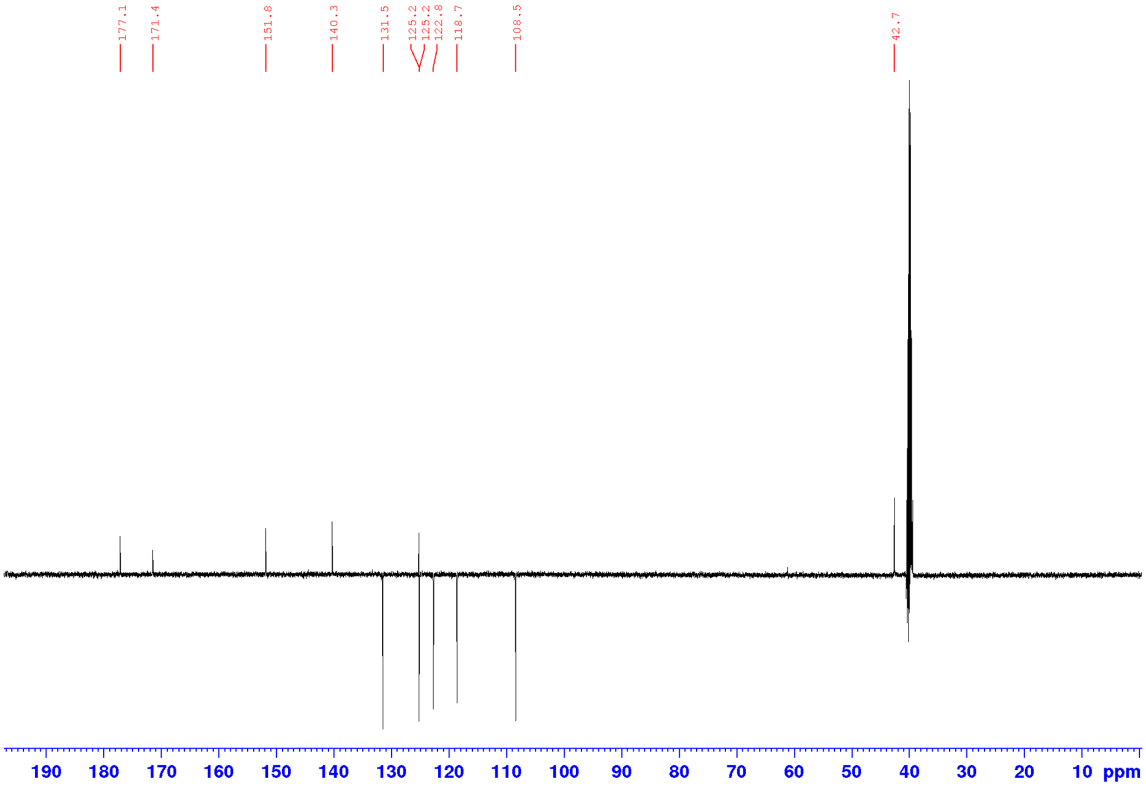


Figure S17. $^{13}$C NMR spectrum of **8**

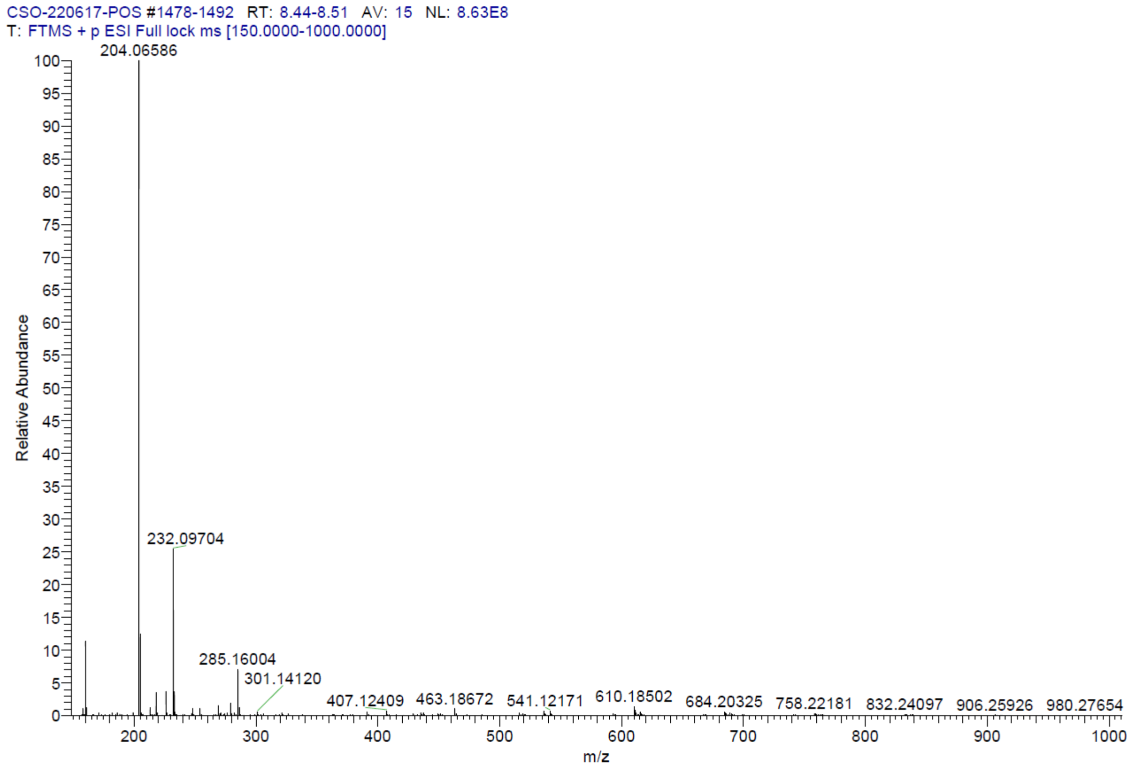


Figure S18. HRMS spectrum of **8**

**3-(Piperidin-1-ylmethyl)-1*H*-azeto[1,2-*a*]quinolin-4(2*H*)-one (9)**

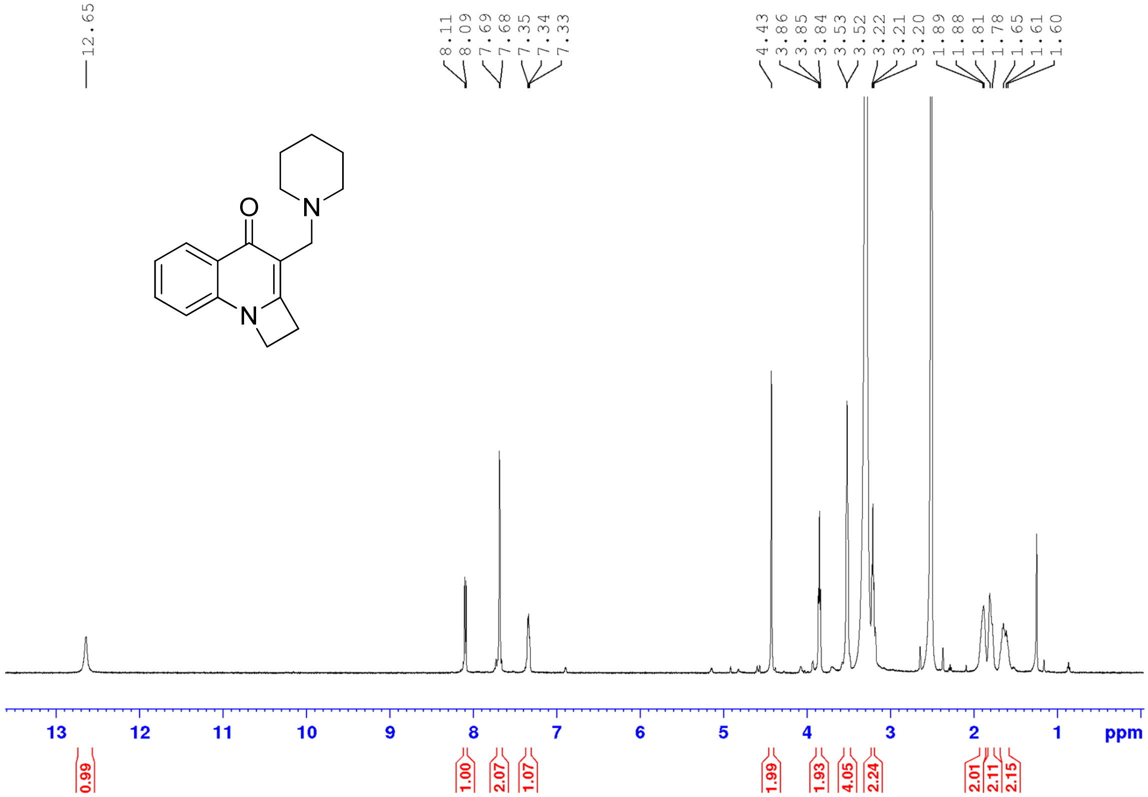


Figure S19. $^{1}$H NMR spectrum of **9**

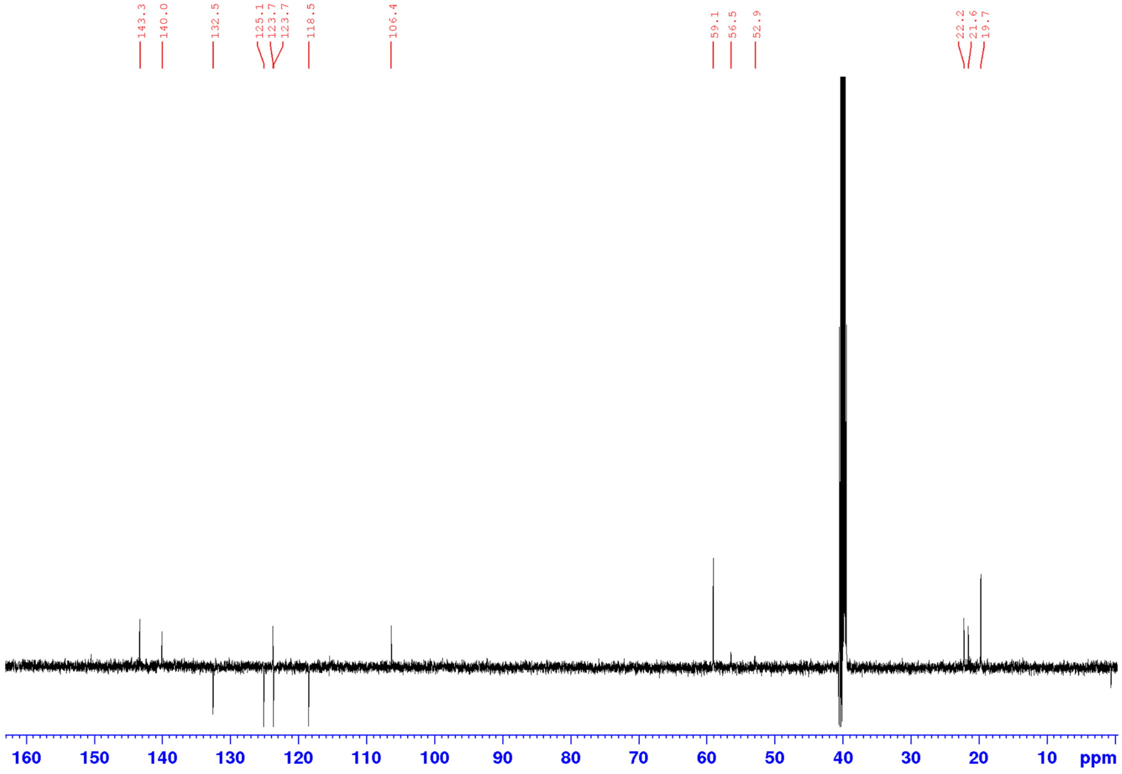


Figure S20. $^{13}$C NMR spectrum of **9**

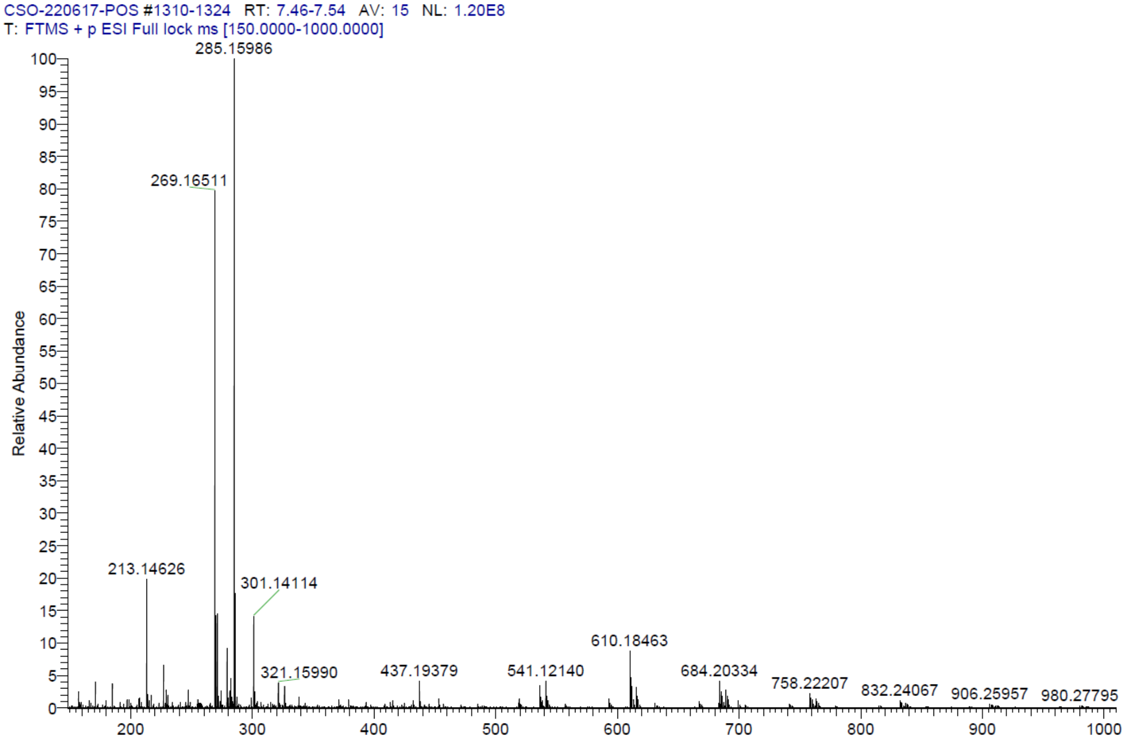


Figure S21. HRMS spectrum of **9**

**Methyl 2-(4-hydroxy-3-(piperidin-1-ylmethyl)quinolin-2-yl) acetate (10a)**

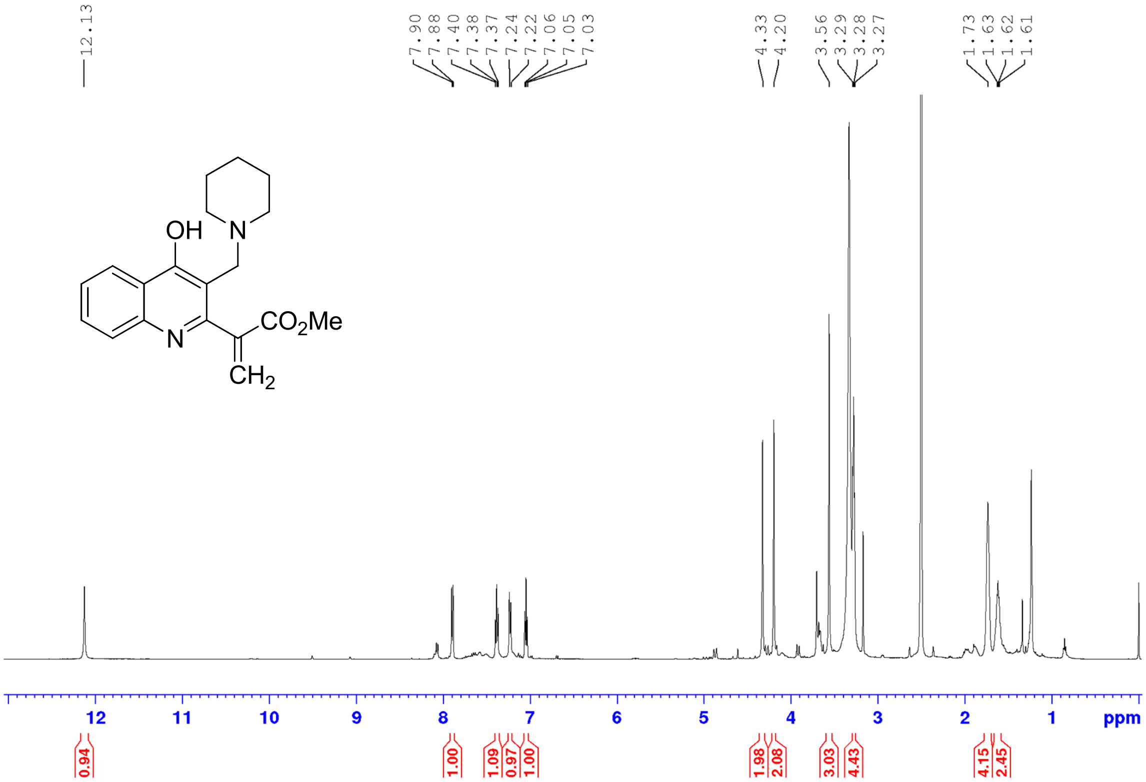


Figure S22. $^{1}$H NMR spectrum of **10a**

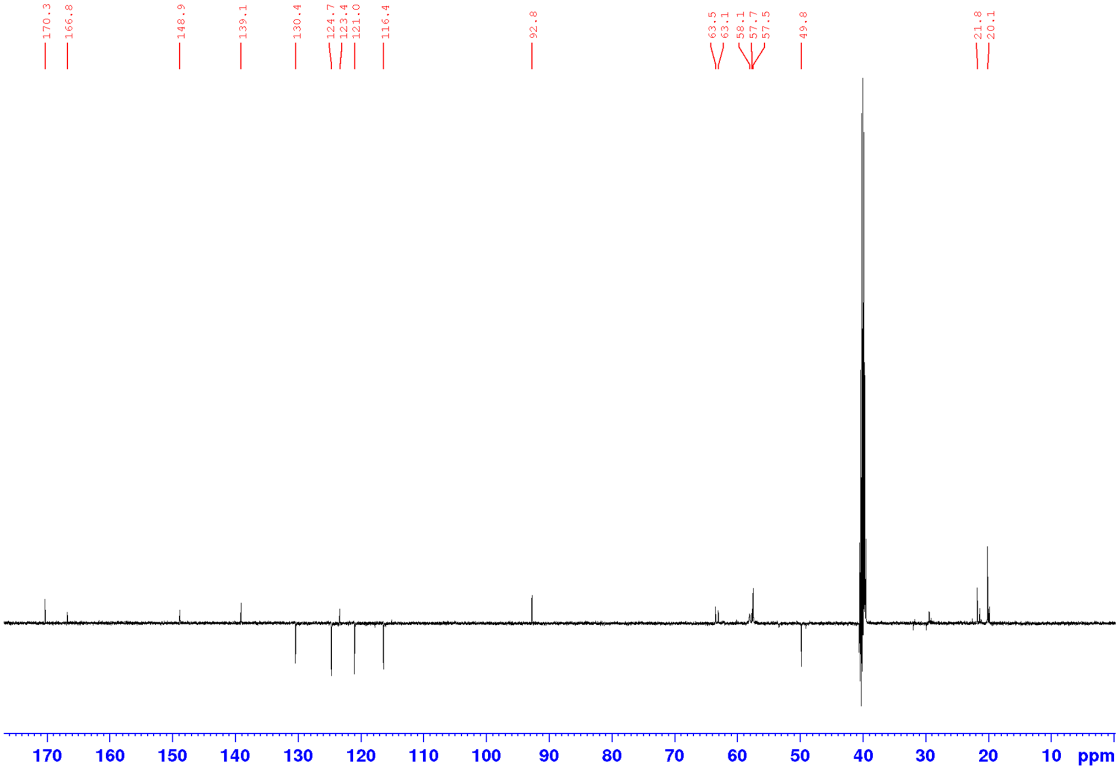


Figure S23. $^{13}$C NMR spectrum of **10a**

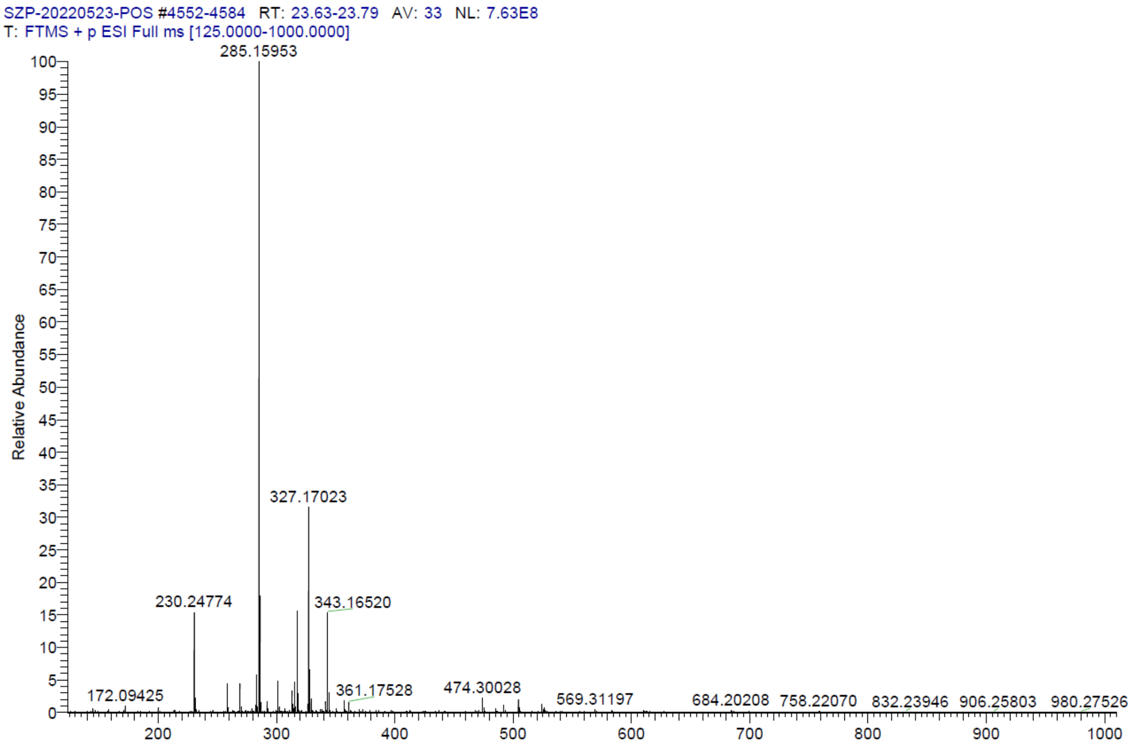


Figure S24. HRMS spectrum of **10a**

**Ethyl 2-(4-hydroxy-3-(piperidin-1-ylmethyl)quinolin-2-yl) acetate (10b)**

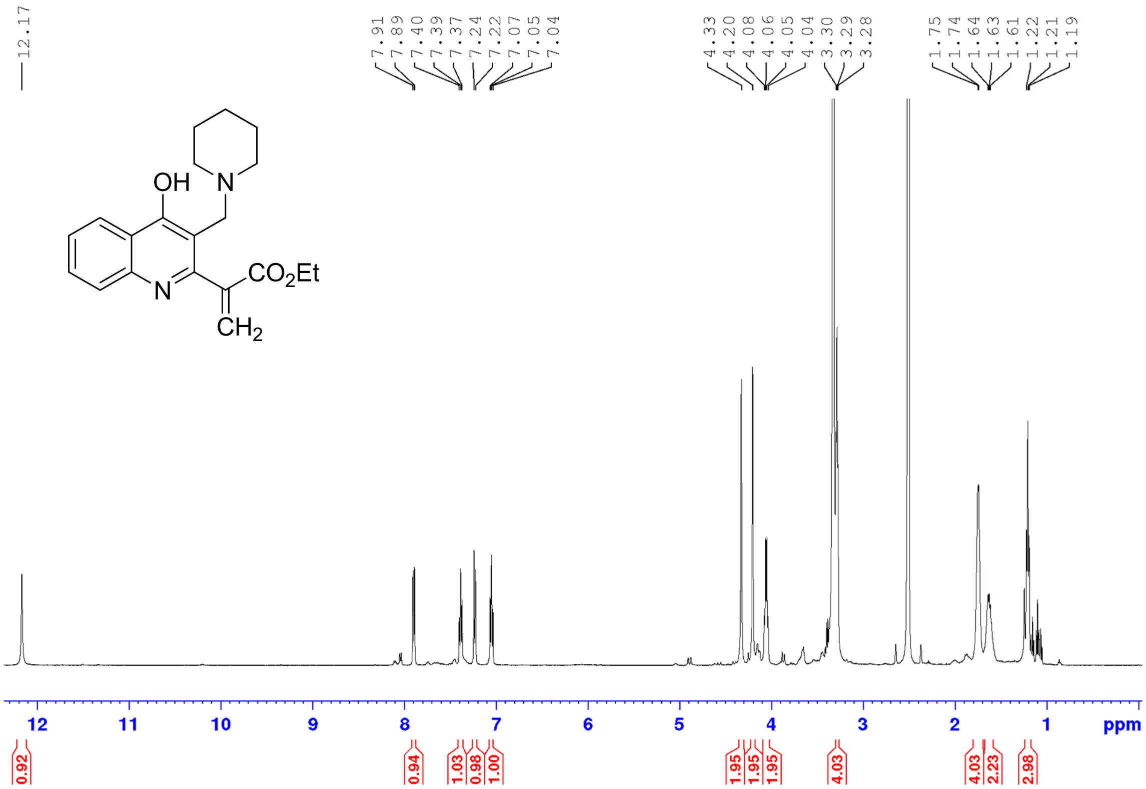


Figure S25. $^{1}$H NMR spectrum of **10b**

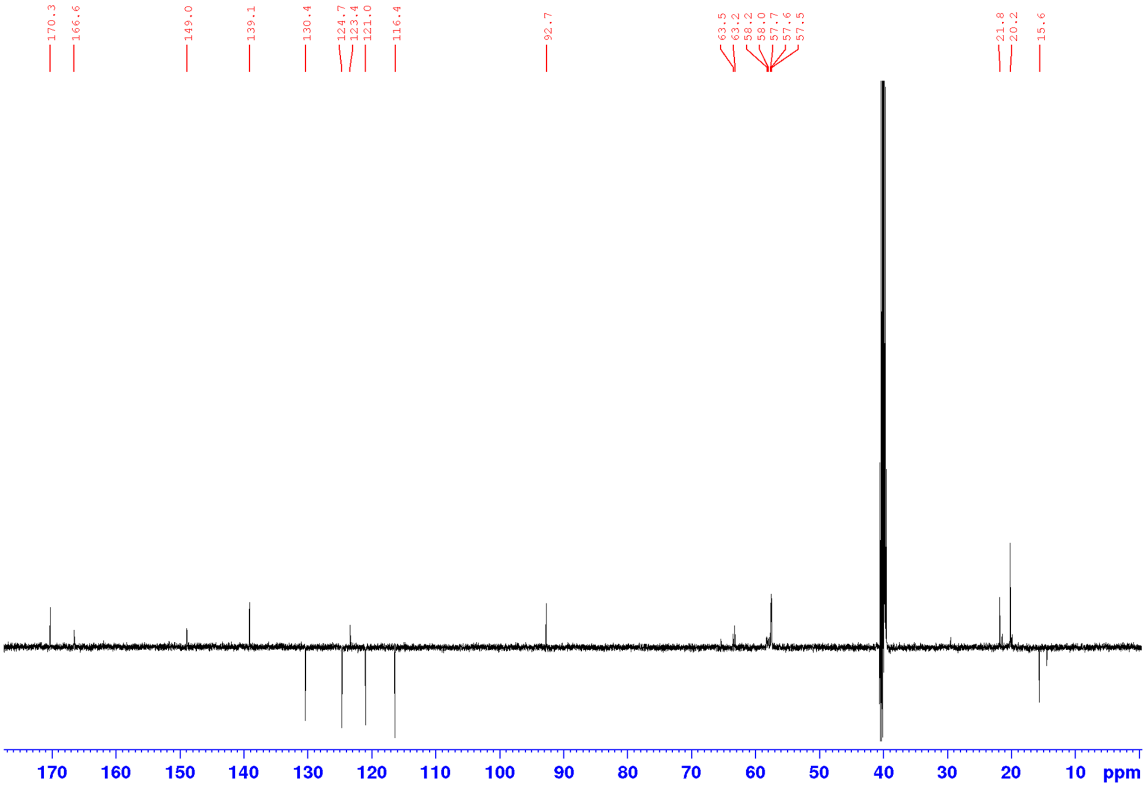


Figure S26. $^{13}$C NMR spectrum of **10b**

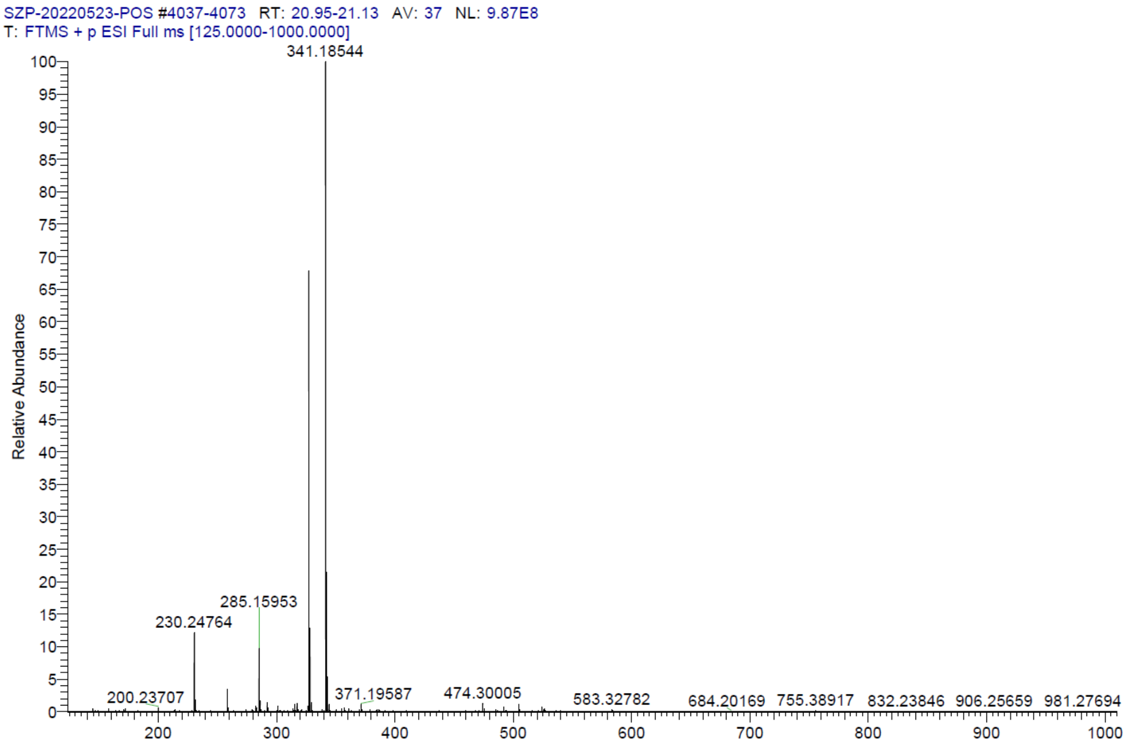


Figure S27. HRMS spectrum of **10b**

**(*Z*)-methyl 2-(4-hydroxyquinolin-2-yl)-3-phenylacrylate (13a)**

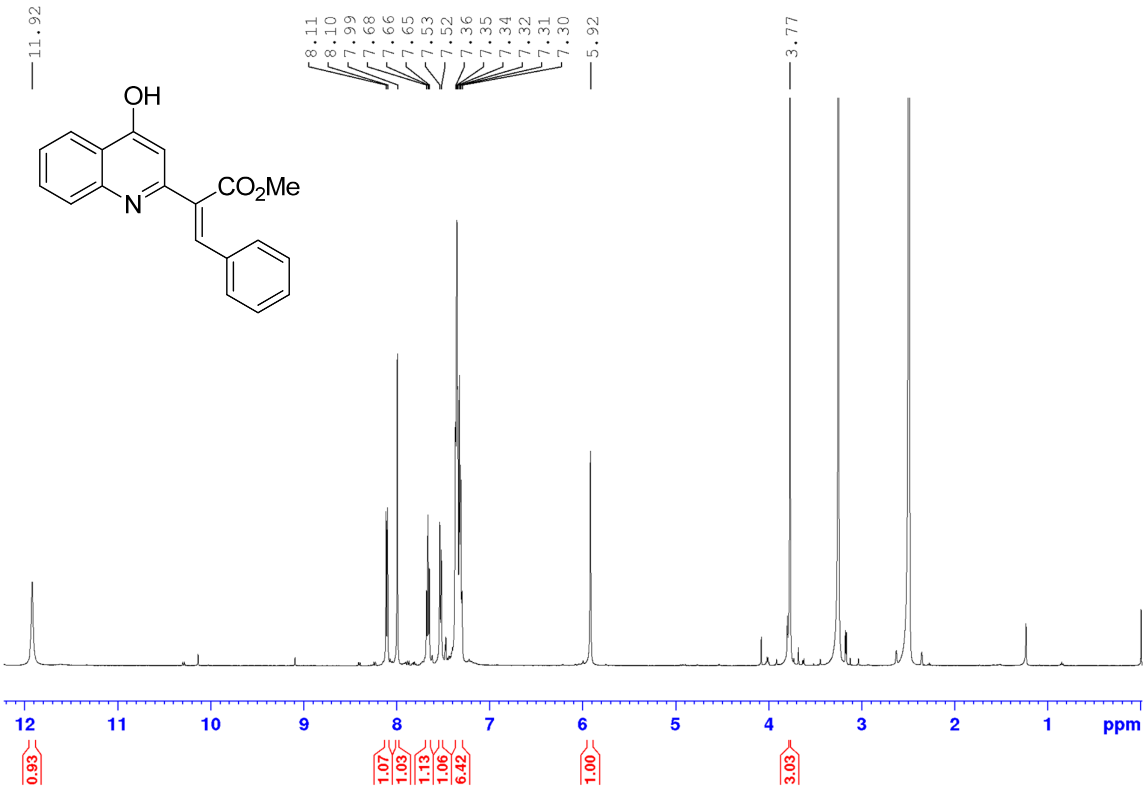


Figure S28. ¹H NMR spectrum of **13a**

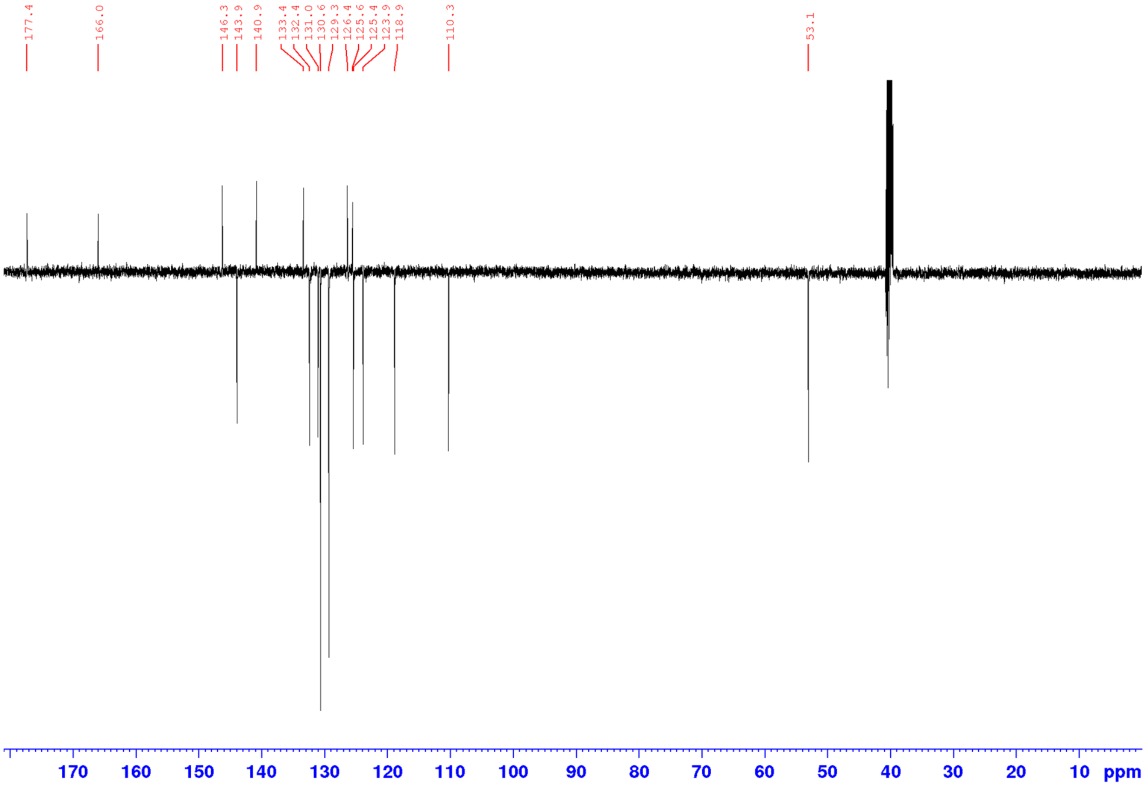


Figure S29. ¹³C NMR spectrum of **13a**

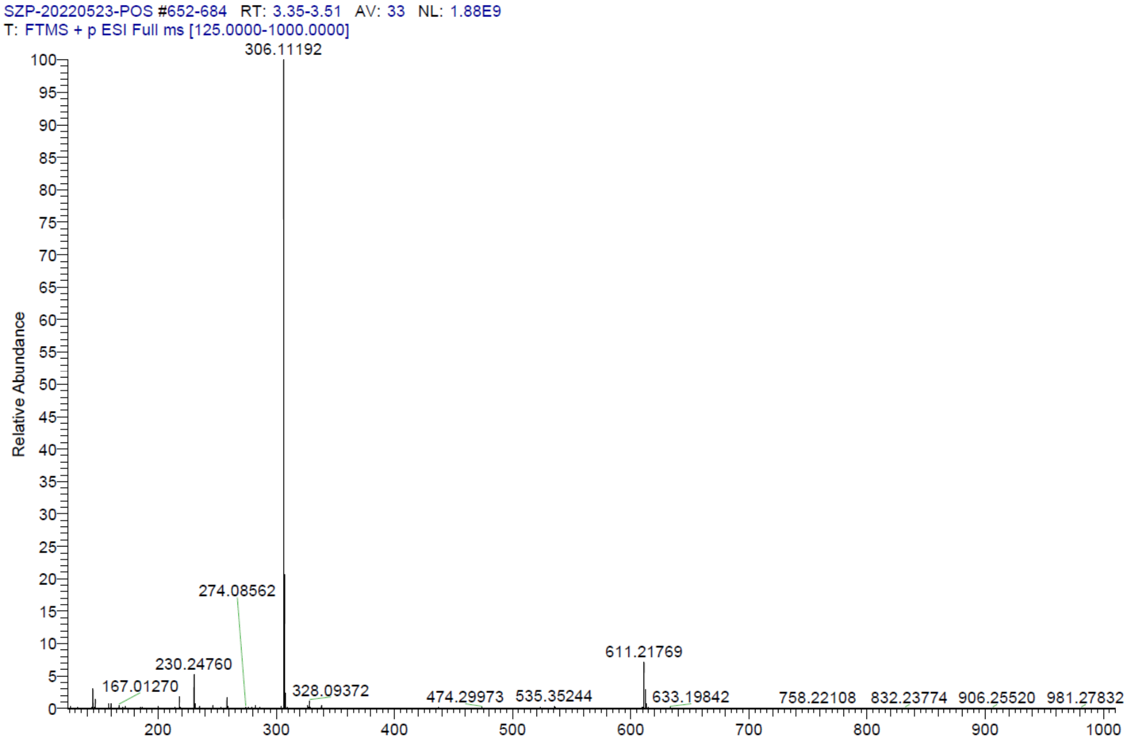


Figure S30. HRMS spectrum of **13a**

**(*Z*)-ethyl 2-(4-hydroxyquinolin-2-yl)-3-phenylacrylate (13b)**

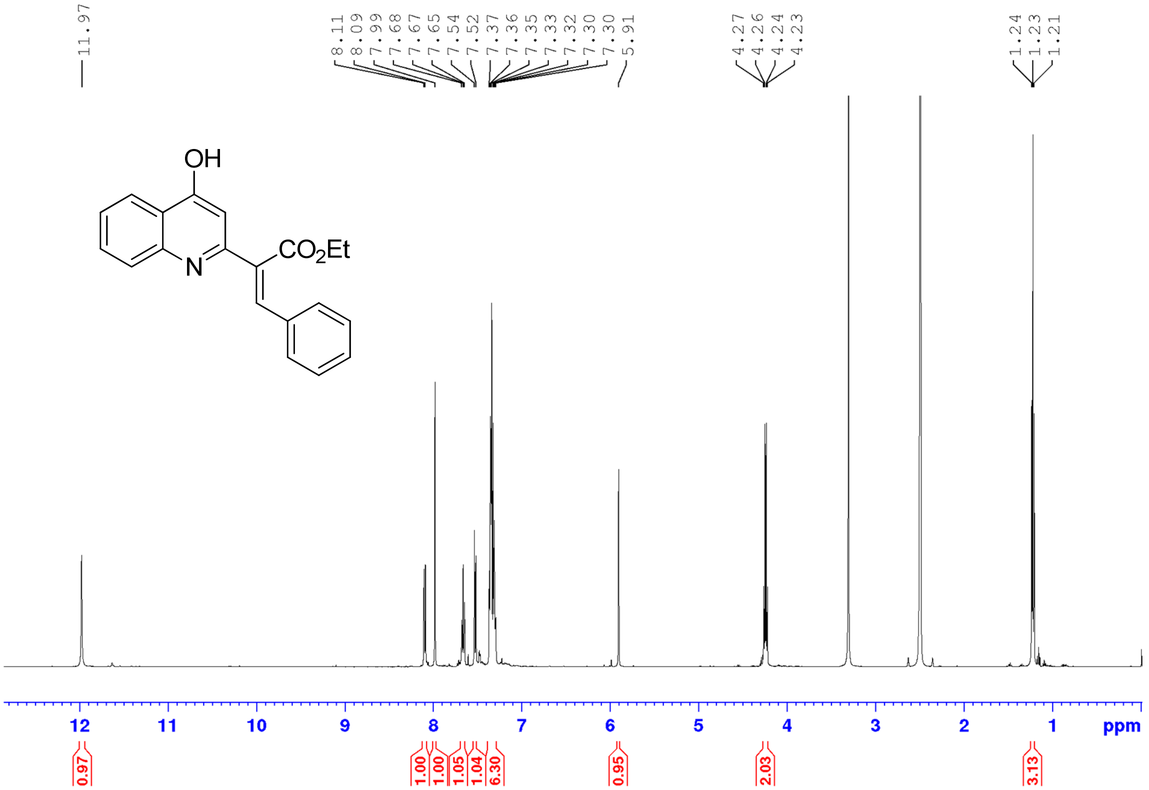


Figure S31. $^{1}$H NMR spectrum of **13b**

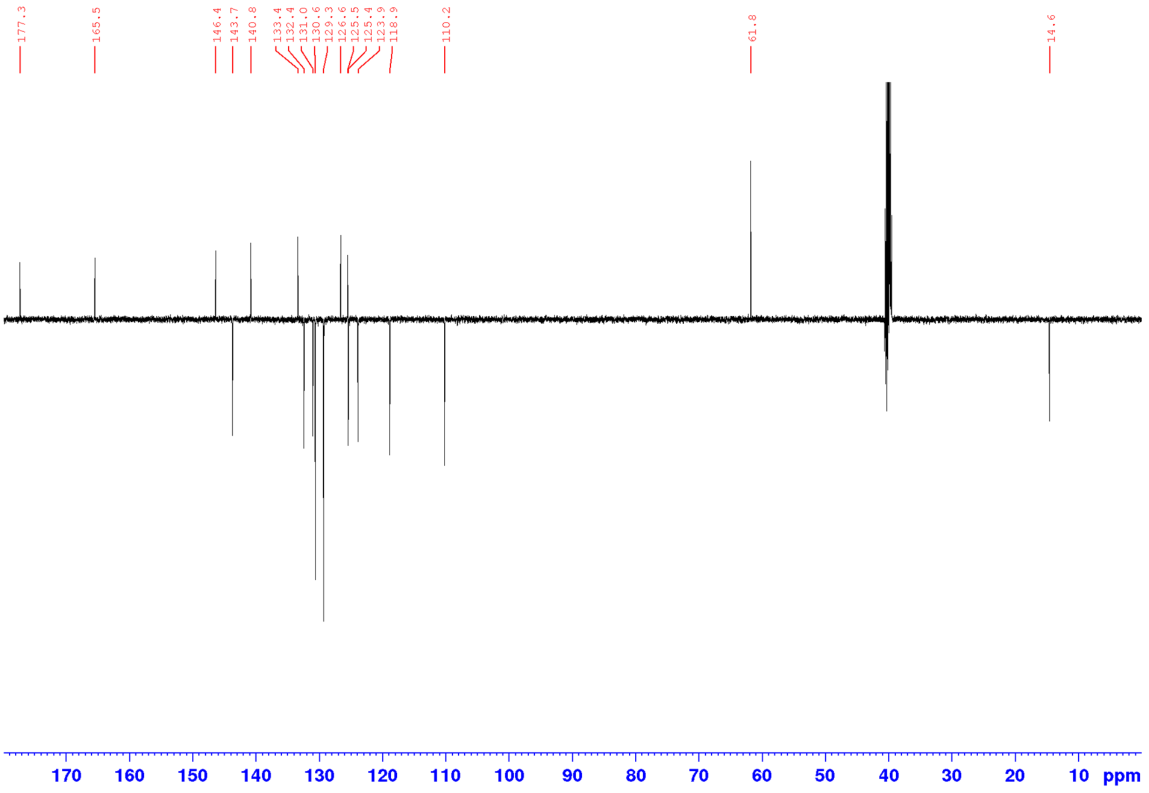


Figure S32. $^{13}$C NMR spectrum of **13b**

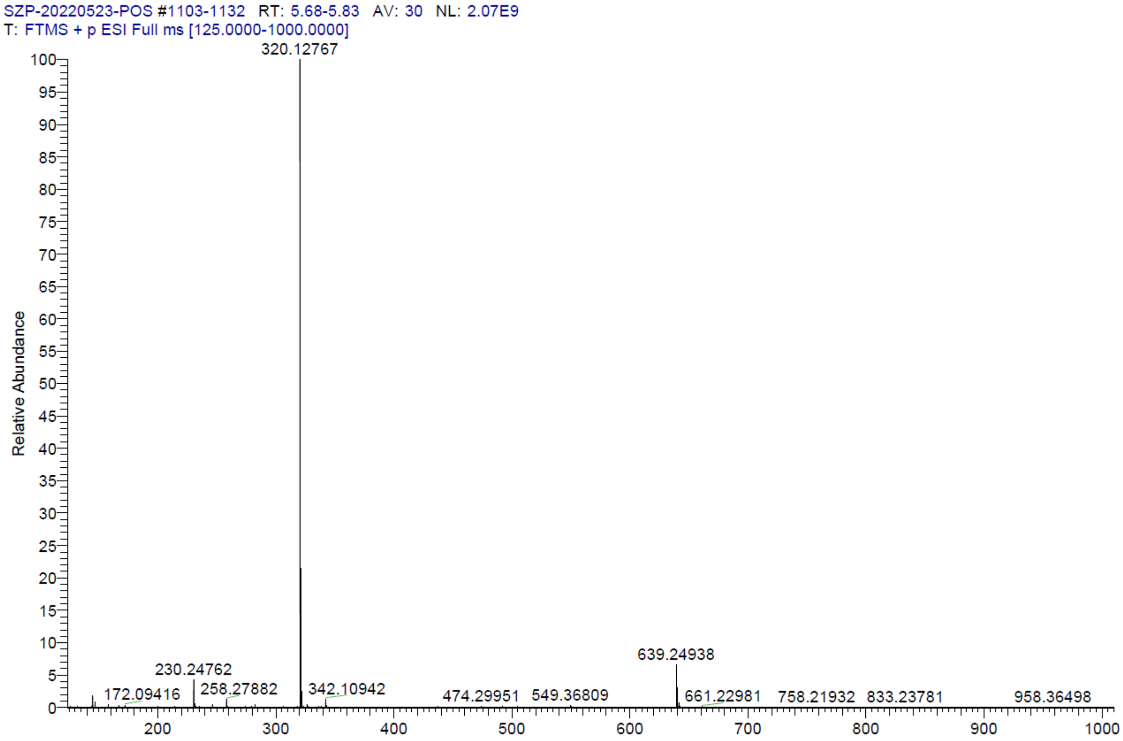


Figure S33. HRMS spectrum of **13b**

**(*Z*)-ethyl 2-(4-hydroxyquinolin-2-yl)-3-(4-nitrophenyl)acrylate (20)**

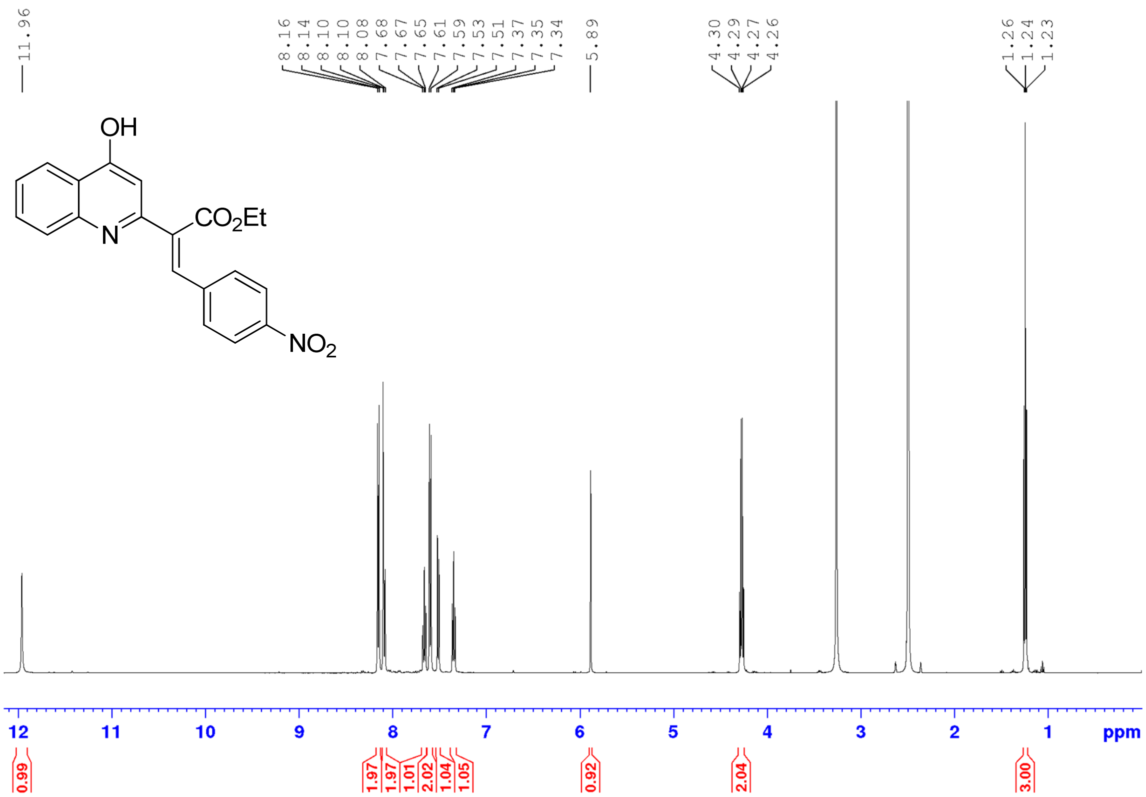


Figure S34. $^{1}$H NMR spectrum of **20**

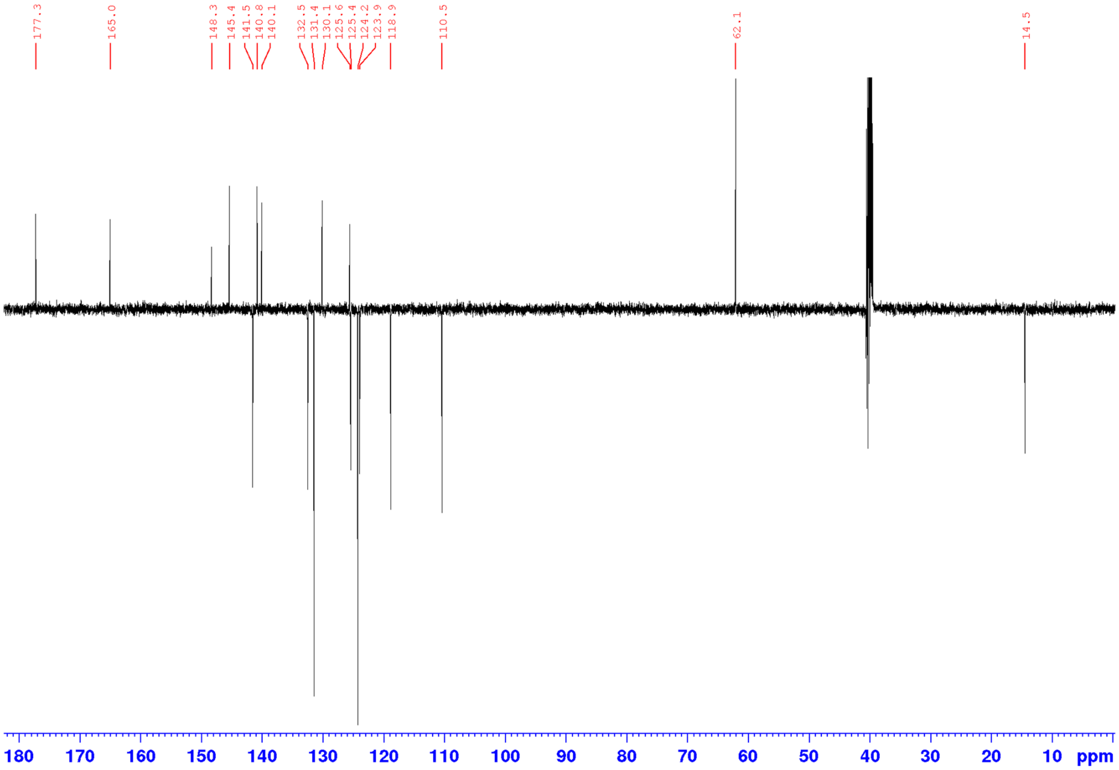


Figure S35. $^{13}$C NMR spectrum of **20**

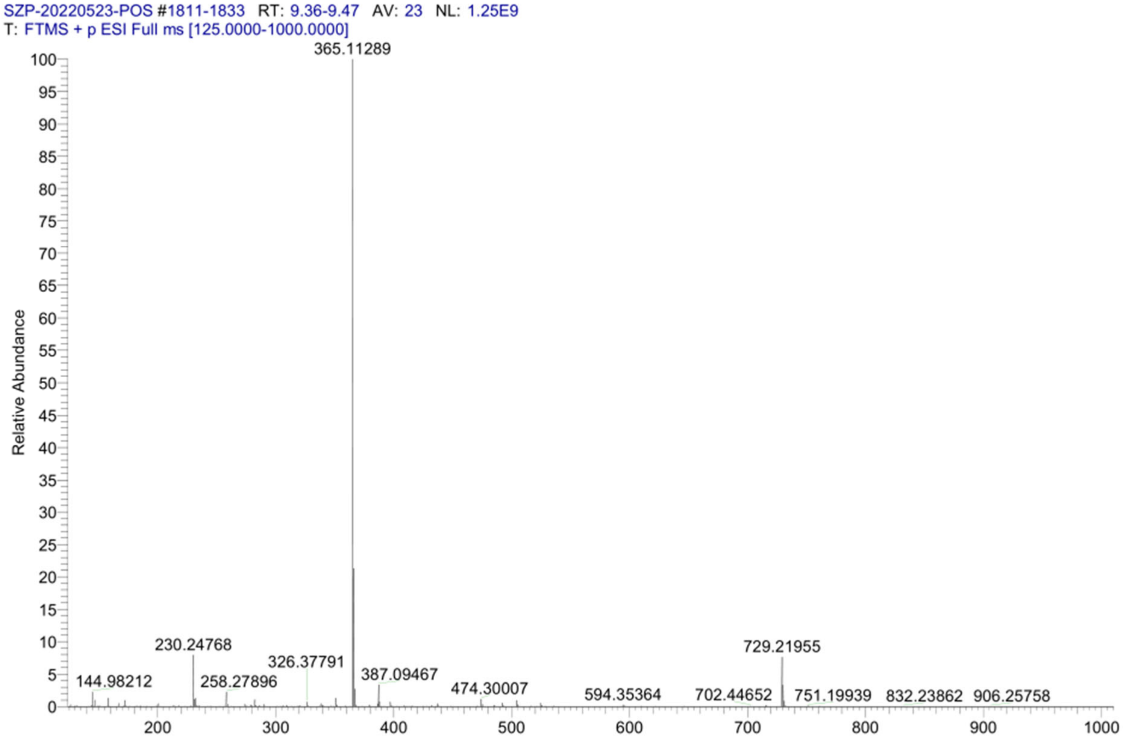


Figure S36. HRMS spectrum of **20**

**(*Z*)-ethyl 3-(4-fluorophenyl)-2-(4-hydroxyquinolin-2-yl)acrylate (21)**

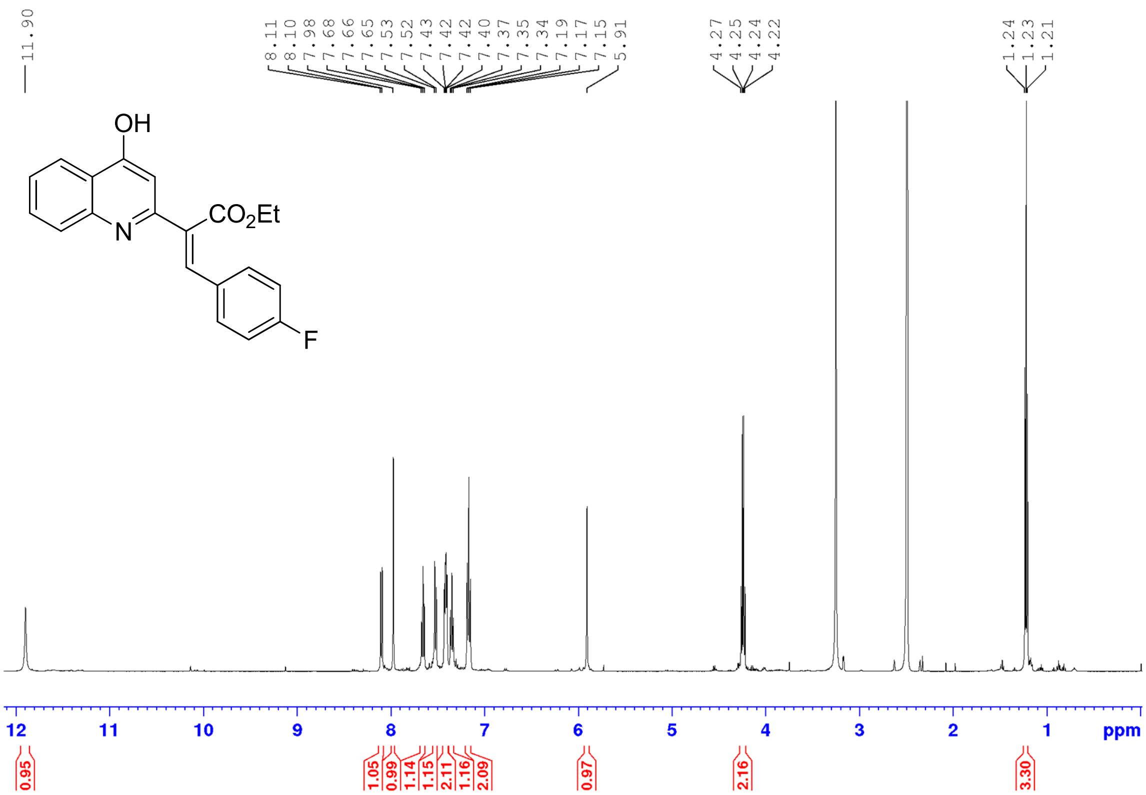

Figure S37. $^{1}H$ NMR spectrum of **21**

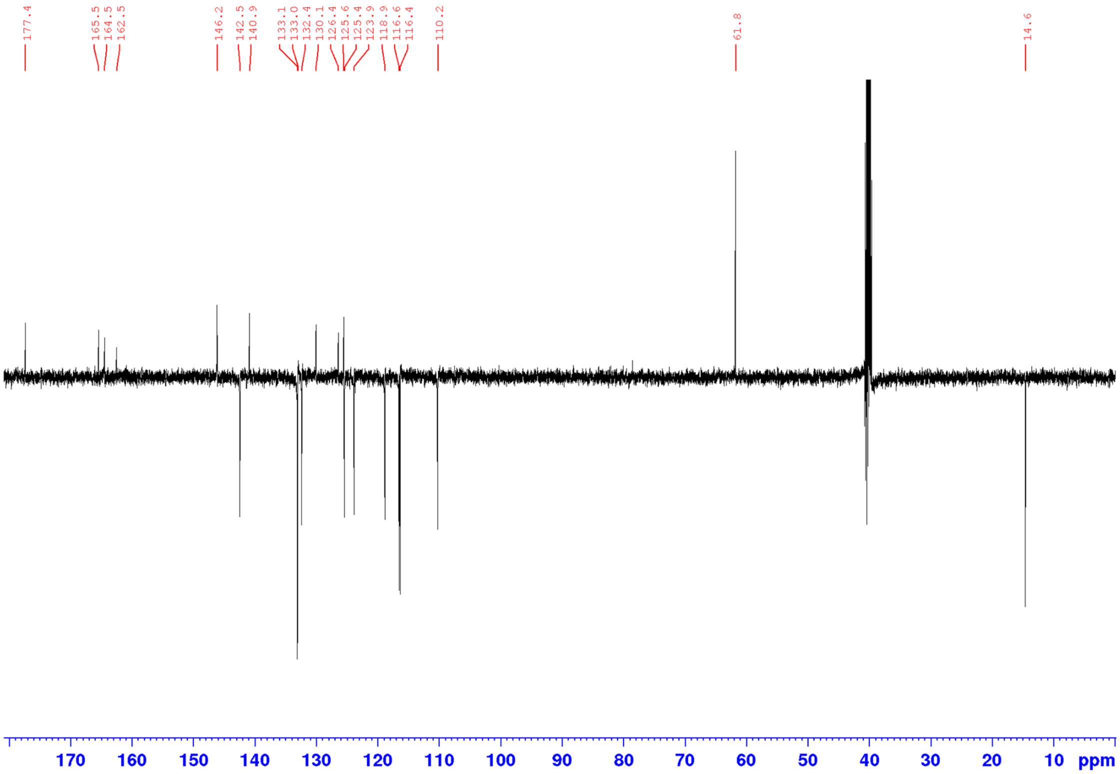

Figure S38. $^{13}C$ NMR spectrum of **21**

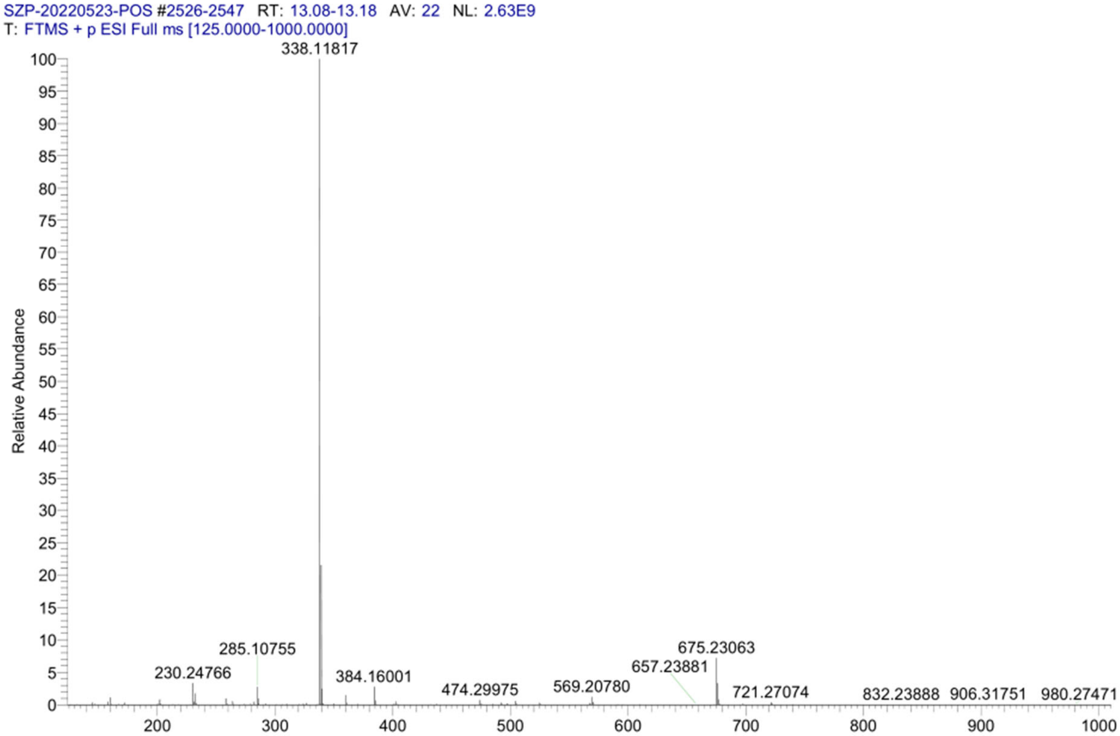


Figure S39. HRMS spectrum of **21**

**(*Z*)-ethyl 2-(4-hydroxyquinolin-2-yl)-3-(*p*-tolyl)acrylate (22)**

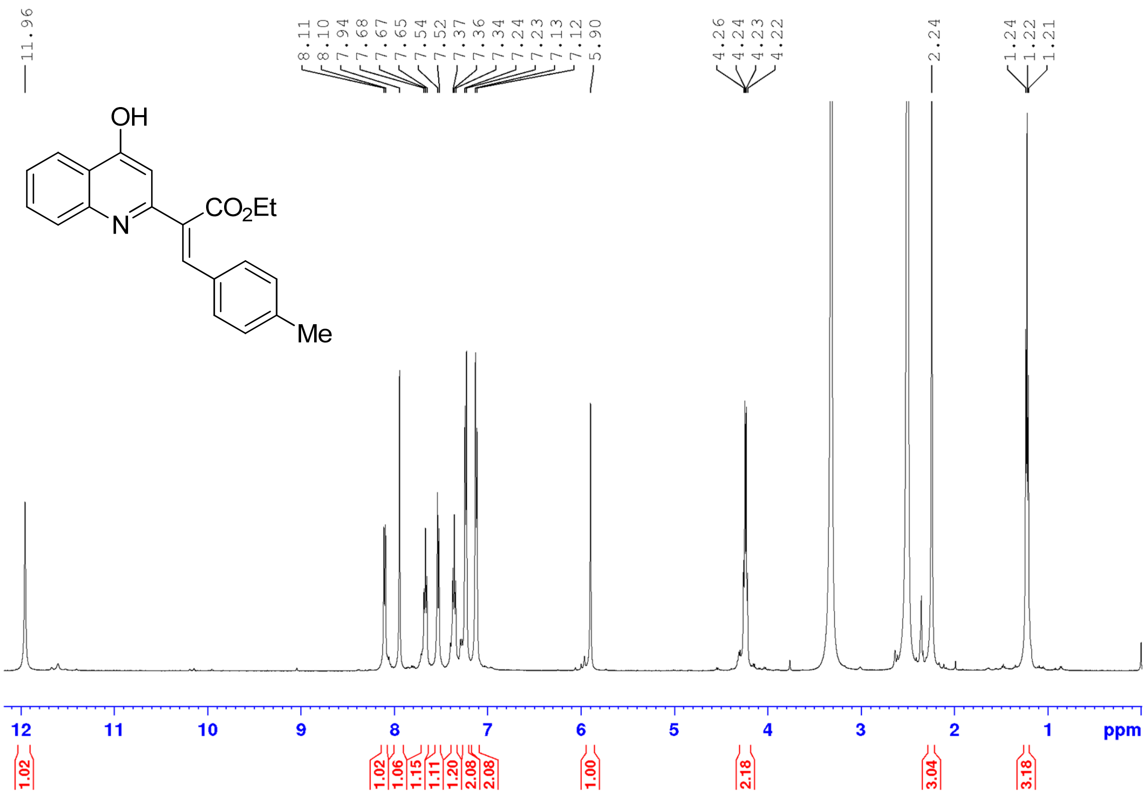


Figure S40. $^{1}$H NMR spectrum of **22**

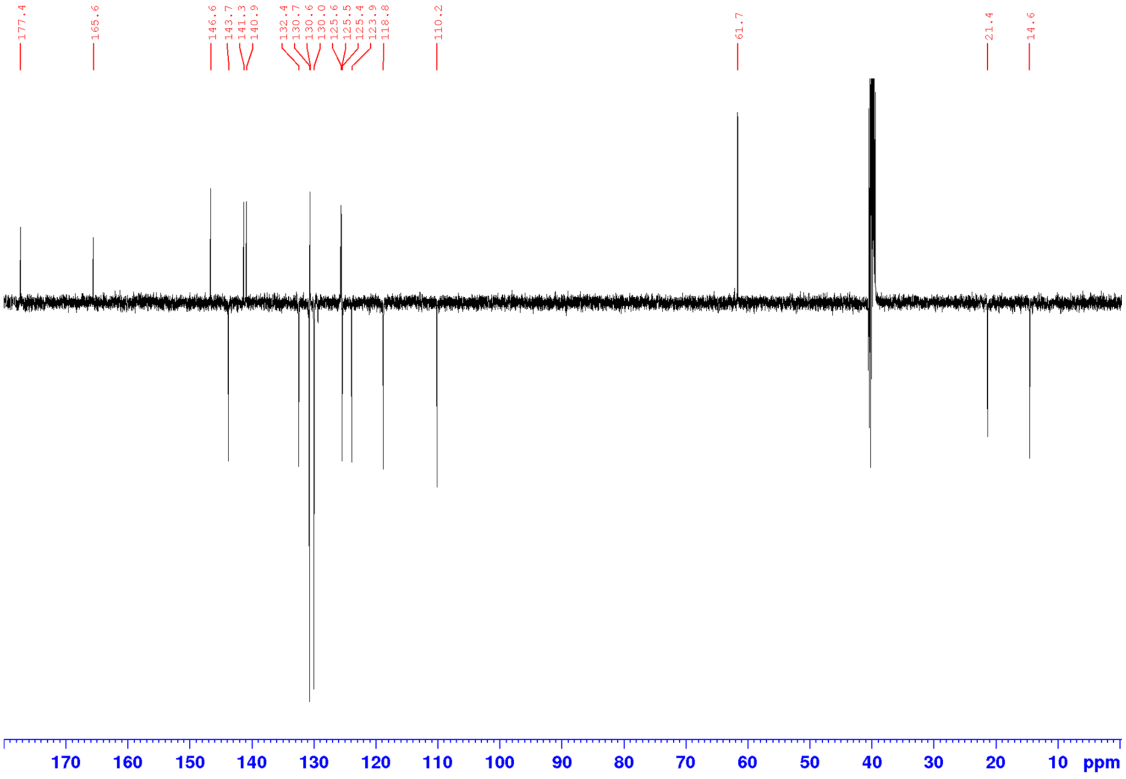


Figure S41. $^{13}$C NMR spectrum of **22**

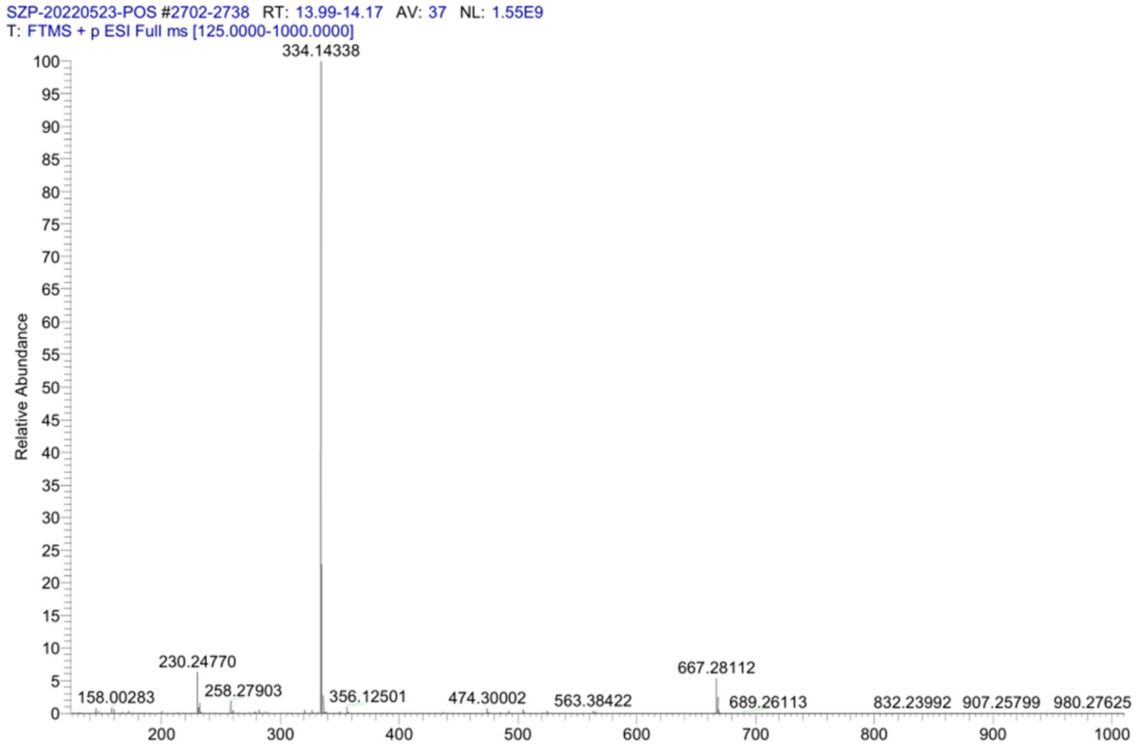


Figure S42. HRMS spectrum of **22**

**(*Z*)-ethyl 2-(4-hydroxyquinolin-2-yl)-3-(4-methoxyphenyl)acrylate (23)**

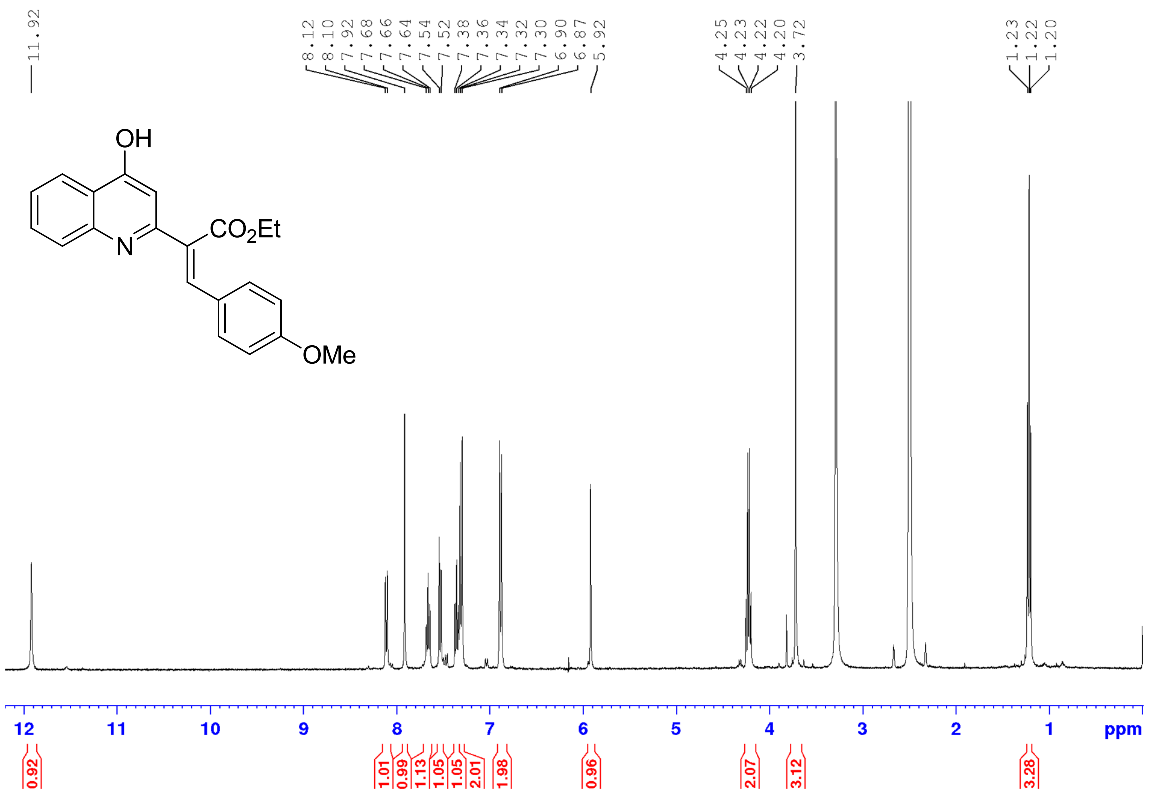


Figure S43. $^{1}$H NMR spectrum of **23**

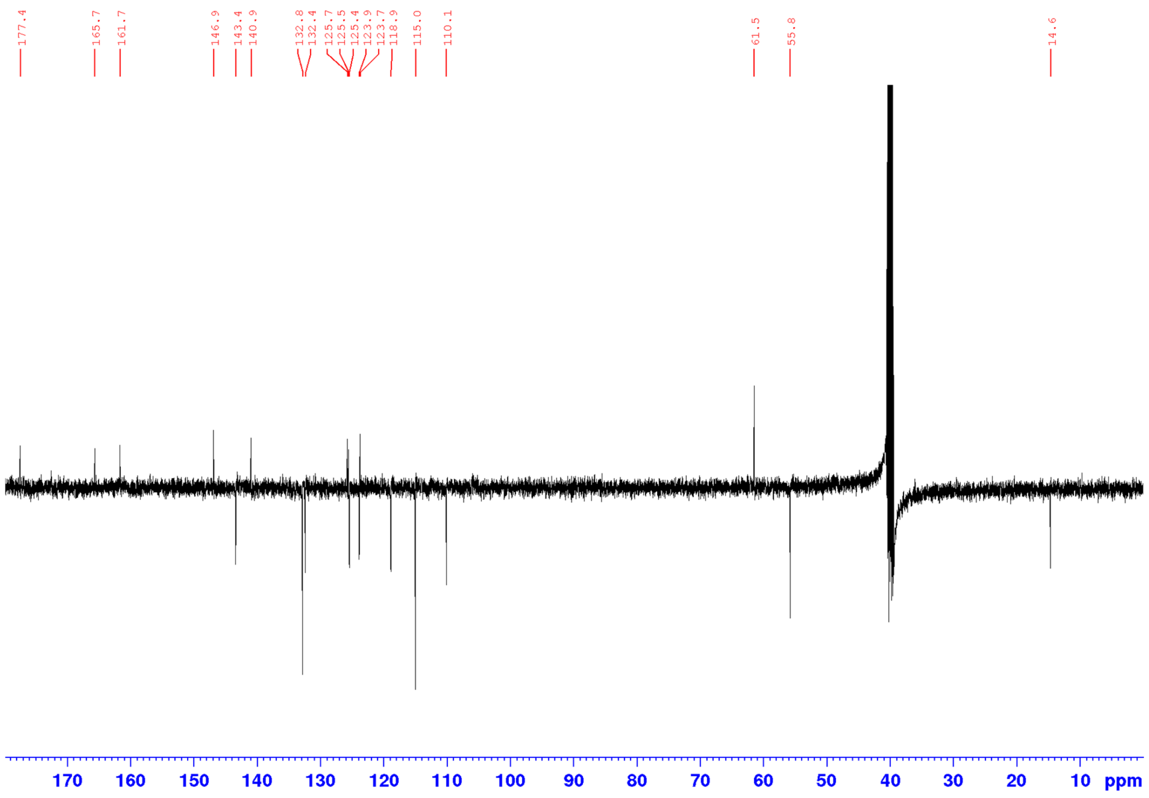


Figure S44. $^{13}$C NMR spectrum of **23**

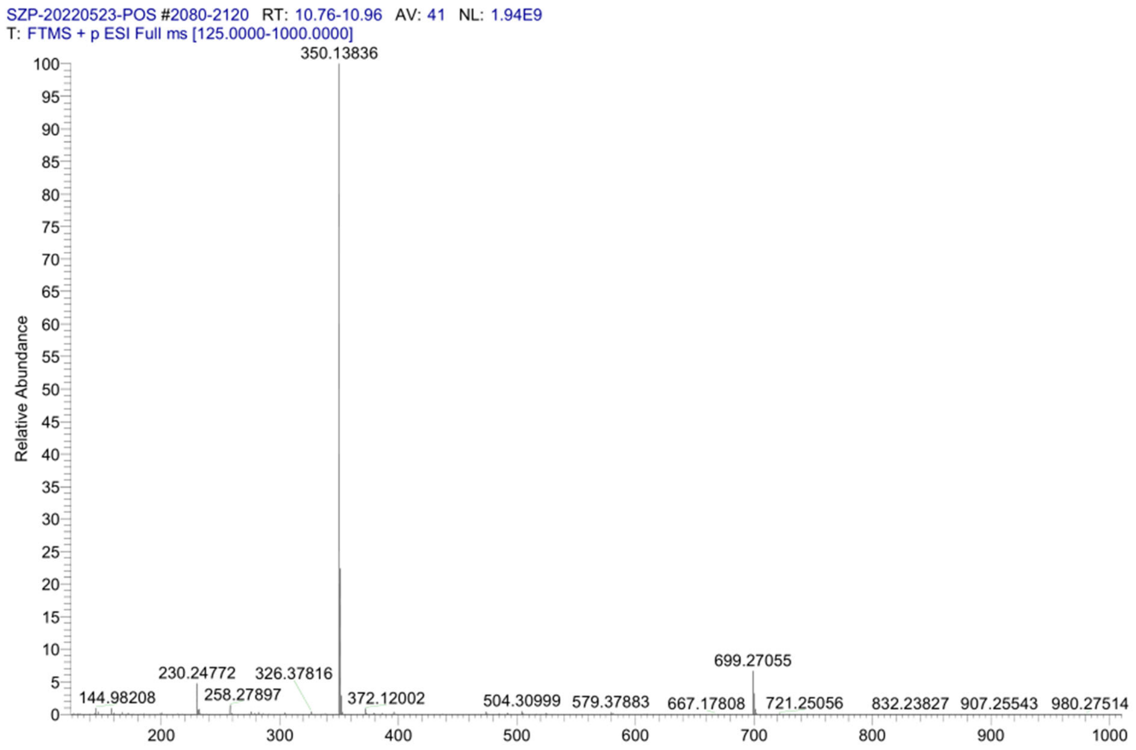


Figure S45. HRMS spectrum of **23**

**(*Z*)-ethyl 3-(4-(dimethylamino)phenyl)-2-(4-hydroxyquinolin-2-yl)acrylate (24)**

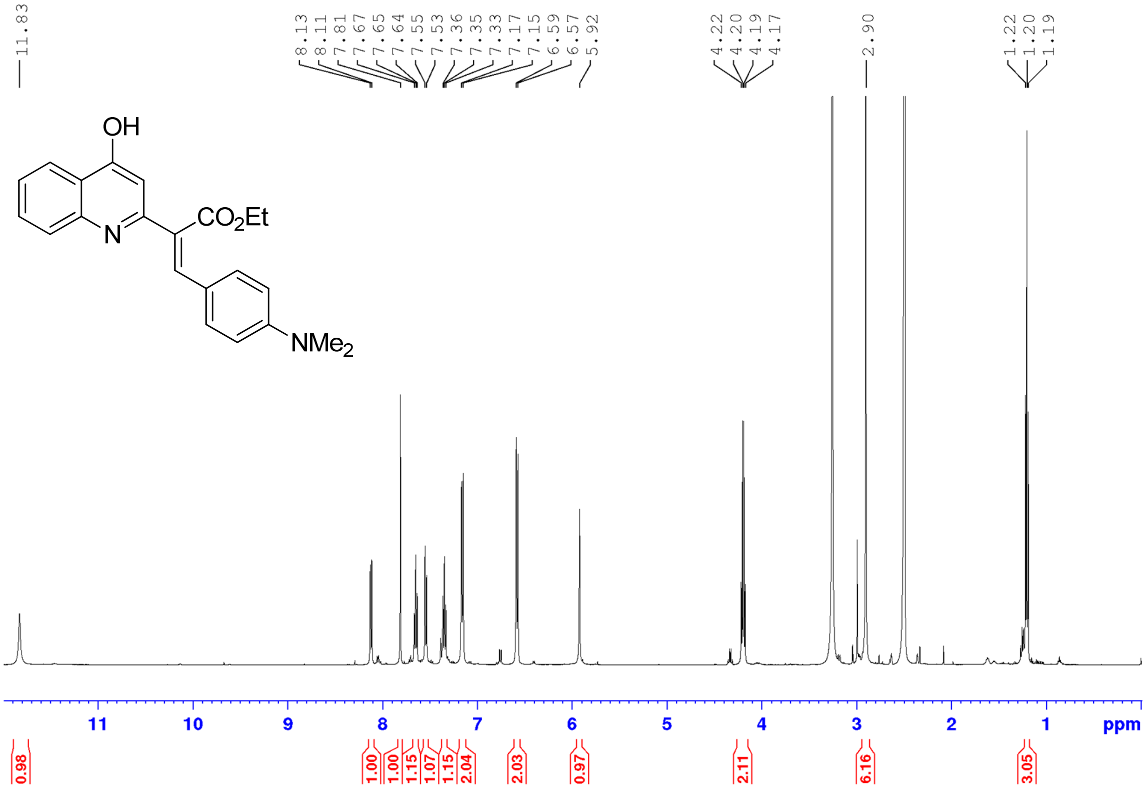


Figure S46. $^{1}$H NMR spectrum of **24**

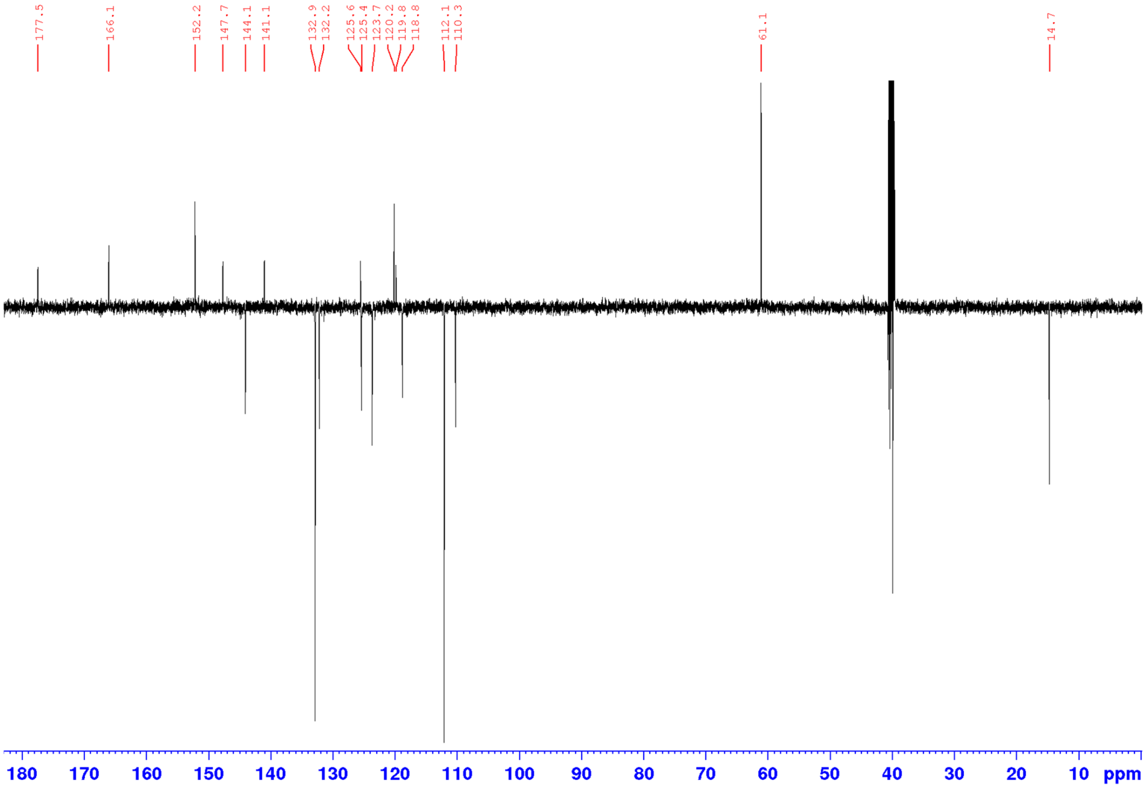


Figure S47. $^{13}$C NMR spectrum of **24**

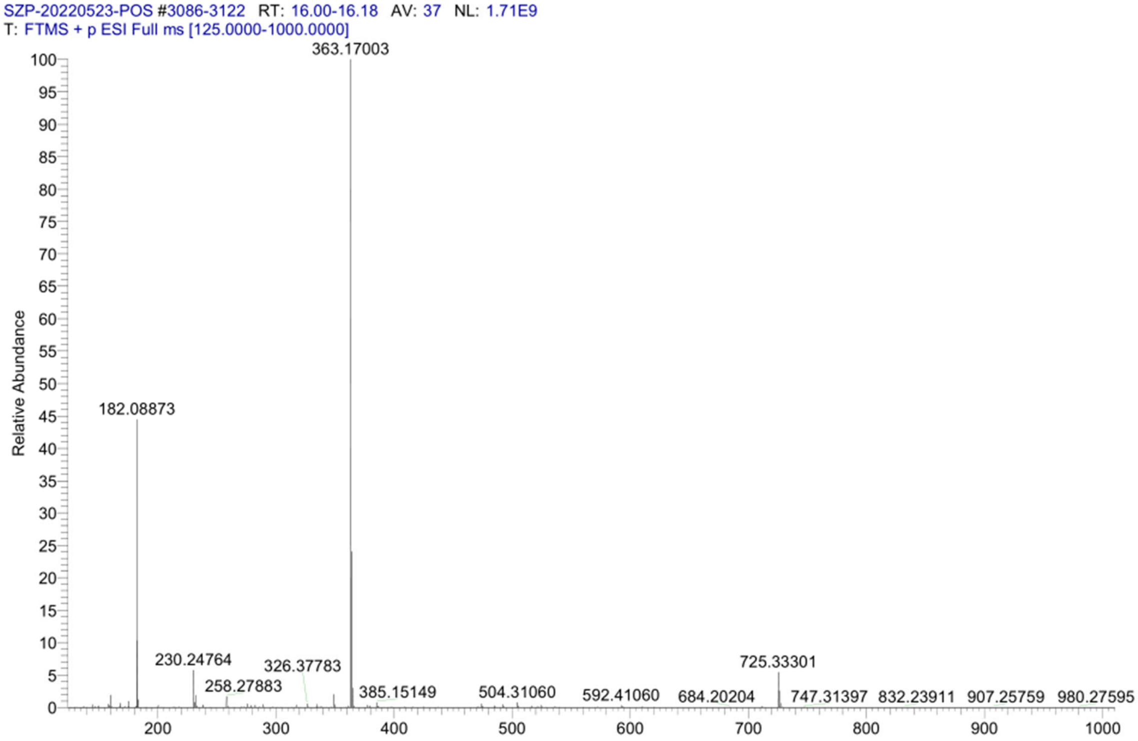


Figure S48. HRMS spectrum of **24**

**(*Z*)-ethyl 2-(4-hydroxyquinolin-2-yl)-3-(naphthalen-1-yl)acrylate (25)**

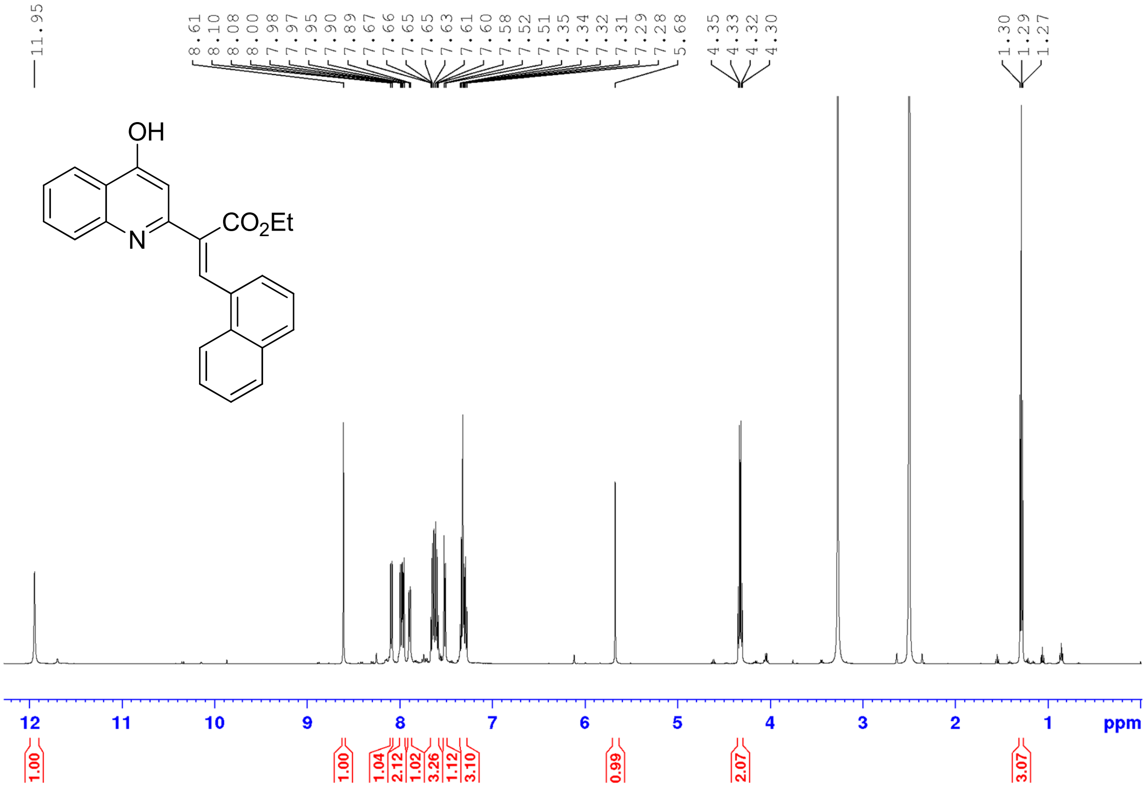


Figure S49. $^{1}$H NMR spectrum of **25**

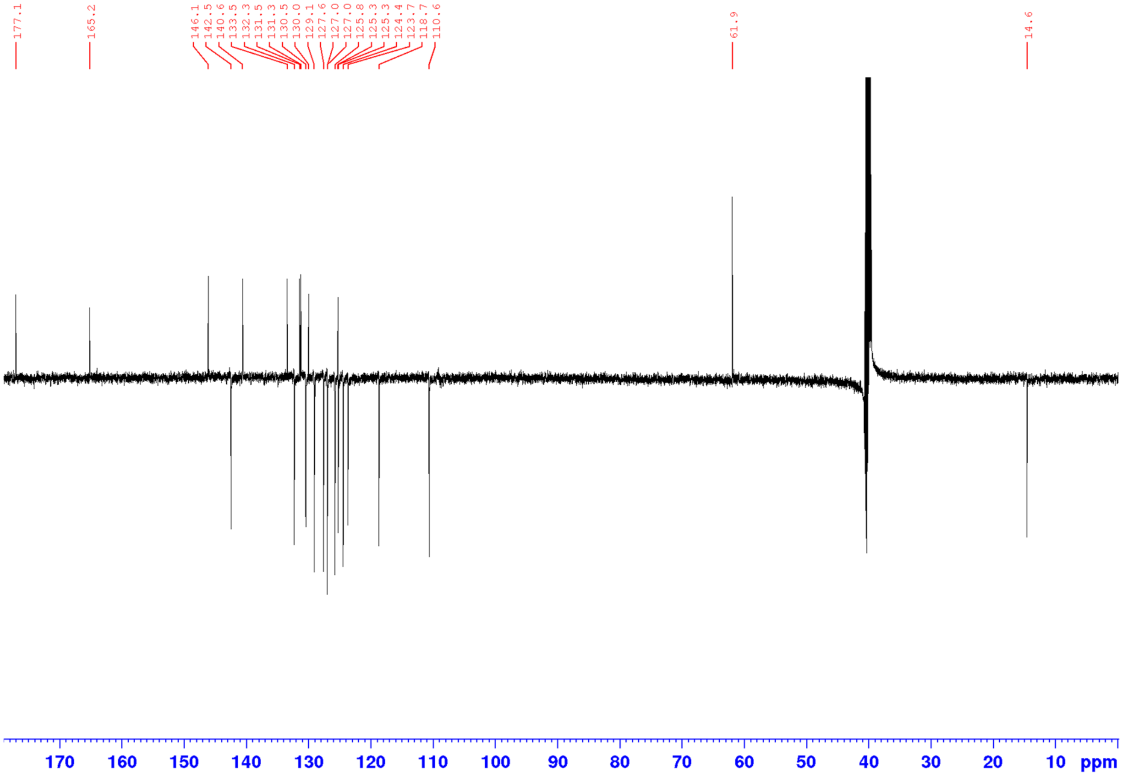


Figure S50. $^{13}$C NMR spectrum of **25**

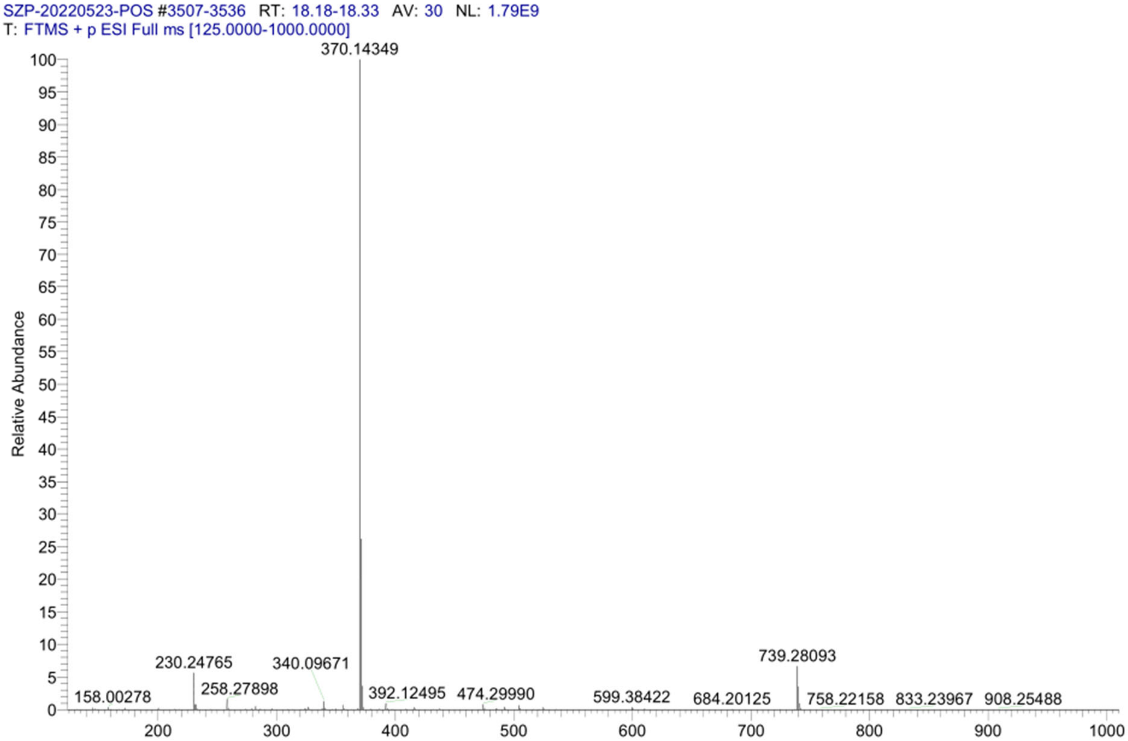


Figure S51. HRMS spectrum of **25**

**(*Z*)-ethyl 2-(4-hydroxyquinolin-2-yl)-3-(naphthalen-2-yl)acrylate (26)**

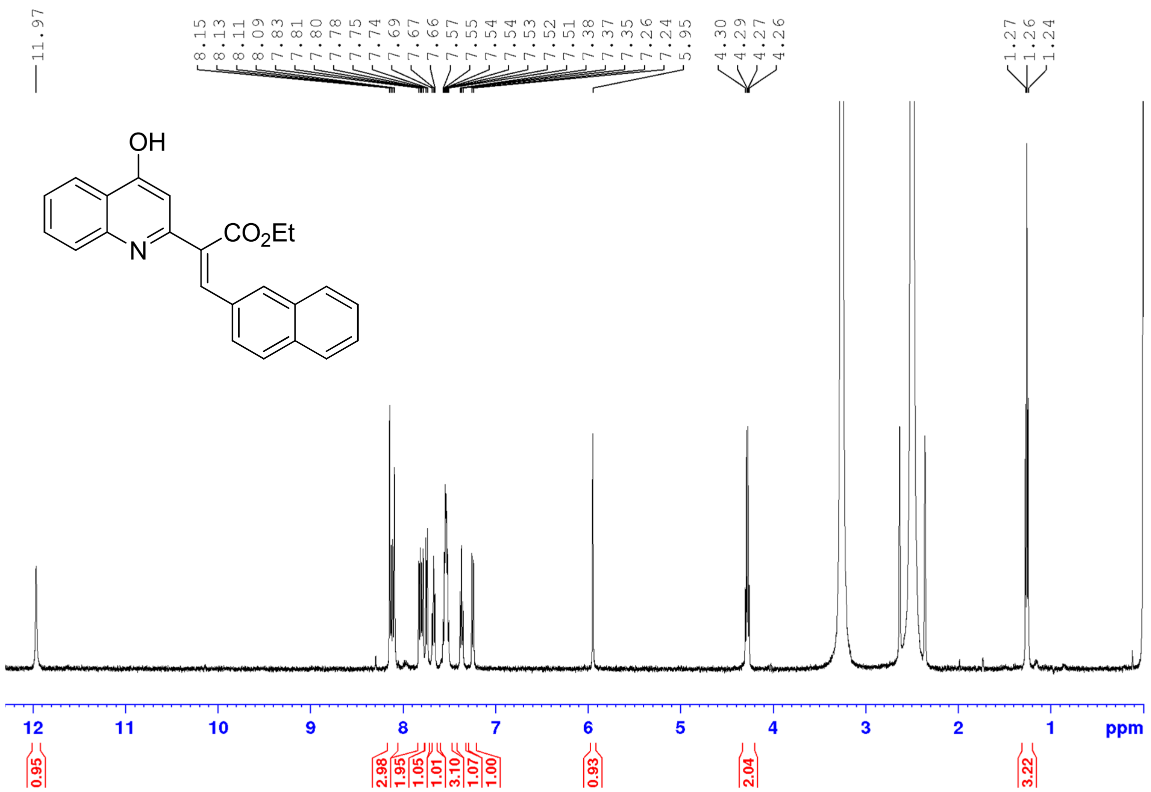


Figure S52. $^{1}$H NMR spectrum of **26**

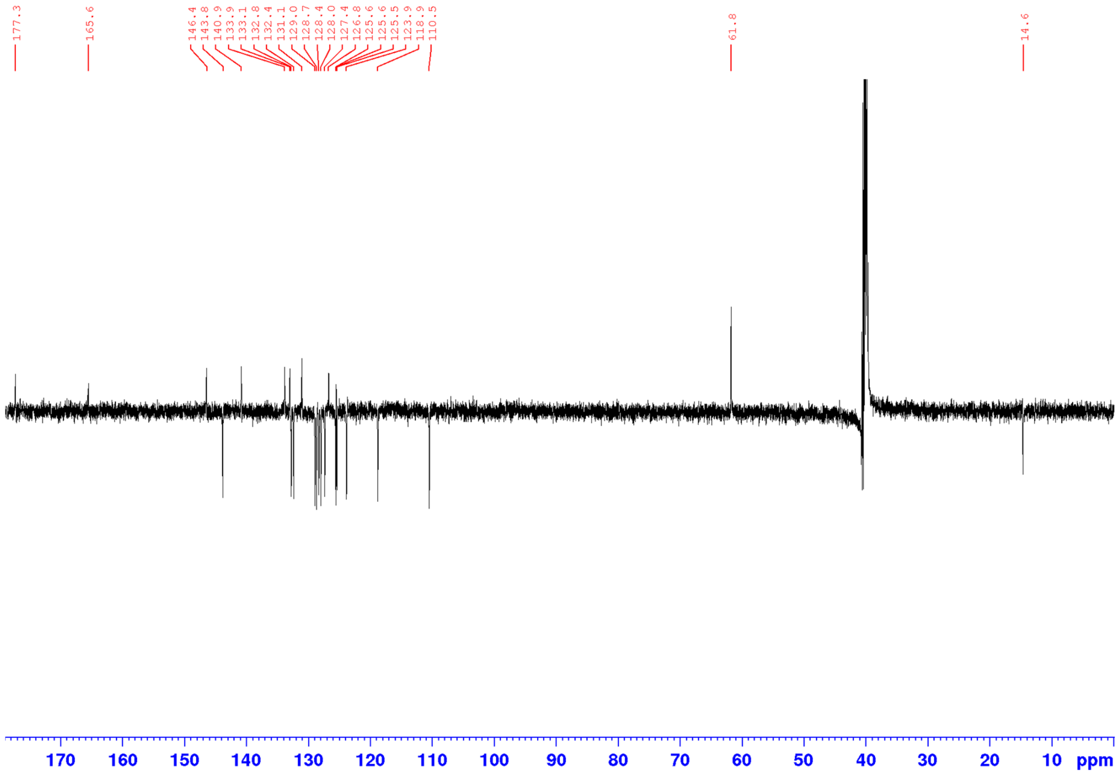


Figure S53. $^{13}$C NMR spectrum of **26**

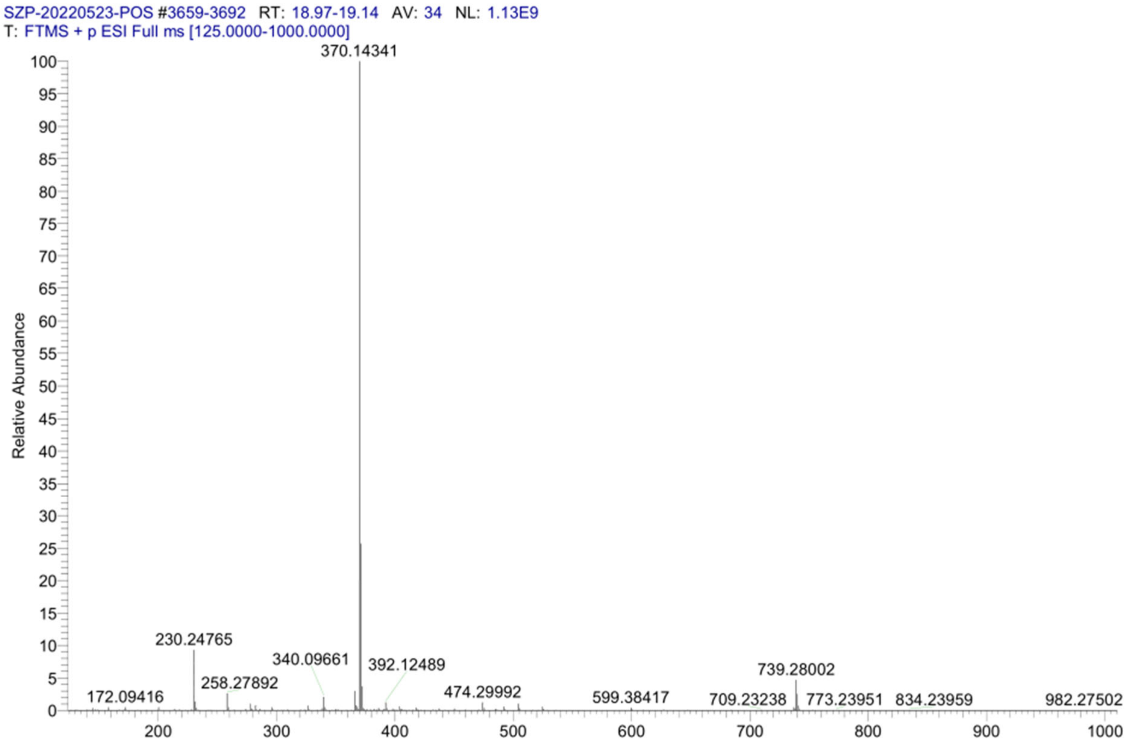


Figure S54. HRMS spectrum of **26**

**3-(4-Hydroxyquinolin-2-yl)-*2H*-chromen-2-one (28)**

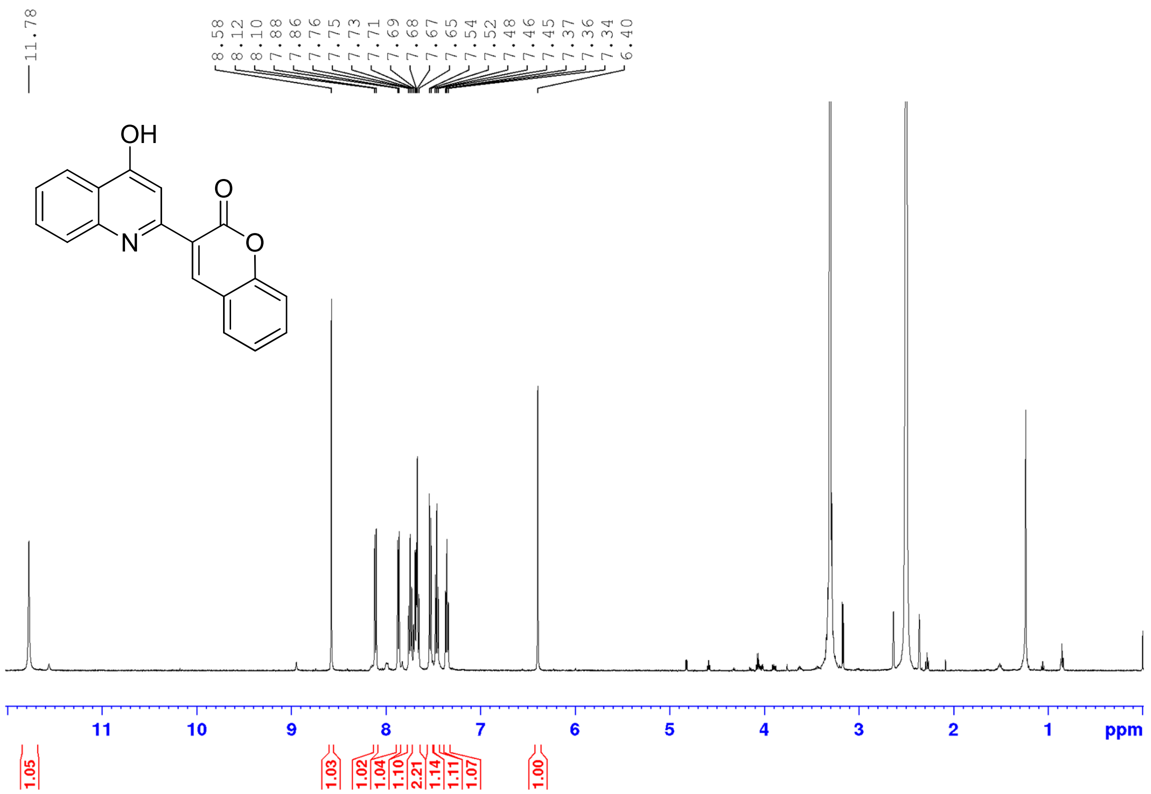


Figure S55. $^{1}$H NMR spectrum of **28**

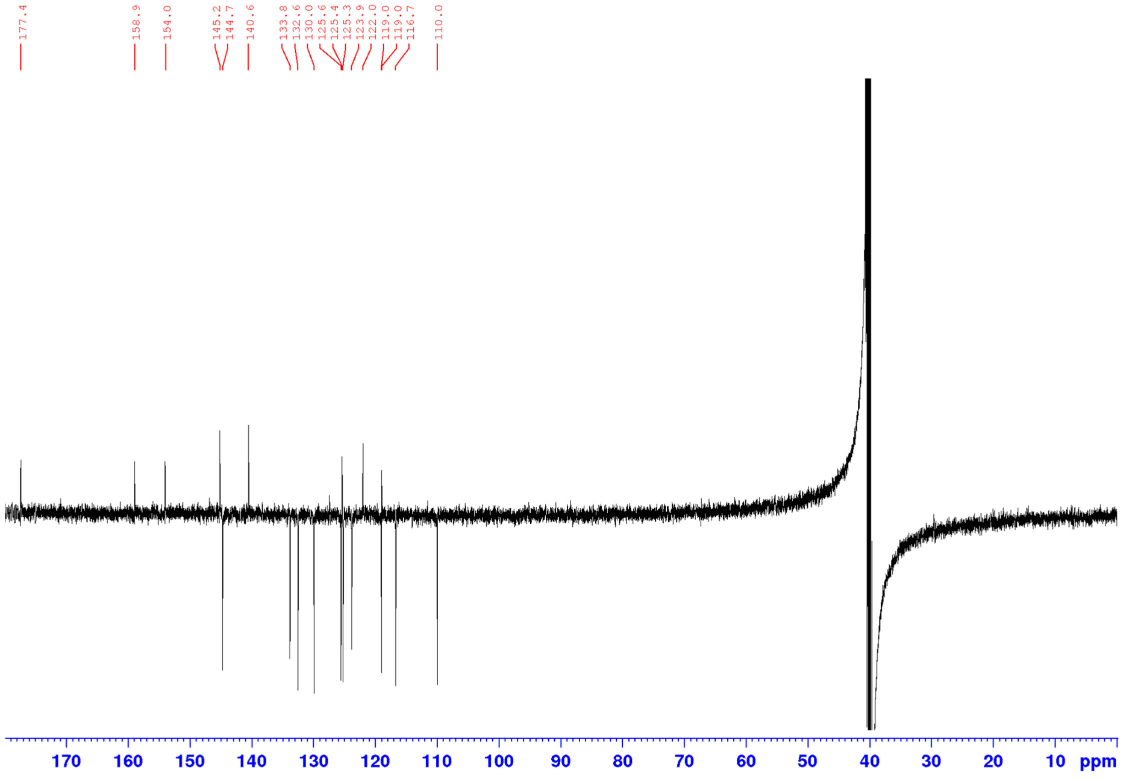


Figure S56. $^{13}$C NMR spectrum of **28**

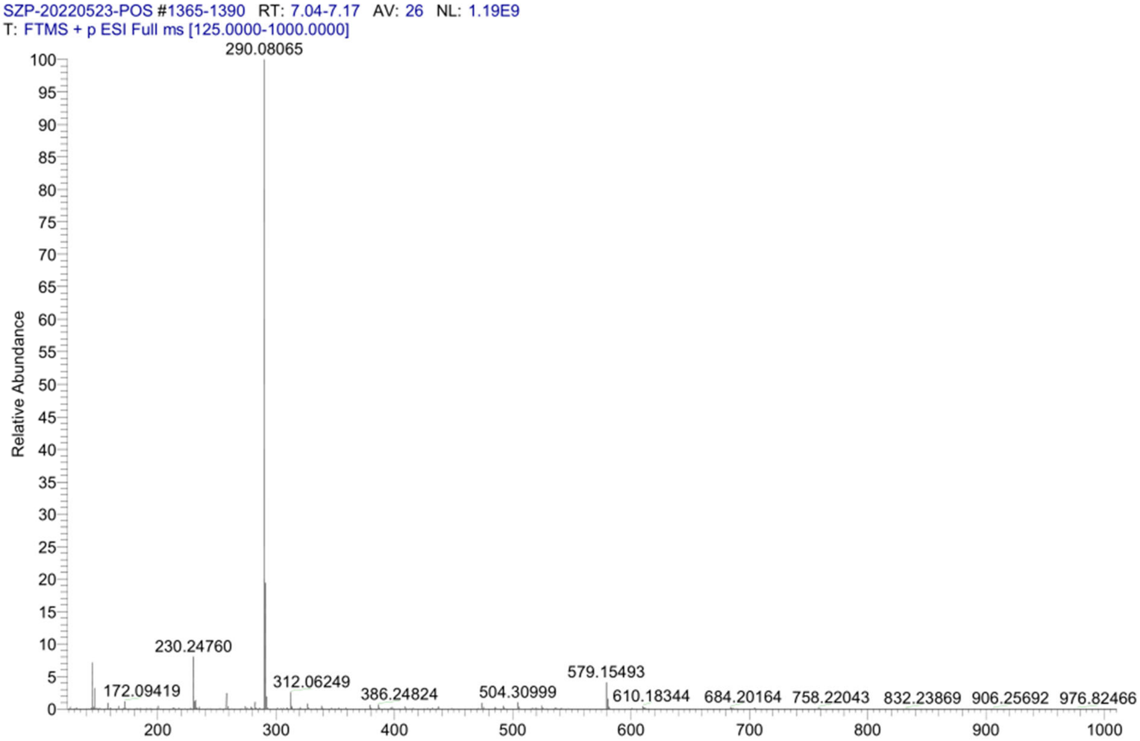


Figure S57. HRMS spectrum of **28**

**2-(4-Hydroxyquinolin-2-yl)-*3H*-benzo[*f*]chromen-3-one (29)**

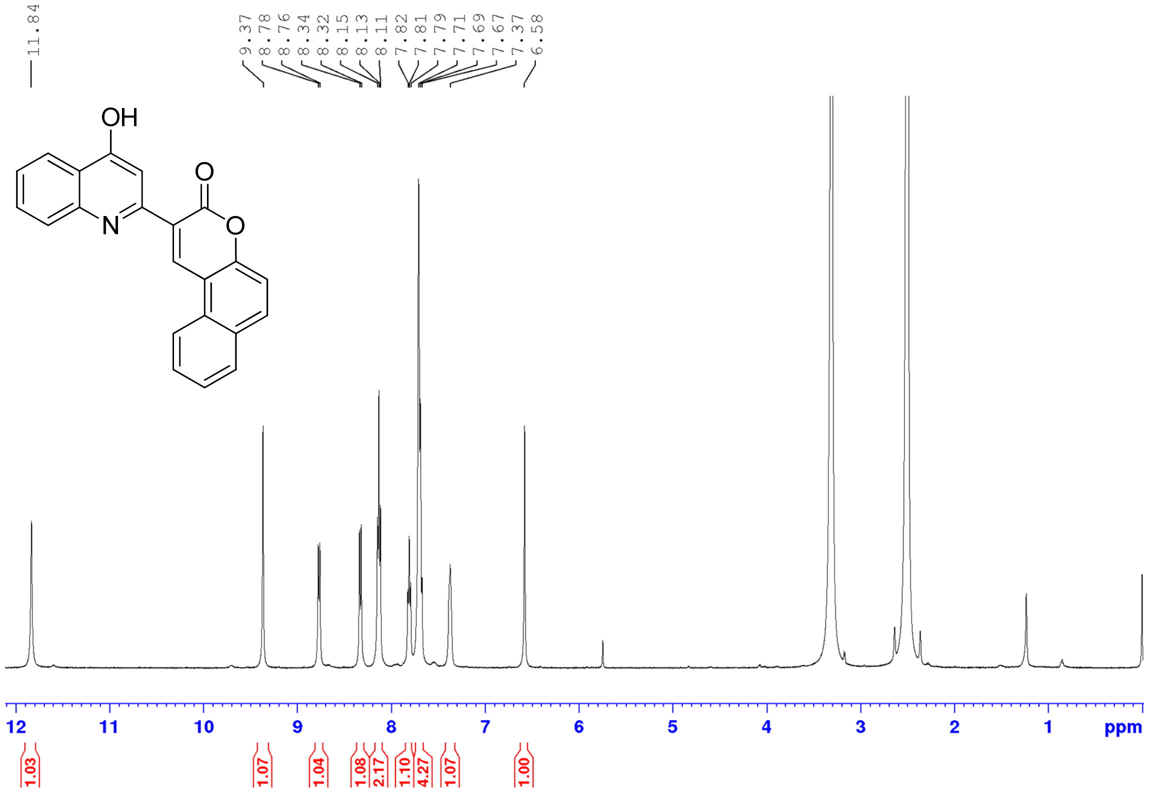


Figure S58. $^{1}$H NMR spectrum of **29**

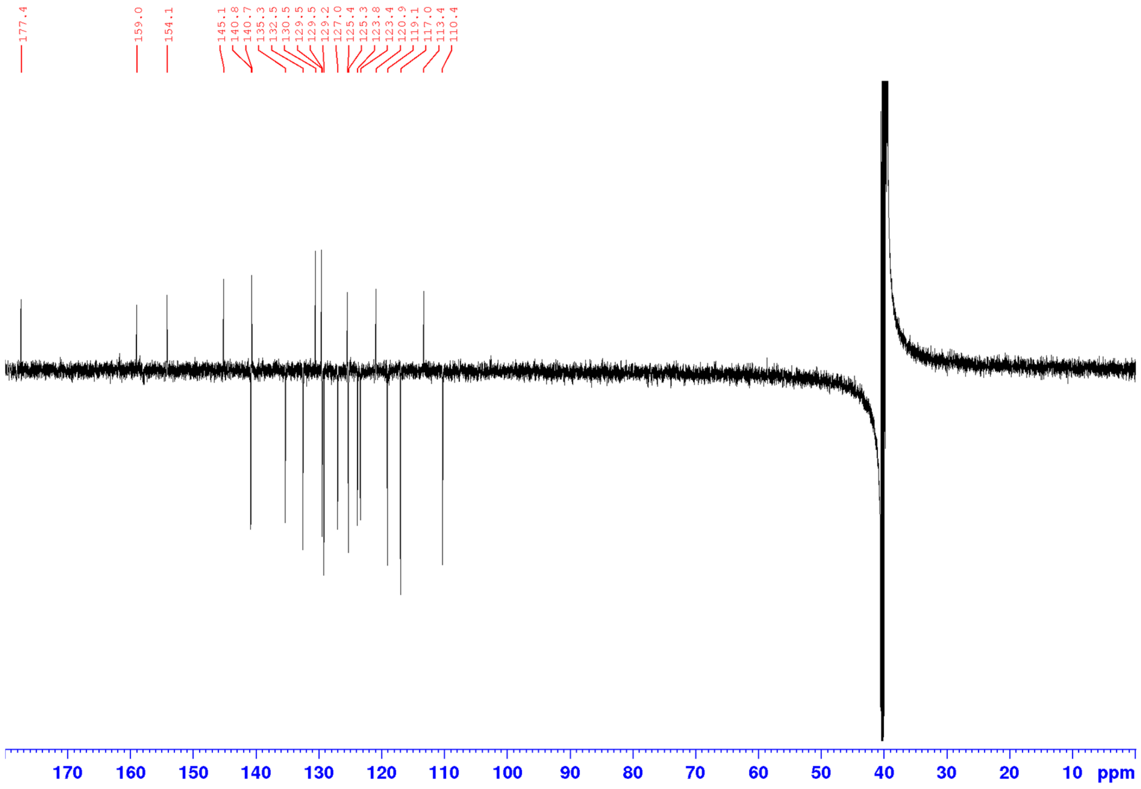


Figure S59. $^{13}$C NMR spectrum of **29**

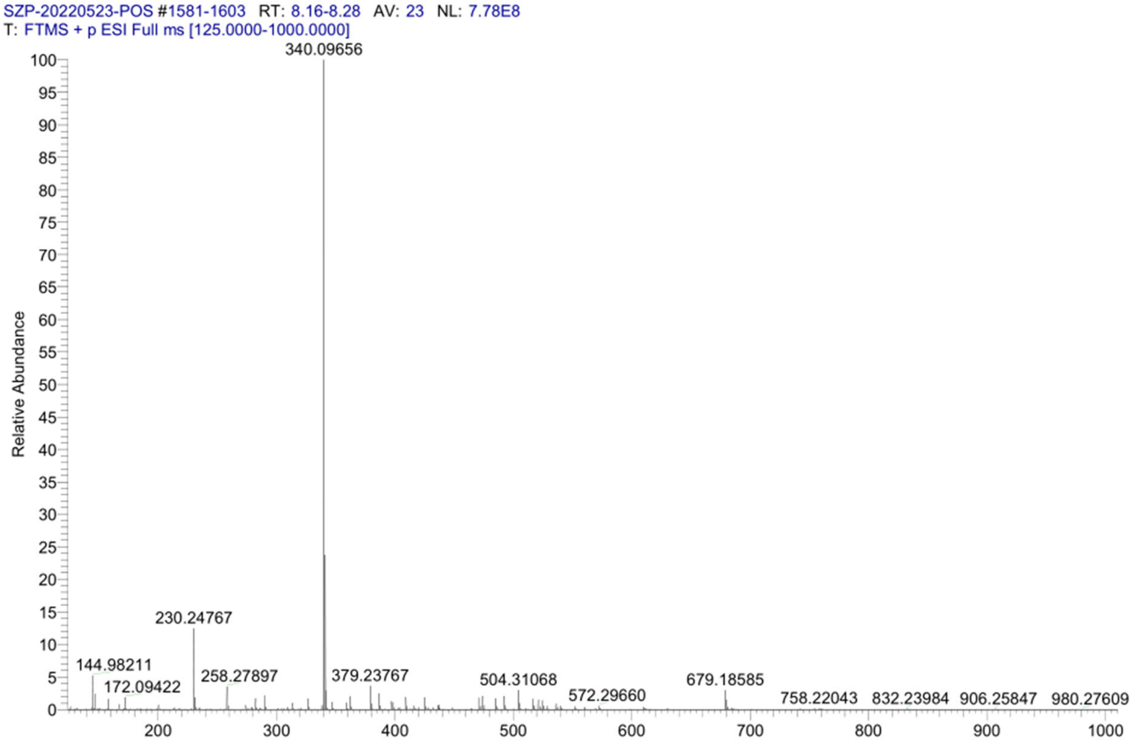


Figure S60. HRMS spectrum of **29**

**3-(4-Hydroxyquinolin-2-yl)-*2H*-benzo[*h*]chromen-2-one (30)**

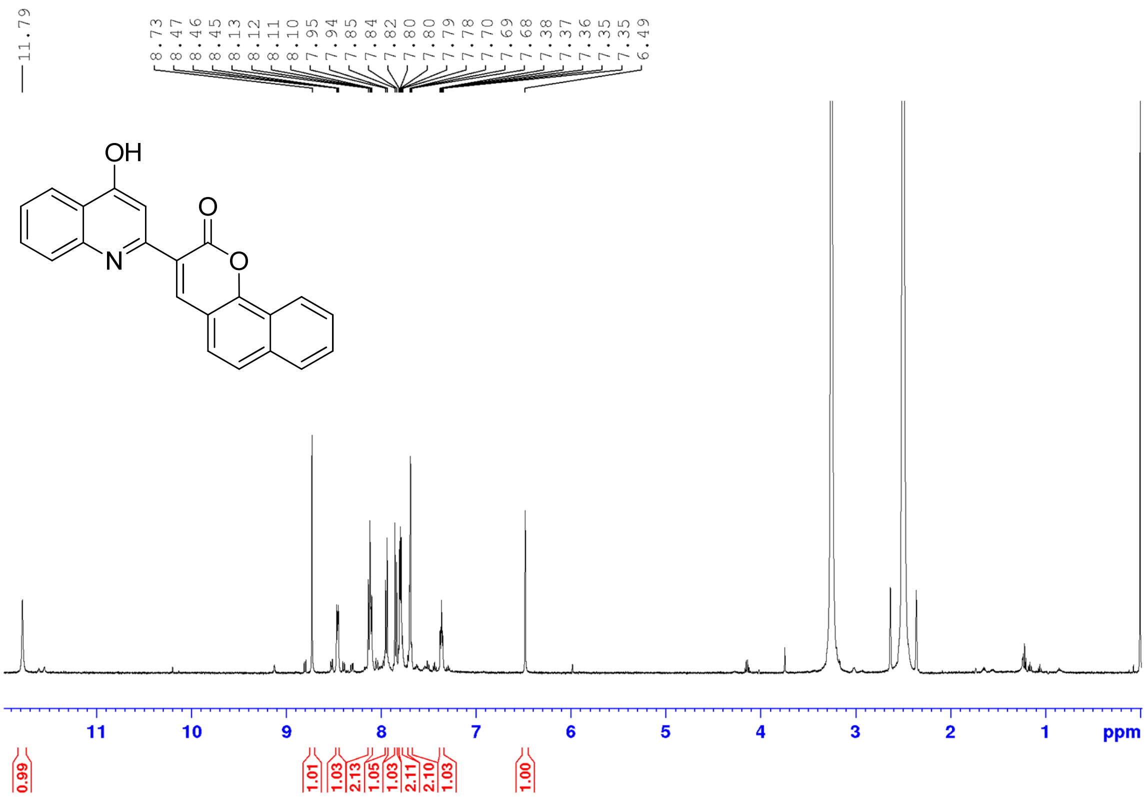


Figure S61. $^{1}$H NMR spectrum of **30**

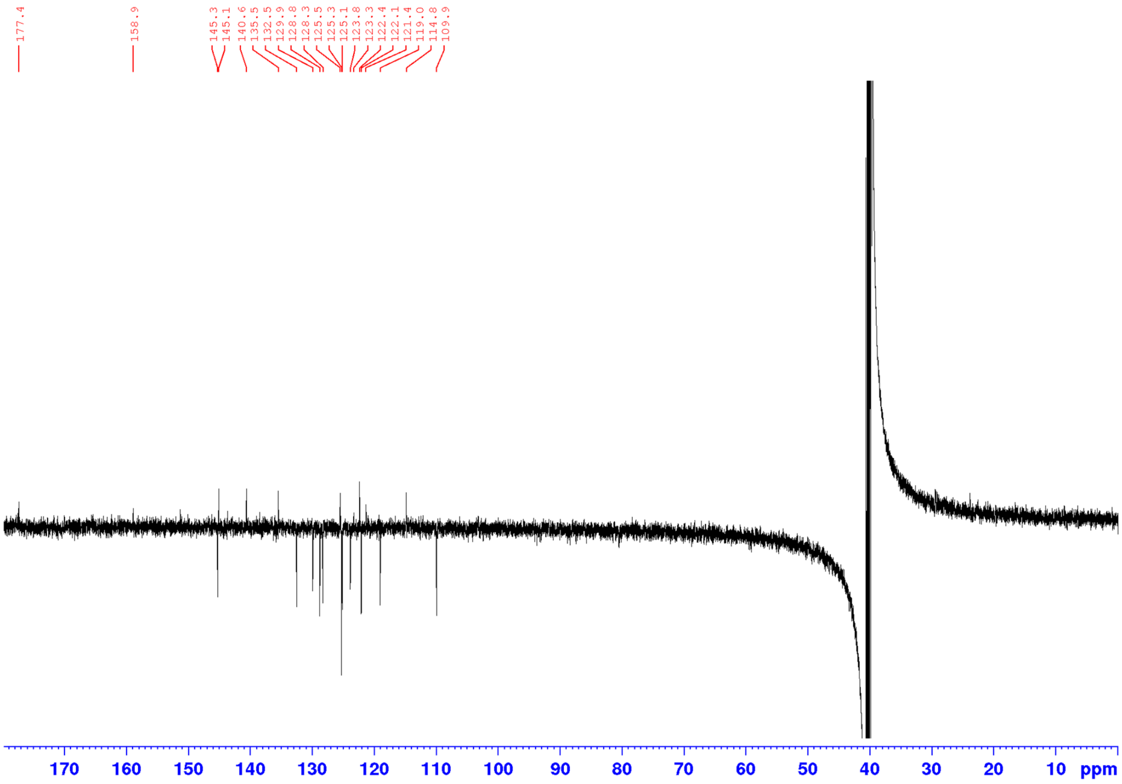


Figure S62. $^{13}$C NMR spectrum of **30**

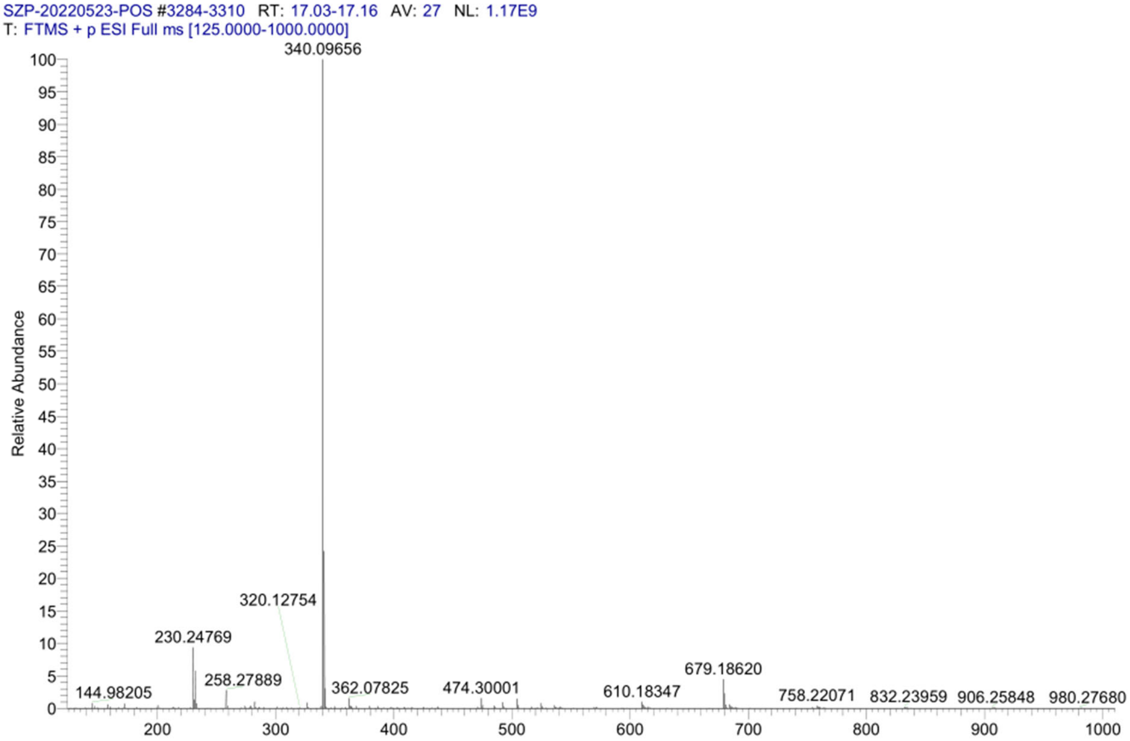

Figure S63. HRMS spectrum of **30**

**2-(4-Hydroxyquinolin-2-yl)-3-phenylacrylic acid (14)**

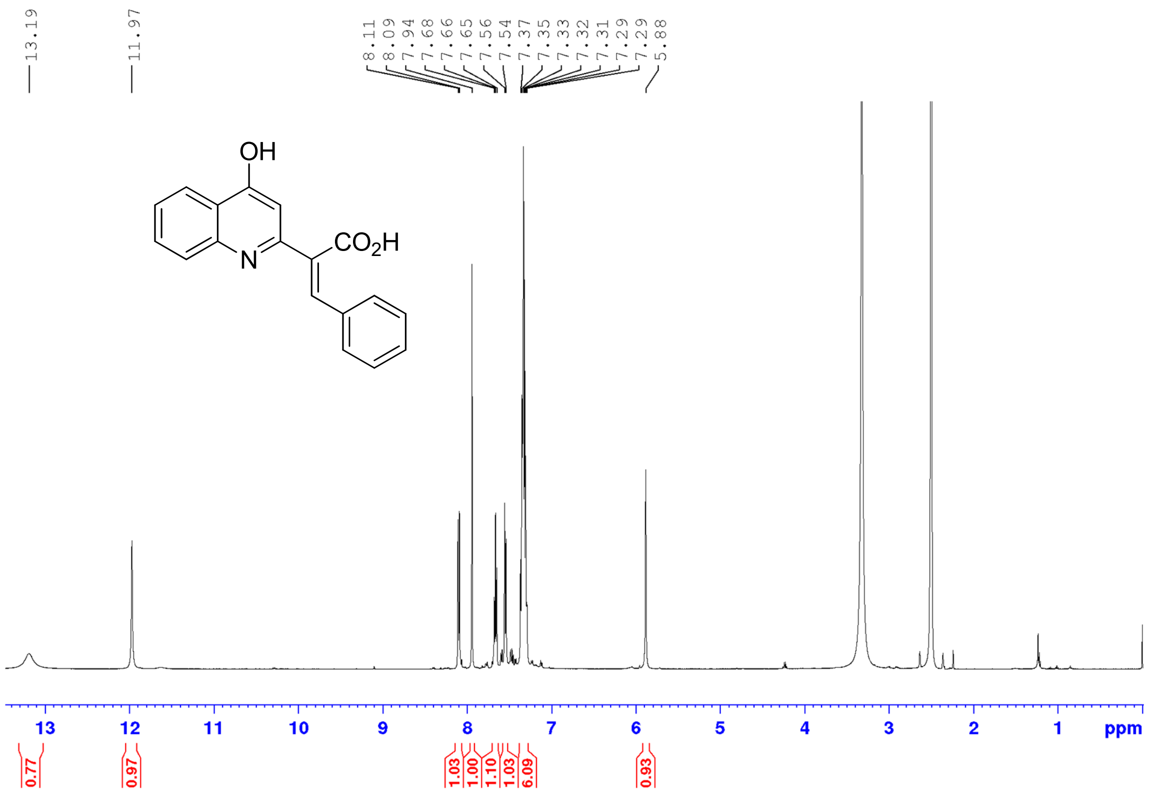


Figure S64. $^{1}$H NMR spectrum of **14**

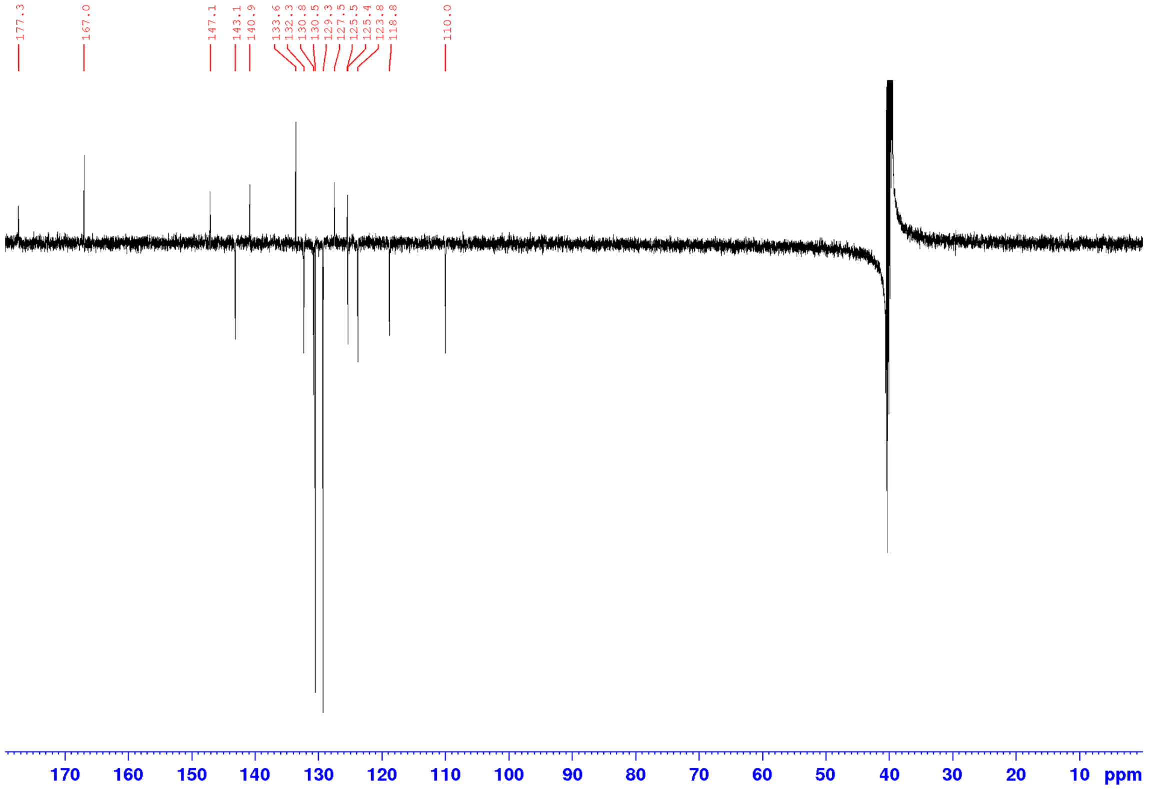


Figure S65. $^{13}$C NMR spectrum of **14**

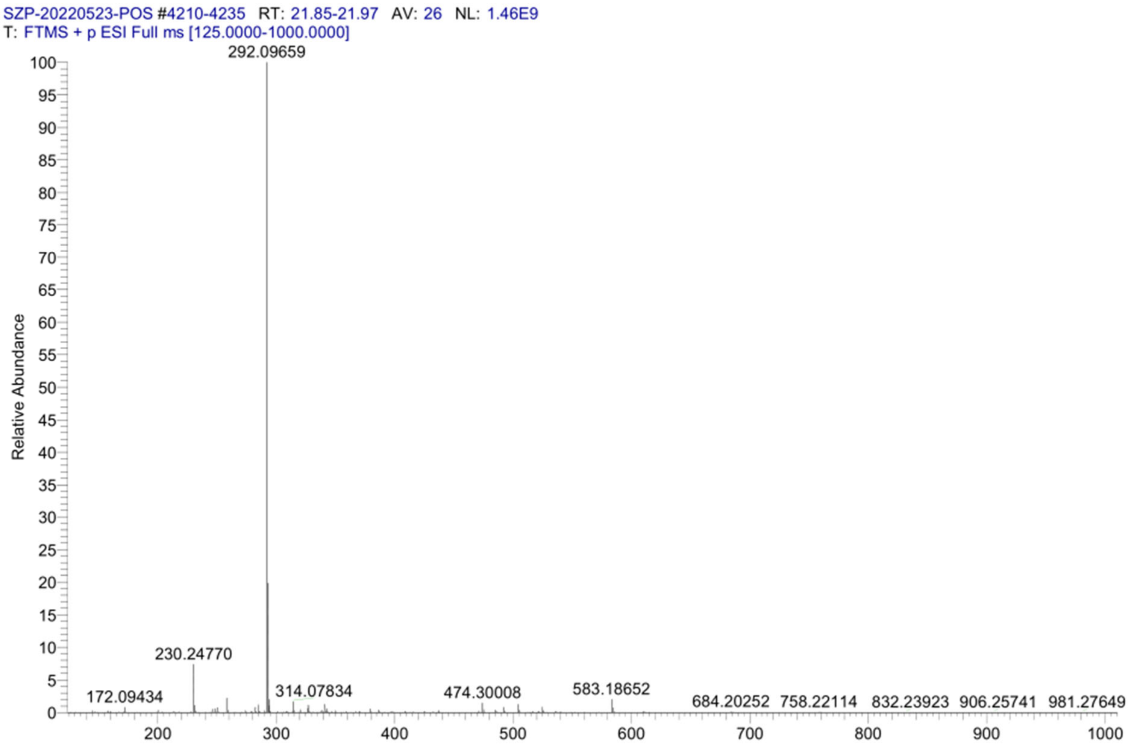


Figure S66. HRMS spectrum of **14**
